# Multivitamins After Myocardial Infarction in Patients With Diabetes: A Randomized Clinical Trial

**DOI:** 10.1001/jamainternmed.2024.8408

**Published:** 2025-03-03

**Authors:** Francisco Ujueta, Gervasio A. Lamas, Kevin J. Anstrom, Ana Navas-Acien, Robin Boineau, Yves Rosenberg, Mario Stylianou, Teresa L. Z. Jones, Bonnie R. Joubert, Qilu Yu, Jun Wen, Hayley Nemeth, Zhen Huang, Vivian Fonseca, David M. Nathan, Gabriel Uwaifo, Ivan A. Arenas, Lan Luo, Jeffrey Baker, Diana Visentin, Andre Paixao, John F. Schmedtje, Daniel B. Mark

**Affiliations:** 1 Brigham and Women’s Hospital Division of Cardiovascular Medicine, Boston, Massachusetts; 2 Columbia University Division of Cardiology, Mount Sinai Medical Center, Miami Beach, Florida; 3 Gillings School of Global Public Health, University of North Carolina, Chapel Hill; 4 Department of Environmental Health Sciences, Columbia University Mailman School of Public Health, New York, New York; 5 National Center for Complementary and Integrative Health, National Institutes of Health, Bethesda, Maryland; 6 National Heart, Lung and Blood Institute, National Institutes of Health, Bethesda, Maryland; 7 National Institute of Diabetes and Digestive and Kidney Diseases, National Institutes of Health, Bethesda, Maryland; 8 National Institute of Environmental Health Sciences, National Institutes of Health, Durham, North Carolina; 9 Duke Clinical Research Institute, Duke University, Durham, North Carolina; 10 Tulane University School of Medicine, New Orleans, Louisiana; 11 Massachusetts General Hospital Diabetes Research Center, Harvard Medical School, Boston; 12 Southern Illinois University School of Medicine, Springfield; 13 Salem Health, Salem, Oregon; 14 Central Florida Heart Center, Ocala; 15 Clinical Research Prime, Idaho Falls, Idaho; 16 South Simcoe Cardiac Services, Barrie, Ontario, Canada; 17 Arkansas Heart Hospital, Little Rock; 18 Roanoke Heart Institute, Roanoke, Virginia

## Abstract

**Importance:**

In 2013, the Trial to Assess Chelation Therapy (TACT) reported that in 1708 patients with stable coronary disease and prior myocardial infarction (MI), oral multivitamins and multiminerals (OMVMs), in a factorial design with edetate disodium (EDTA) chelation therapy, did not reduce cardiovascular events relative to placebo OMVMs, but active EDTA combined with active OMVMs was superior to placebo OMVM/placebo EDTA.

**Objective:**

To compare OMVM vs placebo in terms of efficacy for reducing major adverse cardiovascular events in patients with diabetes and prior MI.

**Design, Setting, and Participants:**

The TACT2 randomized, multicenter double-masked 2 × 2 factorial clinical trial took place across 88 sites in the US and Canada. Participants were 50 years or older, had diabetes, and had an MI 6 weeks ago or more. TACT2 participants were enrolled between September 2016 and December 2020. Data were collected between October 2016 and June 2023.

**Interventions:**

Six caplets daily of a 28 component OMVM or matching OMVM placebo, and 40 weekly infusions of an EDTA-based chelation solution or matching placebo, in a 1:1:1:1 allocation ratio.

**Main Outcomes and Measures:**

The primary end point was the composite of all-cause mortality, MI, stroke, coronary revascularization, or hospitalization for unstable angina.

**Results:**

A total of 1000 participants were randomized (500 in the active OMVM group and 500 in the placebo group). The median (IQR) age was 67 (60-72) years, and 730 (73%) were male. Median (IQR) follow-up was 48 (34-58) months. The primary end point occurred in 175 participants (35%) in the active OMVM group and 175 (35%) in the placebo group (hazard ratio [HR], 0.99 [95% CI, 0.80-1.22]; *P* = .92). The 5-year event rate for the primary end point in the EDTA chelation + active OMVM group was 34.0%; in the EDTA chelation + placebo OMVM group, 35.7%; in the placebo infusion + active OMVM group, 36.0%; and in the placebo infusion + placebo OMVM group, 34.3%. The comparison of the active infusion + active OMVM with the placebo infusion + placebo OMVM was not significant (HR, 0.91 [95% CI, 0.67-1.23]; *P* = .54). Although nonsignificant, there was a numerically higher event rate of MI, stroke, mortality from cardiovascular causes in the active OMVM compared to placebo OMVM group.

**Conclusions and Relevance:**

The results of this randomized clinical trial demonstrated that, for participants with chronic coronary disease, diabetes, and a previous MI, high-dose OMVM alone or in conjunction with EDTA-based chelation did not reduce cardiovascular events.

**Trial Registration:**

ClinicalTrials.gov Identifier: NCT02733185

## Key Points

**Question:**

In participants with diabetes and prior myocardial infarction (MI), do oral multivitamins and multiminerals (OMVMs), with or without edetate disodium (EDTA)-based chelation, reduce major adverse cardiovascular events compared to placebo OMVM?

**Findings:**

In this randomized clinical trial of 1000 participants with diabetes and prior MI, the findings of the initial Trial to Assess Chelation Therapy were confirmed in this patient population. OMVM did not reduce major adverse cardiovascular events compared to placebo OMVM, with or without with active EDTA-based chelation.

**Meaning:**

In participants with diabetes and prior MI, high-dose OMVMs alone or in conjunction with EDTA-based chelation did not reduce cardiovascular events.

## Teaser

This randomized clinical trial assesses the efficacy of oral multivitamins and multiminerals in reducing cardiovascular events in patients with diabetes after a myocardial infarction.

## Introduction

Between 2003 and 2010, the Trial to Assess Chelation Therapy (TACT) randomized 1708 participants with chronic coronary disease and a history of myocardial infarction (MI) to high doses of oral multivitamins and multiminerals (OMVMs) or placebo and edetate disodium (EDTA)–based chelation or placebo.^1^ The primary aim of the trial was to rigorously test the clinical effectiveness of EDTA chelation, which had been used clinically for decades for cardiovascular prevention on the basis of anecdotal evidence alone and despite the negative results of several small, randomized clinical trials. The lack of definitive evidence regarding clinical effectiveness was considered a public health problem by the National Institutes of Health (NIH), which provided funding for the trial. OMVMs were included as part of the trial intervention because of the prevailing belief that they provided adjunctive benefits to chelation. A 2 × 2 factorial design was chosen so that the effects of EDTA and OMVM could be examined both separately and in combination. In 2013, TACT reported an 18% reduction in the primary composite end point with EDTA chelation infusions vs placebo infusions (hazard ratio [HR], 0.82 [95% CI, 0.69-0.99]) but found that active OMVM did not reduce cardiovascular end points compared with placebo OMVM (HR, 0.89 [95% CI, 0.75-1.07]).^2^ In the factorial analyses, however, EDTA plus active OMVM was superior to placebo EDTA/placebo OMVM (HR, 0.74 [95% CI, 0.57-0.95]).^3^

Because TACT demonstrated the greatest risk reduction from EDTA in the subset of patients with diabetes, TACT2 studied patients with diabetes as well as a prior MI. TACT2 was designed to replicate TACT in diabetes, with the same EDTA and OMVM interventions and factorial design.^4^ We have previously published the results of the EDTA vs placebo comparison, which did not demonstrate any significant benefit for major adverse cardiovascular events.^5^ This report describes the OMVM vs placebo comparison as well as the factorial comparisons.

## Methods

### Study Design and Participants

TACT2 was a randomized, multicenter double-masked 2 × 2 factorial clinical trial, testing the effect of up to 40 weekly infusions of an edetate disodium-based regimen compared with placebo infusions, and high doses of OMVM compared with oral placebo. The full design of the study has been previously published.^4^ See the final trial protocol in Supplement 1.

Central and local institutional review boards at enrolling sites maintained study oversight. All participants provided written informed consent. The Data and Safety Monitoring Board (DSMB) members were selected by the NIH. The DSMB had the responsibility to review the accruing trial outcomes periodically and to recommend continuation or termination of the study to the NIH. The Consolidated Standards of Reporting Trials (CONSORT) reporting guideline was followed.

The 4 factorial groups included the following:

1. Active OMVM + active intravenous (IV) chelation infusions;
2. Placebo OMVM + active IV chelation infusions;
3. Active OMVM + placebo IV infusions; and
4. Placebo OMVM + placebo IV infusions.

The present study focuses on the active OMVM vs placebo OMVM comparison, and on the 4 factorial groups, to determine if there is an effect of OMVM on cardiovascular events, or an additive benefit with chelation, as was found in the original TACT trial. The infusion regimen lasted approximately 1 year. The OMVM or oral placebo intervention lasted up to 5 years. The results of the chelation vs placebo infusions comparison have been previously published.^5^

**Figure 1.**
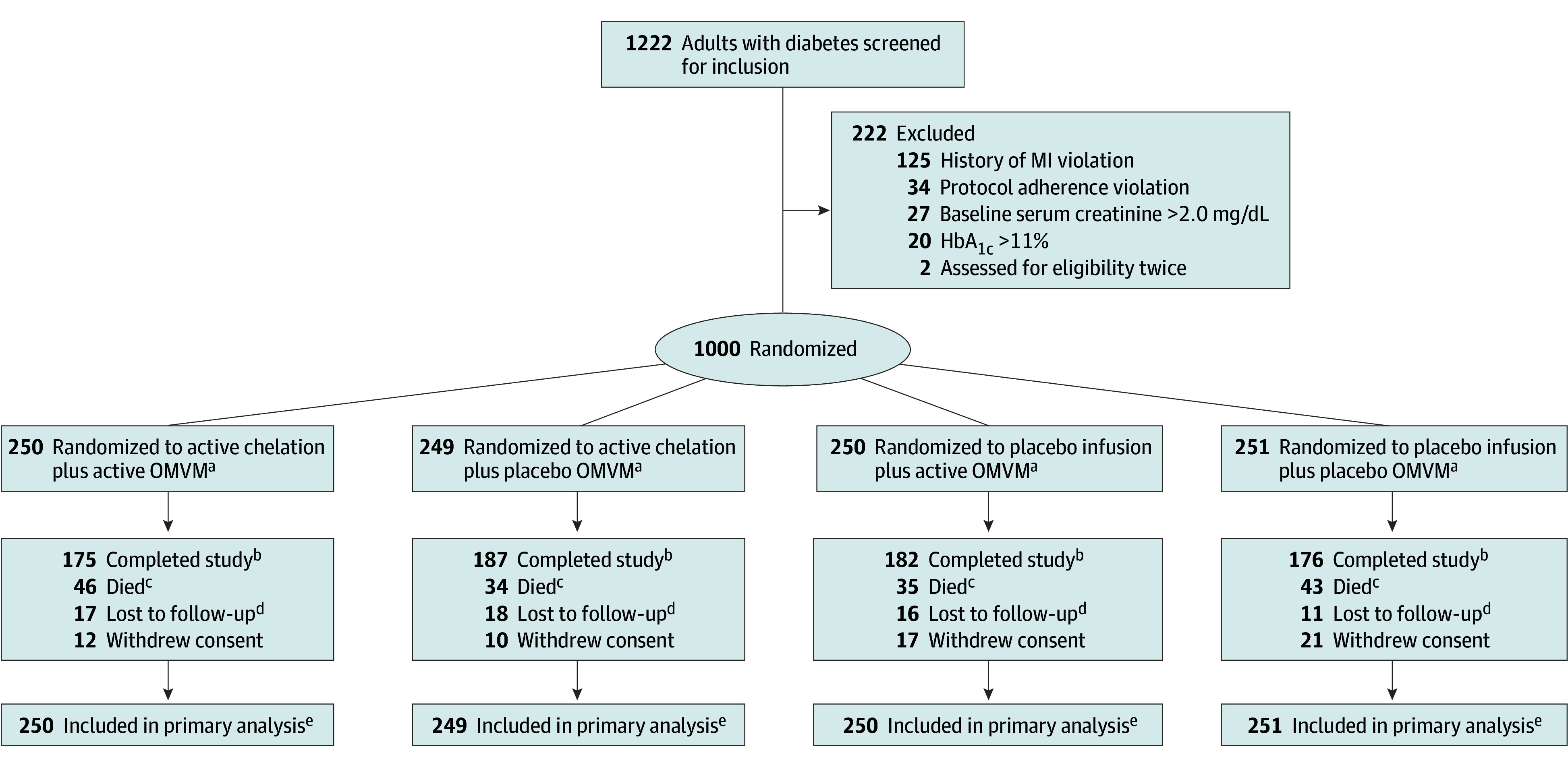
Screening, Randomization, and Follow-Up by 4 Treatment Groups SI conversion factors: to convert creatine to μmol/L, multiply value by 76.25; to convert hemoglobin A_1c_ (HbA_1c_) to proportion of total hemoglobin, multiply value by 0.01. MI indicates myocardial infarction. ^a^High-dose oral multivitamins and multiminerals (OMVM). ^b^Randomized participants who did not die, were not lost to follow-up, and did not withdraw consent are considered participants who completed the study. ^c^Participants who died before the end of study. ^d^Lost to follow-up is defined as no contact within 12 months of the end of study. End of study is defined as the 5-year informed consent expiration date or administrative end of study date (April 2024). Last contact is defined as the last known alive date. ^e^All randomized participants are included in the primary analysis population.

Participants were enrolled at 88 sites across the US and Canada between September 2016 and December 2020 (Figure 1). Eligible participants were 50 years and older and had an MI at least 6 weeks prior to enrollment. Participants were excluded if they were women of childbearing potential, had a serum creatinine level greater than 2.0 mg/dL (to convert to μmol/L, multiply by 76.25), platelet count less than 100 × 10^3^/μL (to convert to ×10^9^/L, multiply by 1), abnormal liver function studies, blood pressure greater than 160/100 mm Hg, past intolerance to any study component, greater than 1 dose of IV chelation therapy or other US Food and Drug Administration–approved chelation drug within 5 years, prior participation in the original TACT, coronary or carotid revascularization planned or having taken place within 6 months, cigarette smoking within 3 months, active heart failure or heart failure hospitalization within 6 months, or inability to tolerate 500-mL infusions weekly. Patient race and ethnicity were self-reported.

The clinical coordinating center at Mount Sinai Medical Center (Miami Beach, Florida) recruited sites and coordinated central pharmacy activities. The Duke Clinical Research Institute (DCRI; Durham, North Carolina) served as the data coordinating center and performed site and data management and statistical analyses. Biological samples for long-term storage and for metal analyses were processed at the trace metals and biorepository center at Columbia University Mailman School of Public Health (New York, New York). Each clinical site was led by a licensed physician. Site personnel obtained informed consent, evaluated, randomized, enrolled, and infused participants according to the randomized assignment. Sites collected and entered data into the trial electronic data collection system (Rave EDC [Medidata]). Follow-up took place at the sites during the infusion period and subsequently was transferred to the telephone-based DCRI participant research operations call center.

### Masking

The clinical sites, clinical coordinating center, and trace metals and biorepository center were fully masked to treatment assignment. A masked statistical team at the DCRI was responsible for all interactions with study staff. A separate unmasked statistical team at the DCRI received and analyzed unmasked data for presentation to the DSMB on a predetermined schedule. An independent unmasked team at NIH worked with the DSMB and the unmasked DCRI team.

### Treatments

#### OMVMs

The components and dose of active OMVM were identical to those used in TACT (September 2003-October 2011),^6^ and consisted of 3 caplets, taken twice daily, with 28 ingredients. See Table 1 for contents and comparison with recommended daily allowances. Placebo caplets contained microcrystalline cellulose, stearic acid, croscarmellose sodium, silica, and magnesium stearate. Active and placebo caplets were coated with OPADRY II (Colorcon) complete film white coating, magnesium stearate, and vanilla flavor. The OMVM or placebo caplets were mailed directly to the participants’ homes, to be taken for the duration of the study.

**Table 1.** Vitamin Content and Recommended Dietary Allowances (RDAs)^a^

High dose regimen (taken twice daily)	Total amount for 6 pills	RDA, %
Vitamin A	25 000 IU	833
Vitamin C	1200 mg	1333
Vitamin D_3_	100 IU	13
Vitamin E	400 IU	1818
Vitamin K_1_	60 µg	50
Thiamin	100 mg	8333
Niacin	200 mg	1250
Vitamin B_6_	50 mg	2941
Folate	800 µg	200
Vitamin B_12_	100 µg	4167
Biotin	300 µg	1000
Pantothenic acid	400 mg	8000
Calcium	500 mg	42
Iodine	150 µg	100
Magnesium	500 mg	125
Zinc	20 mg	182
Selenium	200 µg	364
Copper	2 mg	222
Manganese	20 mg	870
Chromium	200 µg	571
Molybdenum	150 µg	333
Potassium	99 mg	3
Choline	150 mg	NE
Inositol	50 mg	NE
PABA	50 mg	NE
Boron	2 mg	NE
Vanadium	35 µg	NE
Citrus Bioflavonoids	100 mg	NE

Abbreviations: NE, not established; PABA, para-aminobenzoic acid.

^a^ Placebo caplets consisted of microcrystalline cellulose, stearic acid, croscarmellose sodium, silica, and magnesium stearate. OPADRY II complete film white coating, magnesium stearate, and vanilla flavor were used to coat both active and placebo pills.

#### Low-Dose Vitamins

To prevent chelation-related depletion of some essential nutrients, all participants received a low-dose regimen consisting of a single daily pill to be taken when infusions were being performed. The low-dose regimen consisted of: vitamin B_6_, 25 mg; zinc, 25 mg; copper, 2 mg; manganese, 15 mg; and chromium, 50 μg.

#### EDTA Infusions

The contents of the infusions and masking strategies used have been previously published.^4^ The active infusions consisted of disodium EDTA, up to 3 g, adjusted based on estimated creatinine clearance; ascorbic acid, 7 g; magnesium chloride, 2 g; other additives; and sterile water, 500 mL. The placebo infusion appeared identical, consisting of normal saline, 500 mL, and dextrose, 1.2%, 2.5 g total. Infusions were administered weekly via IV access for at least 3 hours. A total of 40 weekly infusions were planned. There was flexibility for longer intervals between infusions due to sickness, vacations, and clinic site closures during the COVID-19 pandemic.

### Follow-Up, Safety Monitoring, and Adherence Assessment

During the planned infusion period, participants were seen at study sites for infusions and OMVM adherence was assessed during follow-up phone calls. The last follow-up contact was completed in June 2023. Monitoring during the infusion period included limited physical examinations and blood draws during the screening visit, and monitoring for potential adverse effects and hemoglobin A_1c_ levels at infusions 7, 15, and 40. The DCRI call center contacted participants 6 months and 12 months after randomization and then every 4 months until 5 years or the end of the study to determine the occurrence of clinical events and adherence to oral OMVM or placebo caplets. Adherence was assessed on each telephone visit by asking if participants were taking all, some, or none of the OMVM/placebo caplets.

### End Points

The TACT2 primary end point was a composite of death from any cause, MI, stroke, coronary revascularization, or hospitalization for unstable angina. A clinical events committee masked to treatment assignment adjudicated all nonprocedural components of the primary and secondary end points. Coronary revascularizations were verified from the source medical record by the DCRI. The 3 secondary end points were recurrent events of the primary composite end point, all-cause mortality, and composite of cardiovascular mortality, MI, or stroke. Metals were measured in blood and urine at infusions 1, 5, 20, and 40, using inductively coupled plasma mass spectrometry at the Centers for Disease Control and Prevention National Center for Environmental Health.^7^ Blood lead and urine cadmium were considered the primary metals of interest. Levels of other metals were also measured.

### Statistical Analysis

The sample size was based on the anticipated event rate with placebo and the effect size of the chelation vs placebo infusions. Thus, no formal power calculations for the OMVM comparison or the factorial analyses were used for planning the study. A total of 282 primary end point events was estimated to provide 85% power assuming a HR of 0.70 for chelation vs placebo infusion. TACT2 originally planned to enroll 1200 participants with a minimum follow-up of 12 months after the final infusion. Ultimately, and partially due to the COVID-19 pandemic, the final study enrollment was 1000 participants, and planned follow-up was extended to a minimum of 2.5 years for all participants, preserving statistical power for the chelation vs placebo infusion comparison.

A full description of the statistical analysis plans has been previously published.^4^ Continuous variables are presented as median and IQR, along with means and SDs, as appropriate. Categorical variables are summarized as frequencies and percentages. The primary statistical comparison is based on the time from randomization to the first occurrence of any of the primary composite event components using the Cox proportional hazards regression model. The Cox proportional hazards model included indicator variables for the active OMVM and active chelation groups, and adjustments for age, sex, and insulin use. For the analysis of the 2 × 2 factorial design, the reference group in the statistical models was the placebo infusion and placebo OMVM group. The remaining 3 treatment groups were compared with the reference group in the statistical models. For analyses of metal levels, we evaluated preinfusion urine and blood metal levels at infusions 1, 5, 20, and 40, comparing participants with active OMVM vs placebo OMVM to confirm that OMVM treatment did not have an effect on metal burden.

Prespecified subgroups included women and racial and ethnic minority groups, persons older than 70 years, particularly participants at high risk based on history of prior anterior MI, peripheral artery disease, pharmacologic treatment of diabetes, and participants not taking statins. All statistical analyses were performed using SAS statistical software, version 9.4 or higher (SAS Institute Inc). Statistical testing was 2-sided and significant at a threshold of *P* < .05.

## Results

### Baseline Characteristics

A total of 1000 participants were randomized. The median (IQR) follow-up was 48 (34-58) months. The baseline characteristics between active and placebo OMVM randomized groups with 500 participants each were well balanced (Table 2). The median (IQR) age was 67 (60-72) years, and 730 (73%) were male. The qualifying MI occurred at a median (IQR) of 5 (2-10) years before enrollment. The time from diagnosis of diabetes to randomization was a median (IQR) of 14 (7-21) years. A total of 960 participants (96%) had type 2 diabetes, with 468 of 997 (47%) requiring insulin, 153 (15%) taking sodium-glucose cotransporter 2 inhibitors, and 102 (10%) taking L agonists at baseline. Participants had a median (IQR) baseline hemoglobin A_1c _of 7.3% (6.5%-8.3%) (to convert to proportion of total hemoglobin, multiply by 0.01), and median (IQR) low-density lipoprotein cholesterol of 73 (56-96) mg/dL (to convert to mmol/L, multiply by 0.0259). Baseline characteristics by factorial groups are shown in eTable 1 in Supplement 2.

**Table 2.** Demographics and Baseline Clinical Characteristics in the Primary Analysis Population

Characteristic	No./total No. (%)	
	Active OMVM (n = 500)	Placebo OMVM (n = 500)
Demographics		
Age, median (IQR), y	66 (60-72)	67 (61-72)
Sex		
Female	136/500 (27.2)	134/500 (26.8)
Male	364/500 (72.8)	366/500 (73.2)
Race^a^		
American Indian or Alaska Native	3/500 (0.6)	2/500 (0.4)
Asian	27/500 (5.4)	27/500 (5.4)
Black/African American	53/500 (10.6)	48/500 (9.6)
Multiracial	2/500 (0.4)	4/500 (0.8)
Native Hawaiian or other Pacific Islander	10/500 (2.0)	8/500 (1.6)
White	390/500 (78.0)	388/500 (77.6)
Other^b^	15/500 (3.0)	23/500 (4.6)
Ethnicity		
Hispanic or Latino	99/496 (20.0)	99/491 (20.2)
Not Hispanic or Latino	397/496 (80.0)	392/491 (79.8)
Medical history^c^		
Diabetes		
Type 1	26/500 (5.2)	14/500 (2.8)
Type 2	474/500 (94.8)	486/500 (97.2)
Time from diabetes diagnosis to randomization, median (IQR), y^d^	14 (7-23)	14 (7-21)
Time from qualifying MI to randomization, median (IQR), y	5 (2-11)	5 (2-10)
Hypertension requiring treatment	459/499 (92.0)	459 (91.8)
Hypercholesterolemia^e^	455/496 (91.7)	449/498 (90.2)
Any cardiac revascularization (CABG or PCI)	408/496 (82.3)	405/499 (81.2)
Complications of diabetes^f^	243/498 (48.8)	210/495 (42.4)
Anterior MI	151/500 (30.2)	159/500 (31.8)
Congestive heart failure	98/500 (19.6)	109/500 (21.8)
Peripheral vascular disease	76/490 (15.5)	84/490 (17.1)
Stroke	52/497 (10.5)	41/498 (8.2)
Cardiovascular medications		
Aspirin, warfarin, or P2Y12 inhibitor	448/498 (90.0)	449/500 (89.8)
Statin	419/499 (84.0)	441/500 (88.2)
β-Blocker	393/498 (78.9)	400/500 (80.0)
Angiotensin-converting enzyme inhibitor or angiotensin receptor blocker	313/498 (62.9)	323/500 (64.6)
PCSK9 inhibitor	24/496 (4.8)	5/500 (1.0)
Diabetes medications		
Insulin	239/500 (47.8)	229/500 (45.8)
Other oral agent	333/498 (66.9)	350/500 (70.0)
GLP-1 receptor agonist or SGLT-2 inhibitor	113/498 (22.7)	109/498 (21.9)
DPP-4 inhibitor	58/496 (11.7)	59/499 (11.8)
Other diabetes medication	37/491 (7.5)	31/496 (6.3)
No diabetes medication	23/497 (4.6)	30/500 (6.0)
Vitals		
BMI,^g^ median (IQR)	31.8 (28.1-36.8)	31.5 (28.4-36.1)
Systolic blood pressure, mean (SD), mm Hg	132.6 (17.62)	132.6 (17.35)
Diastolic blood pressure, mean (SD), mm Hg	74.4 (10.41)	73.8 (10.16)
Laboratory examination results, median (IQR)		
Fasting glucose, mg/dL	136.0 (113.0-170.0)	134.0 (110.0-175.0)
Hemoglobin A_1c_, % of total hemoglobin	7.3 (6.5-8.3)	7.2 (6.5-8.4)
Creatinine, mg/dL	1.1 (0.9-1.3)	1.0 (0.9-1.2)
Calculated eGFR, mL/min/1.73 m^2^	67.0 (53.7-82.8)	71.1 (54.6-84.6)
HDL cholesterol, mg/dL	42.0 (35.0-50.0)	41.0 (34.0-49.0)
LDL cholesterol, mg/dL	73.0 (56.0-98.0)	73.0 (55.0-95.5)
Total cholesterol, mg/dL	145.0 (124.0-178.0)	143.0 (122.0-172.0)
Triglycerides, mg/dL	144.0 (104.0-220.0)	143.0 (101.0-206.0)
Blood and urine metals, median (IQR)		
Lead (blood), μg/L	9.69 (6.49-14.15)	8.80 (6.20-13.64)
Cadmium (urine), μg/g^h^	0.29 (0.16-0.50)	0.31 (0.19-0.53)

Abbreviations: BMI, body mass index; CABG, coronary artery bypass graft; DPP-4, dipeptidyl peptidase-4; eGFR, estimated glomerular filtration rate; GLP-1, glucagon-like peptide 1; HDL, high-density lipoprotein; LDL, low-density lipoprotein; MI, myocardial infarction; OMVM, oral multivitamin and multimineral; PCI, percutaneous coronary intervention; SGLT-2, sodium-glucose cotransporter 2.

SI conversion factors: To convert glucose to mmol/L, multiply value by 0.0555; hemoglobin A_1c_ to proportion of total hemoglobin, multiply by 0.01; creatine to μmol/L, multiply by 76.25; eGFR to mL/s/m^2^, multiply by 0.0167; cholesterol to mmol/L, multiply by 0.0259; triglycerides to mmol/L, multiply by 0.0113.

^a^ Race collected by selecting all that apply as reported by the participant. If multiple races were selected, the participant was included in the multiracial category.

^b^ The other category was participant reported.

^c^ Medical history was participant reported.

^d^ Year of diabetes diagnosis was collected. Day and month were imputed as June 1 of the collected year.

^e^ Total cholesterol greater than 240 md/dL; LDL greater than 130 mg/dL.

^f^ Laser/injection eye treatment for diabetic retinopathy, diabetic neuropathy, amputation, or hypoglycemia with help needed for treatment.

^g^ BMI calculated as weight in kilograms divided by height in meters squared.

^h^ Baseline urinary metal levels (μg/g) corrected for urinary creatinine (μg of metal/g of creatinine).

### Primary and Secondary End Points for OMVM

**Figure 2.**
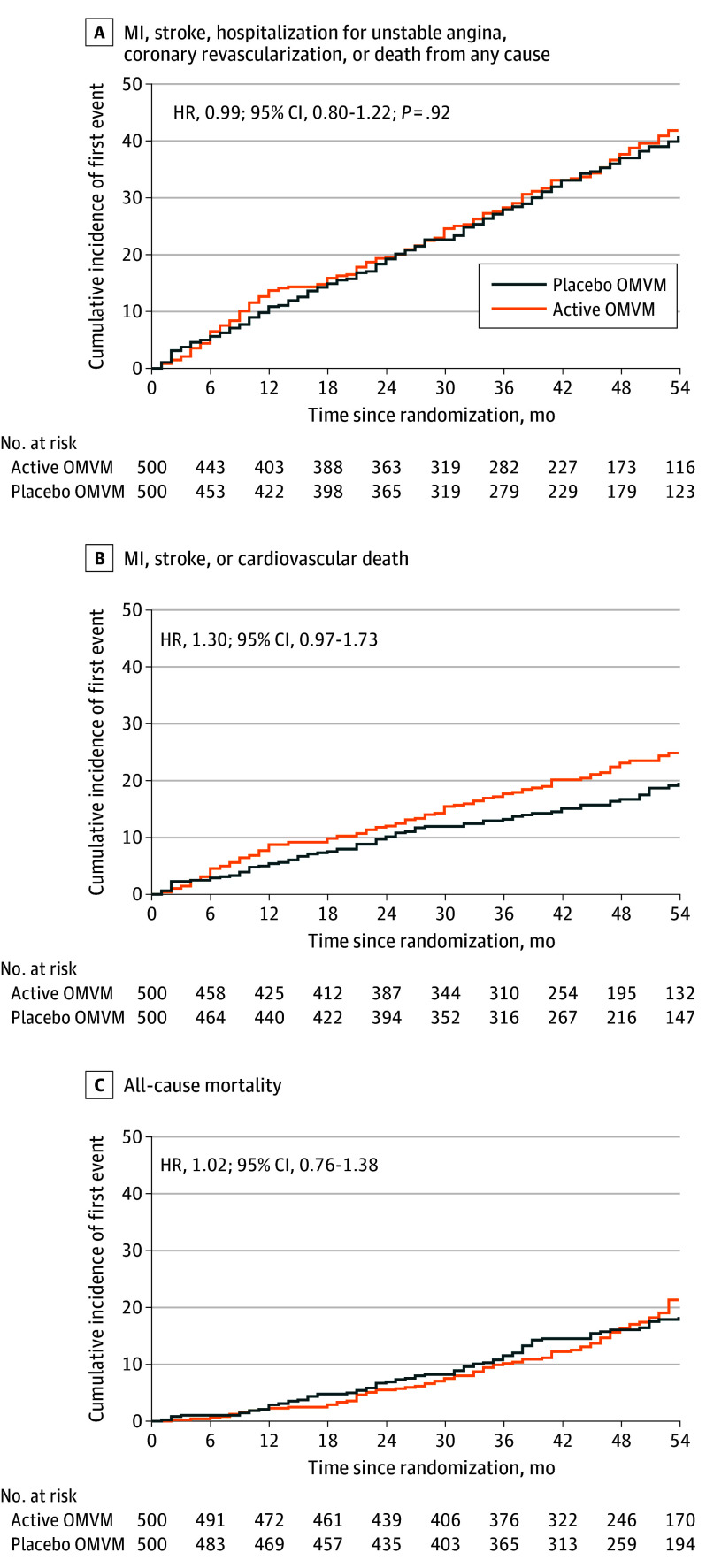
Cumulative Incidence of Time to First Event These values were derived from the primary analysis population. A, The primary outcome was a composite of myocardial infarction (MI), stroke, hospitalization for unstable angina, coronary revascularization, or death from any cause. B, A key secondary outcome was a composite of MI, stroke, or cardiovascular death. C, A key secondary end outcome was all-cause mortality. Beyond month 54, the sample size for the numbers at risk was lower than meaningful; therefore, the x-axis is displayed up to 54 months. The hazard ratios (HRs; active oral multivitamins and multiminerals [OMVM] group vs placebo OMVM group) and *P* value were derived from Cox proportional hazards regression with adjustment of edetate disodium chelation group, age, time-varying age, sex, and baseline insulin use. An HR of less than 1 indicates a benefit with active OMVM compared to placebo OMVM. Events occurring after withdrawal of consent or informed consent expiration were censored.

The primary end point occurred in 175 participants (35%) in the active OMVM group and 175 (35%) in the placebo OMVM group (HR, 0.99 [95% CI, 0.80-1.22]; *P* = .92; Figure 2A). There was no significant difference in cumulative incidence of first event of MI, stroke, or cardiovascular death between active OMVM and placebo OMVM (HR, 1.30 [95% CI, 0.97-1.73]; Figure 2B). This secondary composite outcome occurred in 105 participants (21%) in the active OMVM group and in 81 participants (16%) in the placebo OMVM group. There was no significant difference in all-cause mortality between groups (Figure 2C). There were no significant differences between treatment groups for the individual components of the primary end point (Table 3). There was no heterogeneity of effect of OMVM identified in any of the prespecified subgroups (eFigure 1A and 1B in Supplement 2).

**Table 3.** Active Oral Multivitamin and Multimineral (OMVM) vs Placebo OMVM: Primary and Key Secondary End Points

End point	Participants with event, No. (%)		% Difference, OMVM − placebo (95% CI)	Adjusted HR^a^ (95% CI)	*P* value^b^
	Active OMVM (n = 500)	Placebo OMVM (n = 500)			
Primary outcome					
Composite of MI, stroke, hospitalization for unstable angina, coronary revascularization, or death from any cause^c^^,^^d^	175 (35.0)	175 (35.0)	0 (−5.91 to 5.91)	0.99 (0.80 to 1.22)	.92
Components of primary composite outcome					
MI	54 (10.8)	48 (9.6)	NA	NA	NA
Stroke	20 (4.0)	10 (2.0)	NA	NA	NA
Hospitalization for unstable angina	17 (3.4)	20 (4.0)	NA	NA	NA
Coronary revascularization	32 (6.4)	42 (8.4)	NA	NA	NA
Death from any cause	52 (10.4)	55 (11.0)	NA	NA	NA
Secondary outcomes					
Composite of MI, stroke, or death from cardiovascular causes^c^^,^^e^	105 (21.0)	81 (16.2)	4.80 (−0.03 to 9.64)	1.30 (0.97 to 1.73)	NA
All-cause mortality^f^	90 (18.0)	85 (17.0)	1.00 (−3.73 to 5.73)	1.02 (0.76 to 1.38)	NA
Components of secondary composite outcome					
MI	58 (11.6)	51 (10.2)	NA	NA	NA
Stroke	22 (4.4)	11 (2.2)	NA	NA	NA
Death from cardiovascular causes	25 (5.0)	19 (3.8)	NA	NA	NA

Abbreviations: HR, hazard ratio; MI, myocardial infarction; NA, not applicable.

^a^ An HR of less than 1 indicates a benefit with active OMVM compared to placebo OMVM.

^b^ Type 1 error (2-sided α = .05) was used as the threshold for hypothesis testing. *P* values were only displayed if the prior end point was statistically significant.

^c^ Events occurring after withdrawal of consent or informed consent expiration were censored.

^d^ HR (active OMVM vs placebo OMVM) from Cox proportional hazards regression with adjustment of edetate disodium chelation group, age (and time-varying age), sex, and baseline insulin use. Participants with 2 or more events that occurred on the same day will show 1 event based on the following hierarchy: MI, stroke, hospitalization for unstable angina, coronary revascularization, and all-cause mortality.

^e^ HR (active OMVM vs placebo OMVM) from cause-specific Cox proportional hazards regression adjusted by edetate disodium chelation group, age (and time-varying age), sex, and baseline insulin use. Participants with 2 or more events that occurred on the same day will show 1 event based on the following hierarchy: MI, stroke, and cardiovascular mortality.

^f^ HR (active OMVM vs placebo OMVM) from Cox proportional hazards regression. Model was adjusted by edetate disodium chelation group, age, sex, and baseline insulin use. A death date after withdrawn consent or informed consent expiration (obtained from a public data source) was considered an event.

### Factorial Treatment Group Comparisons

The 5-year event rates for the primary end point were 85/250 (34.0%) in the EDTA chelation + active OMVM group; 89/249 (35.7%) in the EDTA chelation + placebo OMVM group; 90/ 250 (36.0%) in the placebo infusion + active OMVM group; and 86/251 (34.3%) in the placebo infusion + placebo OMVM group (eTable 2 in Supplement 2). The primary outcome with the EDTA chelation + active OMVM compared with the placebo infusion + placebo OMVM was not significantly different (HR, 0.91 [95% CI, 0.67-1.23]; *P* = .54). The secondary composite end point of time to first event for MI, stroke, or death from cardiovascular causes occurred in 50 participants (20.0%) in the EDTA chelation + active OMVM group, 40 (16.1%) in the EDTA chelation + placebo OMVM group, 55 (22.0%) in the placebo infusion + active OMVM group, and 41 (16.3%) in the placebo infusion + placebo OMVM group. The factorial results of cumulative incidence of time to first event of MI, stroke, hospitalization for unstable angina, coronary revascularization, or death for any cause can be found in eFigure 2 in Supplement 2. The treatment group comparisons for the secondary composite outcome and the all-cause mortality outcome were not significantly different. The Kaplan-Meier event rate estimates for the active EDTA chelation compared with placebo infusions have been previously published.^5^

### Treatment Adherence

OMVM adherence, defined as taking 1 or more tablets of the intervention at the specified visit among those with nonmissing data, was present in 457 participants (91.4%) in the active OMVM group vs 448 (89.6%) in the placebo OMVM group at infusion visit 40; and 303/395 participants (77%) in the active OMVM group vs 325/405 (80%) in the placebo OMVM group at 12-month postinfusion follow-up (eTable 3 in Supplement 2). There were 43 participants (9%) in the active OMVM group and 52 (10%) in the placebo OMVM group who did not take any vitamins (eFigure 3 in Supplement 2). The main reason for discontinuation was participant preference. Treatment adherence with the chelation/placebo infusion regimen has been previously published.^5^

### OMVM and Metal Levels

There was no significant difference in lead, selenium, and manganese levels over time in the preinfusion blood or urine samples when comparing participants on active OMVM vs placebo OMVM. Preinfusion urine molybdenum levels were higher at infusions 5, 20, and 40 vs infusion 1 when comparing participants on active OMVM vs placebo OMVM (eFigure 4A-4D and 5E-5G in Supplement 2). Preinfusion blood and urine lead declined over time in both OMVM treatment groups during the infusion period, mostly due to half of each group also receiving active chelation infusions. Preinfusion blood and urine cadmium levels remained stable during the infusion period. Preinfusion blood manganese levels increased over time, reflecting the effect of the low-dose vitamins, which included manganese, 15 mg. No significant difference was observed between the active OMVM and placebo OMVM group, despite the additional 20 mg of manganese in the active OMVM treatment. No significant differences were observed in blood selenium by OMVM treatment groups, although selenium, 200 μg, was included in the active OMVM treatment groups.

### Adverse Events

Serious adverse events were documented in 80 active OMVM recipients (16.8%) and 80 placebo OMVM recipients (16.6%) (between-group difference, −0.2% [95% CI, −5.0% to 4.5%]; eTable 4 in Supplement 2). There were 20 stroke events in the active OMVM group (4% of subgroup participants) compared to 10 in the placebo OMVM group (2% of subgroup participants) (Table 3). Among the adverse events, cardiac disorders were the most common, with 17 participants (3.6%) in the active OMVM group and 30 (6.2%) in the placebo OMVM group. There was no evidence suggesting harm from the EDTA-based infusion or oral vitamin therapy in any of the categories of adverse events.

## Discussion

The results of this randomized clinical trial demonstrated that, for participants with chronic coronary disease, diabetes, and a previous MI, the use of OMVM caplets containing 28 high-dose multivitamins and multiminerals was safe but did not reduce cardiovascular events during a 48-month median follow-up (IQR, 34-58) months. TACT2 enrolled secondary prevention patients at high risk for recurrent cardiovascular events based on their diabetes and prior history of MI. These results are consistent with the much larger body of trial-based evidence showing no benefit of multivitamins and multiminerals for primary prevention.^8,9,10^ The active OMVM group had a nominally higher proportion of strokes, MI, and death from cardiovascular causes compared to the placebo OMVM group, but the effect is best described as indeterminate due to insufficient precision. We were also unable to replicate the finding from TACT that the combination of active EDTA chelation and active OMVM significantly reduced the composite primary event rate relative to placebo EDTA/placebo OMVM.

Vitamins and minerals are involved in essential metabolic pathways, which are possible prime targets for improving or maintaining cardiovascular health, through mechanisms such as improvement of endothelial function, or reduction of oxidative stress.^11,12,13,14^ Yet clinical trials, focusing principally on primary prevention, have consistently demonstrated no benefit in reducing cardiovascular events.^10,15^^,^ In 2021, a meta-analysis including 3 multivitamin primary prevention studies did not reveal any benefit with supplementation of OMVM on total cardiovascular events or all-cause mortality.^16^ Finally, in 2024, a large cohort study of 390 124 participants demonstrated no benefit in mortality associated with the use of multivitamins.^15^ Based on such studies, the US Preventive Services Task Force has consistently recommended against the use of OMVM to improve cardiovascular health.^17^

While the OMVM used in TACT2 included selenium and manganese, we did not observe a difference in the corresponding blood or urine levels over time between the active OMVM group compared with the placebo group, which could be related to tight regulation of these essential elements. There was a difference in urine molybdenum levels over time between active and placebo OMVM, which is consistent with the 150 μg of molybdenum in OMVM supplementation. OMVM active treatment had no impact on the blood and urine levels of blood lead and cadmium.

### Limitations

Several caveats should be considered in the interpretation of these results. First, this study was conducted during the COVID-19 pandemic, which may have altered individuals’ dietary consumption and lifestyle, although active and placebo groups would have been affected equally. Second, OMVM adherence was imperfect, although similar discontinuation of the active OMVM and placebo OMVM was noted in both groups; adherence was similarly imperfect in the first TACT study. Third, the first TACT trial found an 11% relative benefit for the primary end point with a 95% CI that included the null. TACT2 was not sufficiently large to detect (or rule out) an effect size of this magnitude with precision. Finally, the absence of a detectable difference in some minerals in active OMVM vs placebo OMVM, despite those minerals being present in the active OMVM, raises questions about OMVM absorption.

## Conclusions

The results of this randomized clinical trial demonstrated that, for participants with chronic coronary disease, diabetes, and a previous MI, the use of high-dose OMVMs alone or in conjunction with EDTA-based chelation did not reduce cardiovascular events.

## Notes

Supplement 1. Trial Protocol

Supplement 2. eTable 1. Demographics and Baseline Clinical Characteristics for the 4 Treatment Groups (in the Primary Analysis Population) eFigure 1. A, Active OMVM vs Placebo OMVM: Subgroup Analysis of the Primary Outcome (in the Primary Analysis Population); B, Active OMVM vs Placebo OMVM: Subgroup Analysis of the Primary Outcome: Statins (in the Primary Analysis Population) eTable 2. 4 Treatment Group Analysis: Primary and Key Secondary End Points (in the Primary Analysis Population) eFigure 2. Cumulative Incidence of Time to First Event: Myocardial Infarction, Stroke, Hospitalization for Unstable Angina, Coronary Revascularization, or Death from Any Cause by 4 Treatment Groups (in the Primary Analysis Population) eTable 3. Participant OMVM Intervention Exposure Over Time (in the Primary Analysis Population) eFigure 3. Screening, Randomization, and Follow-Up (Screening Population) eFigure 4. Preinfusion Metal Levels at Baseline and Infusion Period for Urine Lead (µg of metal/g of creatinine), Blood Lead (µg/L), Urine Cadmium (µg of metal/g of creatinine), Blood Cadmium (µg/L), in the Primary Analysis Population eFigure 5. Preinfusion Metal Levels at Baseline and Infusion Period for Urine Molybdenum (µg of metal/g of creatinine), Blood Selenium (µg/L), and Blood Manganese (µg/L) (in the Primary Analysis Population) eTable 4. Overview of Serious Adverse Nonoutcome Events (Among Randomized Participants with One or More Infusion)

Supplement 3. TACT2 Nonauthor Collaborators

Supplement 4. Data Sharing Statement
