# Supplementary material for: Multivitamins After Myocardial Infarction in Patients With Diabetes: A Randomized Clinical Trial
Source: JAMA Intern Med. 2025 Mar 3;185(5):540–8. doi: 10.1001/jamainternmed.2024.8408 (PMC11877407; doi:10.1001/jamainternmed.2024.8408)
Supplement: Supplement 1. — Trial Protocol [file jamainternmed-e248408-s001.pdf]

TACT2

TRIAL TO ASSESS CHELATION THERAPY

A randomized, double-blind, controlled factorial clinical trial of edetate disodium-based chelation and high-dose oral vitamins and minerals to prevent recurrent cardiac events in diabetic patients with a prior myocardial infarction.

Principal Investigators:

Gervasio A. Lamas, MD, PI CCC
Mount Sinai Medical Center and Columbia University Medical Center

Kevin J. Anstrom, PhD, Co-PI DCC and
Daniel B. Mark, MD, MPH, Co-PI DCC
Duke Clinical Research Institute

Scientific PI for toxic metals analysis:
Ana Navas-Acien MD PhD MPH
Columbia University Mailman School of Public Health

Supported by:

National Institutes of Health

Study Intervention Provided by:

Edetate disodium is manufactured by Akzo Nobel Pharmaceuticals, Netherlands

Sponsor of IND:

Gervasio A. Lamas, MD, PI CCC
IND 66,743

REVISION HISTORY

February 8, 2016 (Original Version Date)
July 1, 2016
May 18, 2017
November 1, 2019
May 6, 2021

TABLE OF CONTENTS

PRÉCIS ... 4
1. STUDY OBJECTIVES ... 6
  1.1 Primary Objective ... 6
  1.2 Secondary Objectives ... 6
2. BACKGROUND AND RATIONALE ... 7
  2.1 Background on Condition, Disease, or Other Primary Study Focus ... 7
  2.2 Study Rationale ... 9
3. STUDY DESIGN ... 12
4. SELECTION AND ENROLLMENT OF PARTICIPANTS ... 14
  4.1 Inclusion and Exclusion Criteria ... 14
  4.2 Study Enrollment Procedures ... 15
5. STUDY INTERVENTIONS ... 16
  5.1 Interventions, Administration, and Duration ... 16
  5.2 Handling of Study Interventions ... 18
  5.3 Concomitant Interventions ... 20
    5.3.1 Optimal Concomitant Interventions ... 20
    5.3.2 Prohibited Interventions Following randomization ... 21
  5.4 Adherence Assessment ... 21
  6.2 Description of Evaluations ... 23
    6.2.1 Screening Evaluation ... 23
    6.2.3 Subsequent Infusion Visits (2-40) ... 24
    6.2.4 Blinding ... 25
    6.2.5 Completion/Final Evaluation ... 26
7. SAFETY ASSESSMENTS ... 26
  7.1 Specification of Safety Parameters ... 26
  7.2 Methods and Timing for Assessing, Recording, and Analyzing Safety Parameters ... 27
  7.3 Adverse Events and Serious Adverse Events ... 28
  7.4 Reporting Procedures ... 30
  7.5 Follow-up for Serious Adverse Events ... 32
  7.6 Safety Monitoring ... 32
8. INTERVENTION INTERRUPTION ... 33
9. STATISTICAL CONSIDERATIONS ... 33
  9.1 General Design Issues ... 33
  9.2 Sample Size and Randomization ... 33
  9.3 Definition of ITT Population ... 35
  9.4 Interim Analyses and Stopping Rules ... 35
  9.5 Outcomes ... 36
    9.5.1 Primary Outcome ... 36
    9.5.2 Secondary Outcomes ... 36
  9.6 Data Analyses ... 36
10. DATA COLLECTION AND QUALITY ASSURANCE ... 39
  10.1 Data Collection Forms ... 39
  10.2 Data Management ... 39

10.3 Quality Assurance 40
10.3.1 Training 41
10.3.2 Quality Control 41
10.3.3 Metrics 41
10.3.4 Protocol Deviations 41
10.3.5 Monitoring 41
10.4 Medical Information Coding 42
11. PARTICIPANT RIGHTS AND CONFIDENTIALITY 42
11.1 Institutional Review Board (IRB) Review 42
11.2 Informed Consent Forms 42
11.3 Participant Confidentiality 42
11.4 Study Discontinuation 43
12. COMMITTEES 43
13. PUBLICATION OF RESEARCH FINDINGS 44
14. REFERENCES 44

PRÉCIS

Study Title

Trial to Assess Chelation Therapy 2 (TACT2).

A randomized, double-blind, controlled factorial clinical trial of edetate disodium-based chelation and high-dose oral vitamins and minerals to prevent recurrent cardiac events in diabetic patients with a prior myocardial infarction (MI).

Objectives

TACT2 is being carried out to replicate TACT, which found a reduction of recurrent cardiovascular events in post-MI diabetic patients receiving edetate disodium-based chelation therapy.

The primary objective of TACT2, therefore, is to determine if the chelation-based strategy increases the time to the first occurrence of any of the components of the TACT2 primary endpoint: all-cause mortality, myocardial infarction, stroke, coronary revascularization, or hospitalization for unstable angina compared to the placebo chelation strategy.

The secondary objectives of TACT2 are to determine:

1. if the chelation-based strategy reduces the overall rate of occurrence of the events which define the primary TACT2 endpoint: all-cause mortality, myocardial infarction, stroke, coronary revascularization, or hospitalization for unstable angina events compared to placebo chelation strategy.
2. if the chelation-based strategy increases the time to the first occurrence of a composite endpoint: cardiovascular mortality, recurrent myocardial infarction, or stroke compared to placebo chelation strategy.
3. if the chelation-based strategy increases the time to all-cause mortality compared to placebo chelation strategy.

Design and Outcomes

TACT2 is a 2x2 factorial trial testing 40 weekly edetate disodium-based chelation infusions and twice-daily high-dose oral multivitamins and multiminerals (OMVM) in a placebo-controlled design.

The primary endpoint is a composite of all-cause mortality, myocardial infarction, stroke, coronary revascularization, or hospitalization for unstable angina**.** All randomized patients will be followed until the end of the trial.

The secondary endpoints for TACT2 include the following:

Total number of CV events: This endpoint will include all CV events from randomization to the end of the study.

All-cause mortality: All-cause mortality is a secondary endpoint for the trial, based upon its importance and the observed 43% relative risk mortality reduction by chelation alone (P=0.011) in the diabetic subgroup of TACT.

Composite of cardiovascular mortality, recurrent myocardial infarction, or stroke: This composite secondary endpoint captures serious, irreversible, ischemic events. Chelation treatment led to a 40% relative reduction in risk (P=0.017) in this endpoint in the diabetic subgroup of TACT.

Exploratory analyses, defined as those seeking to confirm a signal of benefit observed in TACT, or to develop mechanistic hypotheses for benefits of chelation expected in TACT2, include:

i. Analysis of the primary endpoint in patients receiving OMVM compared with oral placebo
ii. Analysis of the primary endpoint in patients receiving active chelation + active OMVM, compared with placebo chelation + placebo OMVM
iii. Analysis of markers of severity of diabetes (fasting glucose, HbA1c, microalbuminuria) throughout the infusion regimen
iv. Analysis of lead and cadmium levels at multiple times during the infusion regimen
v. Collection of biospecimens for the constitution of a biorepository to permit future analyses yet to be conceived

Sample Size and Population

TACT2 has enrolled 1000 diabetic patients 50 years of age or older with a prior MI and a serum creatinine of 2.0 mg/dL or less. Patients will be randomly allocated (1:1:1:1) to 4 factorial groups, each with approximately 250 patients. TACT2 will use a simple randomization scheme. The four treatment groups are:

1. Active chelation + active OMVM
2. Active chelation + placebo OMVM
3. Placebo chelation + active OMVM
4. Placebo chelation + placebo OMVM

The study is targeting a population consisting of 40% women and 18% minority subjects.

1. STUDY OBJECTIVES

TACT2 is being carried out to replicate TACT, which found a reduction of recurrent cardiovascular events in post-MI diabetic patients receiving edetate disodium-based chelation therapy.

1.1 Primary Objective

The primary objective of TACT2, therefore, is to determine if the chelation-based strategy increases the time to the first occurrence of any of the components of the TACT2 primary endpoint: all-cause mortality, myocardial infarction, stroke, coronary revascularization, or hospitalization for unstable angina compared to the placebo chelation strategy.

1.2 Secondary Objectives

The secondary objectives of TACT2 are to determine:

1. if the chelation-based strategy reduces the overall rate of occurrence of the events which define the primary TACT2 endpoint: all-cause mortality, myocardial infarction, stroke, coronary revascularization, or hospitalization for unstable angina events compared to placebo chelation strategy.
2. if the chelation-based strategy increases the time to the first occurrence of a combined secondary endpoint: cardiovascular mortality, myocardial infarction, or stroke compared to placebo chelation strategy.
3. if the chelation-based strategy increases the time to all-cause mortality compared to placebo chelation strategy.

Exploratory analyses, defined as those seeking to confirm a signal of benefit observed in TACT, or to develop mechanistic hypotheses for benefits of chelation expected in TACT2, include:

i. Analysis of the primary endpoint in patients receiving OMVM compared with oral placebo

ii. Analysis of the primary endpoint in patients receiving active chelation + active OMVM, compared with placebo chelation + placebo OMVM

iii. Analysis of markers of severity of diabetes (fasting glucose, HbA1c, microalbuminuria) throughout the infusion regimen

iv. Analyses of lead and cadmium in blood and urine to:

   a. Compare the change in metal internal dose (blood lead and urine cadmium) in TACT2 participants, from infusion 1 to infusion 40 (or 1 year) in the active versus placebo infusion arms.

   b. Evaluate whether higher screening metal internal dose (blood lead and urine cadmium) and the extent of depletion of those stores identify patients more likely to benefit from chelation.

v. Collection of biospecimens for the constitution of a biorepository to support future

mechanistic work. Samples of blood (plasma and buffy coat) and urine will be stored at -80ºC in a secured biorepository to ensure their availability for the evaluation of future hypotheses that will be guided by the results of the trial.

The above metal collections and biorepository will serve to support analyses to determine whether post-infusion urine metal measurements, alone and in comparison to the corresponding pre-infusion measurements, have clinical significance for predicting outcomes and clinical benefit of chelation; and whether the suite of additional metal levels measured in blood and urine by inductively coupled plasma mass multi-element analytic technique, some of which are not chelated by edetate disodium, have clinical (prognostic) significance in preliminary testing

2. BACKGROUND AND RATIONALE

2.1 Background on Condition, Disease, or Other Primary Study Focus

Chelation treatment was first reported to have clinical benefits in symptomatic CAD patients in 1956 and 1961.[1-2] In the early 1960s, however, following a reappraisal by Kitchell,[3] only alternative medicine practitioners continued to use chelation for prevention of complications of atherosclerosis based on their positive anecdotal experiences.[4-6] Ultimately, academic authors published 3 small studies[7-9] with an aggregate of <300 patients and concluded that there was no benefit from chelation for surrogate endpoints such as time to claudication or angina. Yet these studies were too small and of too short a duration to exclude a small or moderate effect on clinical endpoints.[10] Despite recommendations against the practice of chelation by numerous professional medical associations,[11] patients continued to seek, and some practitioners to administer, chelation therapy for a variety of medical conditions.[5]

The National Institutes of Health (NIH) funded TACT in 2002. The design was a 2X2 factorial, randomly allocating patients to intravenous edetate disodium-based chelation or placebo, and high-dose oral multivitamins and multiminerals or oral placebo. Standard chelation practice in the community also included the addition of oral supplements to the intravenous treatments; hence the factorial design.[12] There were 1708 post-MI patients enrolled in 134 sites and 55,222 infusions administered as part of TACT.

In guideline-treated post-MI patients, chelation alone led to an 18% reduction in a combined cardiovascular endpoint (HR 0.82 (0.69, 0.99) P=0.035, 7-year NNT=18).[13] Patients with diabetes had dramatic benefit from IV chelation (HR 0.59, (0.44, 0.79), P=0.0002, 5-year NNT=6.5).[14] An analysis of the factorial groups demonstrated that the strategy of intravenous chelation plus OMVM yielded a 26% reduction over double placebo (HR 0.74 (0.57, 0.95), P=0.016, 5-year NNT=12).[15] The effect size and the potential for an impact on public health and health care financing were the primary reasons for the Investigators' desire to move forward with TACT2, in this case focusing on the patients in whom the greatest benefit had been observed (Table 1). The investigators also felt that inclusion of an exploratory mechanistic component to the clinical trial would be desirable. TACT2, therefore, if positive, will be a confirmatory

second trial of edetate disodium-based chelation for patients with established coronary disease, and should provide the groundwork for mechanistic hypotheses – that ubiquitous pro-oxidant toxic metals, especially lead and cadmium, are associated with recurrent coronary events.[16-20]

Table 1. Summary of Results from TACT

Population*:	Endpoint**	Treatment comparison	Event rate relative reduction	p-value	5-year NNT
Overall	Primary	EDTA vs. placebo	18%	0.035	18
Overall	Primary	EDTA + OMVM vs. placebo + placebo	26%	0.016	12
Diabetes	Primary	EDTA vs. placebo	41%	0.0002	6.5
Diabetes	Death	EDTA vs. placebo	43%	0.011	12
Diabetes	Primary	EDTA + OMVM vs placebo + placebo	51%	<0.001	5.5

* Post-MI, age ≥ 50 years, creatinine ≤ 2.0; ** time-to-event. Primary endpoint: time to death, MI, stroke, coronary revascularization, and hospitalization for angina; MVM: Multivitamins & Multiminerals; NNT: number needed to treat to prevent an event.

The OMVM versus placebo comparison demonstrated an 11% relative reduction in risk that was not statistically significant.[15] Yet, as can be seen above, there appears to be a numerical, but not statistically significant, signal of benefit from OMVM, reducing the hazard ratio by about 10% each time OMVM is added to the chelation infusion. The TACT vitamins included higher doses of the individual OMVM components than previously used in other trials, and the non-significant signal of potential benefit challenges current opinions on vitamin use for cardiovascular disease. Thus, the TACT2 protocol replicates the 2x2 factorial design initially used in TACT.

One of the two factors in the 2x2 factorial design – OMVM – is not incorporated in a primary or a secondary objective of the trial, and appears only in exploratory analyses. The reasons for this are as follows:

1. A factorial design will allow us to discern whether there is replication of the effect of OMVM on clinical events.
2. If there is an effect, the complex chelation strategy with 38 components will likely be linked, to the benefit of intravenous chelation, and new lines of research into the vascular effects of ultra-high dose oral anti-oxidant mixtures given new life. This will apply even if the effect is numerical and qualitative, and not necessarily statistically significant. Observing the same signal of benefit in 2 studies would be very important.
3. The most likely result, given the weight of prior vitamin research, is that there will be no effect of OMVM. If this is true, then the 28 oral components can be unlinked from the chelation strategy. This will improve patient compliance and, for the cardiology community, reduce barriers to acceptance and implementation.
4. There will be no statistical cost (assuming additive treatment effects) to the chelation vs placebo infusion comparison, which in TACT (diabetes) had a HR of 0.59. The proposed sample *size will provide 85% power to detect a HR of 0.70 for the primary endpoint, and 95% power to detect a HR of 0.65, both conservative estimates based on TACT results. In summary, if vitamins are not an essential part of the effectiveness of the TACT regimen, being able to clarify*

this in TACT2 will allow FDA to consider the approval of chelation alone and will make the treatment significantly easier to administer clinically. At the same time, if the relationships in the factorial comparisons of TACT can be reproduced in TACT2 for both chelation and vitamins, we will have opened up two major new research areas rather than just one.

2.2 Study Rationale

Coronary heart disease (CHD) is the leading cause of premature morbidity and mortality in the United States, including in patients with diabetes. In 2012 there were 29.1 million diabetic patients in the US, including 27% of people age 65 or older.[21] Of these, 6.4 million had coronary artery disease (CAD) and could potentially be eligible for the therapy to be tested in this application. Despite medical research advances that have identified effective medical therapy to reduce coronary events in diabetes, 68% of the 223,000 diabetic patients who died in 2012 had heart disease as either the principal or contributing cause. Compared with non-Hispanic whites, the risk of diagnosed diabetes is 70% higher among African Americans and 80% higher among Hispanics.[21] Although we do not fully understand the mechanism of chelation benefit and how it possibly relates to risk of diabetic patients, the chelation strategy diabetic patients had their risk of major adverse cardiovascular events reduced to the level of those patients who did not have diabetes. Chelation, in other words, appeared to lower the risk of diabetes for cardiovascular events to the non-diabetic post-MI patient level. This finding alone has potentially major public health implications. From an economic perspective, diabetes is a costly disease that consumes a large proportion of the national health care budget. In the US, 1 in 5 health care dollars is spent treating people with diabetes. The annual expenditure exceeds $245 billion (B), including $176B in direct costs and $69B in indirect costs (disability, work loss, premature mortality).[22] TACT2, if positive, will likely have major public health and scientific impact.

Possible Benefit of Chelation Therapy

The epidemiologic evidence that many metals are toxic and promote cardiovascular disease is robust, particularly for lead and cadmium,[16-20, 23-24] metals chelated by the edetates.[25-26] One possible hypothesis, which is an exploratory analysis within the present trial, is that edetate disodium-based chelation depletes body stores of ubiquitous toxic metal pollutants, lead and cadmium in particular, acquired from the environment over many years and that depletion of toxicants leads to improved outcomes.

EDTA Pharmacology

The present trial requires intravenous administration of the test article, edetate disodium. This is supported by extensive literature demonstrating poor absorption from the gastrointestinal tract. Only 5-10% of an oral dose is absorbed in the body, 91% of an oral dose is recoverable from the feces, and 4% is recoverable from the urine.[27] In blood, the drug is found in the plasma compartment. EDTA does not appear to penetrate cells; it is distributed primarily in the extracellular fluid with only about 5% of the plasma concentration found in the spinal fluid. The half- life of EDTA is 20 to 60 minutes. EDTA is excreted primarily by the kidneys, with about 50% excreted in one

hour and over 95% within 24 hours.[27] Calcium and other chelates are excreted in the urine bound to the EDTA. Almost none of the compound is metabolized.

The biological effects of EDTA are due to the formation of chelates with divalent and trivalent metals. The stability of the metal-EDTA complex is directly related to its pH – the higher the pH, the more stable the chelate. Intravenous infusions of edetate disodium result in the chelation of ionized calcium.[28] Transient but mild reduction of serum calcium can be observed following the slow intravenous infusion of EDTA, and was observed in TACT (below). 1gm of EDTA can effectively bind approximately 120 mg of calcium. Among metals normally found as trace metals and metals present pathologically, EDTA has been demonstrated to bind and promote the excretion of cadmium, lead, zinc, copper, iron, manganese, and vanadium.[29-30] Virtually all of the metals chelated by EDTA are excreted in the urine within 24 hours.[26] In the case of calcium, 28% is excreted during the infusion, 60% in the 6 hours following the infusion, and the remainder between 6 and 12 hours after.[31] Among the essential metals chelated by disodium EDTA are copper and iron. Both of these metals have an important role in oxidative state and generally exist in an intracellular compartment or protein-bound. However, there is an important, non-protein bound component that may be chelated by EDTA salts. EDTA salts may promote the excretion of up to 10 mg/day of iron and increase urinary copper by almost 140%.[32] The oral supplement regimen in TACT2 repletes copper. Iron was not substantially diminished by EDTA in TACT, and will be measured during the TACT2 infusion regimen.

Preliminary data on metal excretion following a TACT2 infusion
A preliminary study measured metal excretion after one infusion of placebo, and one of active chelation. Twenty male participants (mean age 66, range 51 to 81) were recruited using the same criteria as in TACT (post-MI, age $\geq$50, creatinine ≤ 2.0 mg/dL). Urinary concentrations of 20 toxic metals (Al, Sb, As, Ba, Be, Bi, Cd, Cs, Gd, Hg, Pb, Ni, Pd, Pt, Te, Tl, Th, Sn, W, U) normalized to creatinine excretion were measured at baseline, for 6 hours following a placebo infusion of 500 mL normal saline and 1.2% dextrose, and for 6 hours following a 3 gram edetate disodium-based infusion as used in TACT.

Following an edetate disodium-based TACT infusion, there was a striking increase in the urinary excretion of lead (mean 3,926% increase, P<0.001; Figure A), cadmium (739% increase, P<0.001; Figure B), and nickel (131% increase, P<0.001). There was also a modest increase in the urinary excretion of tin and thallium (30-40%; P<0.05). These preliminary data[25] show that

Effect of Placebo or Edetate disodium-based infusion on urinary lead (A) and cadmium (B) excretion*

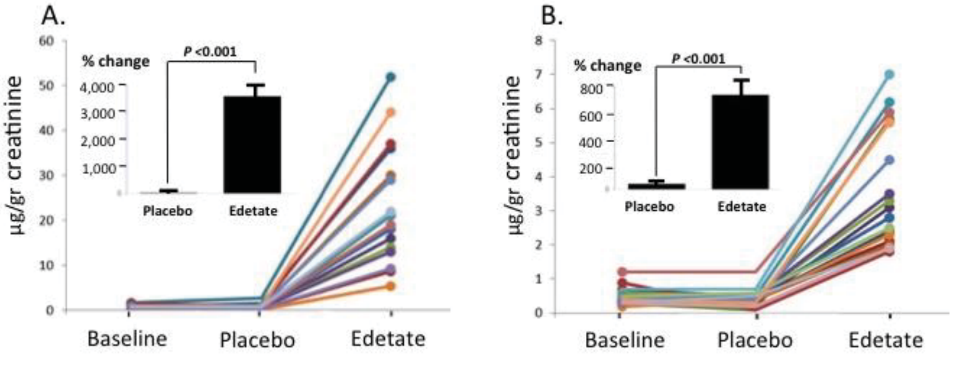

*Lines represent individual patient data points. Bar graphs are mean ± SD of % change from baseline.

an edetate disodium-based infusion, identical to that used in TACT and proposed for TACT2, markedly increased excretion of lead and cadmium. Whether enhanced excretion of metals following repeated chelation infusions leads to a reduction in blood lead or pre-infusion urine cadmium (reflecting total body stores of each) is unknown, but will be identified by TACT2.

FDA safety information and labeling on edetate disodium
Edetate disodium is an old drug for which no clear modern indication exists. Its current approval, as listed on the FDA website,[33] is for medical conditions for which more modern, better treatments exist:

> *"Edetate disodium is approved by the FDA for use in selected patients with high blood calcium levels (hypercalcemia) as well as for use among patients with heart rhythm problems due to intoxication with the drug, digitalis."* [33]

Edetate disodium has a long safety record, which was reviewed by FDA in 2007. Between 1971 and 2007, a time period during which hundreds of thousands of doses of edetate disodium were likely used, there were 11 deaths reported to FDA associated with the use of edetate disodium. According to FDA, 7 of 11 deaths resulted from confusion of edetate disodium with another drug, and hence its misadministration, either with too high a dose or more commonly as an IV push rather than a slow infusion. Thus, in a period spanning 36 years, there were 4 deaths associated with edetate disodium, in which edetate disodium was not misused by being confused with a different drug. This is consistent with the safety data for TACT presented below.

Safety of EDTA chelation as reported in TACT
We amassed a substantial body of safety data in TACT. EDTA was associated with few side effects and there were no differences in serious adverse events compared with placebo.[13,14,34] Some of the safety specifics are summarized below.

- **Calcium.** Hypocalcemia, defined as a calcium level less than 8.5 mg/dL prior to an infusion, was reported in 52 chelation patients (6.2%) and 30 placebo patients (3.5%) (P=0.008). One patient, non-diabetic, had symptomatic hypocalcemia associated with muscle cramping that led to an emergency department visit. With regards to bone health, there were 2 fractures in the patients receiving the full chelation strategy and 2 fractures in those receiving double placebo (P=1.00). Although no safety signal pertaining to bone health was found in TACT, the effect of chelation on calcium makes it appropriate to measure calcium during the infusion period, as well as designate fractures as an event of interest and specifically query for it at regular intervals in the clinic visits and the telephone follow-up.
- **Kidney.** An abnormal creatinine during the infusion phase was reported in fewer 3/839 (0.4%) placebo patients than chelation 9/868 (1%) patients (P=0.093). Because EDTA is renally excreted, creatinine will be followed during the infusion period. Vitamin C in high doses has been associated with oxalate kidney stones, and this was a rare complication in TACT.

- **Liver**. There were no significant differences in liver enzyme abnormalities in the chelation compared with the placebo group. Liver function will therefore not be monitored after the screening study.
- **Hematology**. There were no significant differences in abnormalities of hematologic parameters (white blood cells, hematocrit, and platelet count) in EDTA compared with placebo groups. Complete blood counts will be assessed at screening, infusion 20, and at the end of the infusion period or 1 year.
- **Hypoglycemia**. There was 1 episode of hypoglycemia reported in TACT, not clearly caused by the infusion. Because diabetic patients in TACT2 may possibly have hypoglycemic events due to the treatment of their diabetes, we have retained this potential adverse event in the ICF.
- **Death**. There were 2 deaths attributed by the blinded Medical Monitor as possibly or probably due to study therapy: 1 in the edetate disodium group and 1 in the placebo group. This potential adverse effect has been retained in the ICF.

Safety – chelation infusions in the population with diabetes: In the patients with diabetes, chelation was as safe as in the non-diabetic population. There were 95 serious adverse events (non–endpoint events) in the population with diabetes mellitus (56 chelation placebo and 39 chelation active).

Safety – Oral multivitamins and multiminerals: Serious adverse events occurred in 124 (15%) vitamin recipients and 103 (12%) placebo recipients (difference, 3 percentage points [95%CI=0.7 to 5.7 percentage points]). Adverse events included 12 (1.4%) incident neoplasms in the vitamin group and 11 (1.3%) in the placebo group (difference, 0.1 percentage point [95%CI= 0.8 to 1.3 percentage points]). No evidence suggested harm from vitamin therapy in any category of adverse events.

3. STUDY DESIGN

TACT2, like TACT, is a 2X2 double-blind, placebo-controlled multicenter international (US and Canada) factorial trial testing 40-weekly edetate disodium-based chelation infusions and twice daily OMVM. On December 31, 2020, TACT2 completed enrollment. The study enrolled 1000 diabetic patients 50 years of age or older with a prior MI and a serum creatinine of 2.0 mg/dL or less. The factorial design was selected for TACT2 because it replicates the TACT design and it provides the most information about the independent contributions of the chelation and OMVM factors. Patients will be randomly allocated (1:1:1:1) to 4 factorial groups:

1. Active chelation + active OMVM
2. Active chelation + placebo OMVM
3. Placebo chelation + active OMVM
4. Placebo chelation + placebo OMVM

Patients will be followed for clinical events until the beginning of the analysis phase, 3 months before the end of the 5-year trial. During the infusion period, patients will have frequent clinical and laboratory monitoring to determine whether a clinical endpoint has

been reached, to assess study safety, and to obtain biosamples for the exploratory analyses of metal excretion, and to establish the biorepository. Starting 6 months after randomization, patients will be followed by telephone from the DCRI Call Center. The combination of early clinic visits and samples, combined with later telephone follow-up will permit the ascertainment of clinical and laboratory goals in TACT2, as listed below and in greater detail in Section 6.1.

The primary objective of TACT2 is to determine if the chelation-based strategy increases the time to the first occurrence of any of the components of the TACT2 primary endpoint: all-cause mortality, myocardial infarction, stroke, coronary revascularization, or hospitalization for unstable angina compared to the placebo chelation strategy.

The secondary objectives of TACT2 are to determine:

1. if the chelation-based strategy reduces the overall rate of occurrence of the events which define the primary TACT2 endpoint: all-cause mortality, myocardial infarction, stroke, coronary revascularization, or hospitalization for unstable angina events compared to placebo chelation strategy.
2. to determine if the chelation-based strategy increases the time to the first occurrence of a combined secondary endpoint: cardiovascular mortality, recurrent myocardial infarction, or stroke compared to placebo chelation strategy.
3. to determine if the chelation-based strategy increases the time to all-cause mortality compared to placebo chelation strategy.

Additional analyses, defined as those seeking to confirm a signal of benefit observed in TACT, or to develop mechanistic hypotheses for benefits of chelation expected in TACT2, include:

i. Analysis of the primary endpoint in patients receiving OMVM compared with oral placebo to determine whether the signal of OMVM benefit is reproduced
ii. Analysis of the primary endpoint in patients receiving active chelation + active OMVM, compared with placebo chelation + placebo OMVM to determine if combination therapy has a greater effect size, as observed in TACT
iii. Analysis of markers of diabetes (fasting glucose, HbA1c, microalbuminuria) throughout the infusion regimen to determine whether changes in diabetes control or microvascular effects may be associated with the TACT2 results
iv. Analysis of lead and cadmium levels collected at multiple times during the infusion regimen to assess whether toxic metal removal is associated with the TACT2 results
v. Collection of a specimen biorepository to permit future analyses yet to be conceived

4. SELECTION AND ENROLLMENT OF PARTICIPANTS

4.1 Inclusion and Exclusion Criteria

All TACT2 participants must meet of the following inclusion criteria shown in Table 2. Potential TACT2 participants meeting any of the exclusion criteria in Table 2 will be excluded from study participation.

Table 2: Inclusion and exclusion criteria

INCLUSION CRITERIA (all must be present)	EXCLUSION CRITERIA (none may be present)
1. Age ≥ 50 years<br>2. History of diabetes, defined as medical record evidence or patient report of currently using insulin or oral hypoglycemic agents, or with a history of fasting blood glucose measurement of 126 mg/dL or higher, or a history of HbA1c of 6.5% or higher.<br>3. History of myocardial infarction based on the Universal Definition of MI.[35]<br>a. When information about the MI hospitalization is available, all MI types except Type 2 qualify for study entry.<br>b. When information about the MI hospitalization is not available, a wall motion abnormality on imaging or a perfusion defect on scan that corresponds to a coronary distribution, whether or not accompanied by pathological Q waves in the appropriate distribution, will qualify the patient for study entry. This criterion requires a call to the CCC for case review.	1. Baseline serum creatinine >2.0 mg/dL.<br>2. HbA_1C >11%.<br>3. Myocardial infarction within 6 weeks of randomization.<br>4. History of allergic reactions to EDTA or any other components of the chelation solution, including heparin. Site personnel are to call the CCC to discuss heparin allergy.<br>5. Coronary or peripheral arterial revascularization procedure performed within the last 6 months.<br>6. Planned revascularization procedure in the 6 months following enrollment.<br>7. Heart failure hospitalization within 6 months prior to enrollment or in clinical heart failure at the time of proposed enrollment (such as NYHA Class 3 dyspnea + rales >basilar, and additional signs of fluid overload). Such patients may be treated with diuretics and enrolled when stable.<br>8. Poor or no venous access in the upper extremities.<br>9. a. Prior intravenous chelation therapy consisting of > 1 infusion within 5 years; if only 1 infusion took place, patient cannot be enrolled for at least 12 months after said infusion.<br>b. Oral chelation therapy with an approved oral chelating agent within 2 years.<br>10. Prior participation in TACT.<br>11. Baseline platelet count <100,000.<br>12. History of cigarette smoking within the last 3 months.<br>13. ALT or AST > 2.0 times the upper limit of normal.<br>14. Wilson's disease, hemochromatosis, or parathyroid disease.<br>15. Any medical condition including a current diagnosis of cancer (except non-melanoma skin cancer) that will limit patient survival over the duration of the trial.

	16. Any factor that suggests that the potential participant will not be able to adhere to the protocol.<br>17. Women of child-bearing potential including those with plans for post-menopausal in vitro fertilization or other reproductive technology.

4.2 Study Enrollment Procedures

TACT2 will enroll a population of stable, post-MI diabetic patients. The majority of patients, therefore, will be approached in the outpatient area, including:

i. Cardiology practices of investigators, colleagues, or referral sources
ii. Endocrinology practices of investigators, colleagues, or referral sources
iii. Cardiac rehabilitation services (particularly in Canada)
iv. Potential participants contacting clinical sites in response to IRB-approved advertisements
v. Potential participants contacting clinical sites as a result of interviews, lectures, and social media

Enrollment of Women and Racial and Ethnic Minorities
The TACT2 investigators are committed to recruitment of a representative sample of women and minority subjects as required by NIH, and we understand that failure to do so will decrease the validity and generalizability of study results. TACT2 is well positioned to achieve its goals of enrolling subjects representative of the US population. We are selecting patients for study that are age 50 or older, which has the corollary benefit of achieving a more equivalent distribution of men and women for whom coronary artery disease (CAD) is the leading cause of death. Moreover, we anticipate randomizing patients who predominantly have Type 2 diabetes (T2D). Compared with non-Hispanic whites, the risk of diagnosed diabetes is 70% higher among African Americans and 80% higher among Hispanics, which also has the corollary effect of helping us to achieve a sample that is sufficiently representative of these minority populations. TACT2 will target enrollment of at least 40% women and 18% minority subjects, which is in line with the demographics of prevalent myocardial infarction (MI) in the US population and the realities of enrolling women and minorities, as shown by our review of the literature.

Targeted Enrollment of Women
We are committed to enrolling sufficient numbers of women so that the results of TACT2, if positive, will be plausibly applicable to women. The target proportion of women to be enrolled in TACT2 is lower than the overall proportion of women in estimates from the 2010 US Census (51%),[36] but it is consistent with current estimates of the proportion of prevalent MI among women, especially those with diabetes. Based upon cardiovascular disease (CVD) surveillance from 1994-1997 in the NHLBI Atherosclerosis Risk in Communities (ARIC) Study, one of the largest NHLBI population cohort studies and CVD surveillance programs, 32% (n=4,253) of 13,068 hospitalizations for MI occurred in women.[37] More recent data from the 2014 Heart

Disease and Stroke Statistics update from the American Heart Association (AHA) reported a similar proportion of prevalent MI in women (34%).[38-39] Gore et al.,[40] based on the National Registry of Myocardial Infarction (NRMI), reported that 44.8% of incident MIs in diabetics occurred in women, with an 8.4% in-hospital mortality that was slightly higher than that of men. Perhaps more to the point of the attainable, the FREEDOM Trial[41] reported its results in 2012. This was an NIH study of revascularization strategies in diabetic patients with multivessel coronary disease. There were 1900 patients enrolled, and 29% were women (Similar to ARIC, above). The TACT2 leadership has carefully considered the population goal as reported by Gore, and the attainable goal from FREEDOM. While we believe we can improve on FREEDOM, we do not realistically believe we can enroll a population fully reflective of Gore et al. Thus, our goal of enrolling at least 40% women in the TACT2 trial is justified.[37-41]

Targeted Enrollment of Minorities
Our goal for enrollment of minorities is more reflective of the Gore data, which reported 18.2% minorities in the NRMI diabetes dataset. This proportion included 9% black and 3.4% Hispanics. Overall, the TACT2 enrollment target of 18% for minority patients is justified based on these data and is in accord with published data on the proportion of minority patients with prevalent MI used by the AHA, ACC-NCDR and VAMC.[37-43]

Additional Strategies to Enroll and Retain Women and Minorities
Current informal surveys of patients undergoing chelation have already led to suggestions for sites to consider infusions of multiple patients simultaneously in a socially compatible group setting, to consider gender-based infusion groups, particularly for women patients, and to consider extended hours or weekend infusions.

Strategies will be added or modified throughout the study, as formal surveys of patients are performed, and sites apply or discard different approaches. These plans will form part of a living document external to this Protocol, entitled "Plans to Identify, Recruit, Enroll, and Retain Women & Minorities."

5. STUDY INTERVENTIONS

5.1 Interventions, Administration, and Duration

Chelation/placebo infusions: The active infusion contains up to 3 g of Na_2EDTA adjusted based on eGFR, 2 g of magnesium chloride, 100 mg of procaine HCL, 2500 U of heparin, 7 g of ascorbate, 2 mEq KCl, 840 mg sodium bicarbonate, 250 mg pantothenic acid, 100 mg of thiamine, 100 mg of pyridoxine, and sterile water to complete 500 mL. Placebo infusions consist of 500 ml normal saline with 1.2% dextrose. Infusions are administered weekly for 40 weeks.[12] More details can be found in the Manual of Procedures (MOP).

The dose of edetate disodium in the infusion solution will be adjusted based onf estimated creatinine clearance and ideal body weight (IBW), but in no case will ever exceed 3 grams per infusion (3000mg):

EDTA dose (mg) = [50mg x (IBW x 1.33) x min (100, creatinine clearance)] ÷100

The IBW is calculated based on the Devine formula:[44]

- For men the weight is computed as 50 kg plus 2.3 kg for each inch of height over 5 feet (or 60 inches).
- For women the weight is computed as 45.5 kg plus 2.3 kg for each inch of height over 5 feet (or 60 inches).
- Actual body weight is used whenever it is less than computed ideal body weight.

Correction for creatinine clearance should only be done if creatinine clearance is less than 100.

Creatinine clearance will be computed by using a modified version of the Cockcroft-Gault equation:

$$\text{Creatinine Clearance (ml/min)} = \frac{(140 - \text{age}) \times (\text{IBW} \times 1.33)}{(72 \times \text{Cr})}$$

Creatinine Clearance = computed renal glomerular filtration rate in ml/min
Age = patient's age
IBW = computed weight in Kg
Cr = serum creatinine in mg/dL
For women, multiply the above result by 0.85

EDTA dose is automatically adjusted by the electronic data capture system based on the most recently calculated creatinine clearance value. If a prescription change for EDTA is required, the pharmacy is automatically notified by the electronic data capture system. The pharmacy will adjust the EDTA for the patient's next scheduled infusion.

Serum calcium is corrected by albumin level to generate a corrected calcium value that is an estimation of ionized calcium[45] as follows:

corrected calcium = serum calcium + (0.8 x [normal serum albumin - patient's albumin])

calcium= mg/dL

albumin= g/dL

- When corrected calcium is greater than 8.5 mg/100mL, the infusion is administered over 3 hours.
- When corrected calcium is between 8 and 8.5, the infusion is administered over 4 hours.
- When corrected calcium is less than 8, the next infusion is not administered and serum calcium is rechecked at least 1 week later. When corrected calcium is within acceptable range, infusions continue. This usually leads to a 2 week delay in infusions.

OMVM/placebo: The OMVM consists of 3 caplets, twice daily for a total intake of vitamin A 25000 IU, vitamin C 1200 mg, vitamin D3 100 IU, vitamin E 400 IU, vitamin K1 60 µg, thiamin 100 mg, niacin 200 mg, vitamin B6 50 mg, folate 800 µg, vitamin B12 100 µg, biotin 300 µg, pantothenic acid 400 mg, calcium 500 mg, iodine 150 µg, magnesium 500 mg, zinc 20 mg, selenium 200 µg, copper 2 mg, manganese 20 mg, chromium 200 µg, molybdenum 150 µg, potassium 99 mg, choline 150 mg, inositol 50 mg, para-aminobenzoic acid 50 mg, boron 2 mg, vanadium 35 µg, citrus bioflavonoids 100 mg[12]. Placebo caplets consist of microcrystalline cellulose, stearic acid, croscarmellose sodium, silica and magnesium stearate. OPADRY® II complete film white coating, magnesium stearate and vanilla flavor are used to coat both active and placebo pills. These caplets are taken for the entire duration of the study.

Low-dose vitamins: The low-dose supplements are taken by all patients to prevent any deficiency syndromes from chelation of essential minerals or vitamins. They consist of: vitamin B6, chromium, copper, manganese, and zinc. As was done in TACT, all patients will receive a low-dose regimen consisting of a single pill to be taken during the infusion regimen, stopping at 1 year at the latest. The low-dose regimen consists of: vitamin B6 25 mg, zinc 25 mg, copper 2 mg, manganese 15 mg, and chromium 50 µg.

5.2 Handling of Study Interventions

Infusion Encounter

The clinical unit will administer infusions. Infusions will be received in a refrigerated individual overnight mailer that will contain an IV bag and 2 syringes, each labeled for the individual patient. Typically, the infusion kit is received the day before the infusion is scheduled, and is good for 72 hours if unmixed. Once mixed, the refrigerated infusion will be good for 24 hours only. The specifics will be covered in detail in the MOP.

Each infusion encounter will be preceded by a brief interval history with specific emphasis on cardiac symptoms and clinical events, including hospitalizations. Vital signs will be measured before and after the infusion prior to discharge from the infusion site. A brief cardiopulmonary exam will be recorded at screening and at the end of each infusion. If there is weight gain of 3 lbs or more since the last infusion, or 5 lbs or more since the screening evaluation, the patient will be evaluated for heart failure. A trained study coordinator or infusion nurse may perform this examination. If there is heart failure, the infusion will be delayed until the patient is treated. Symptoms occurring during the infusions will be elicited and recorded on the eCRF. Patients will take their diabetes medications as scheduled unless a blood draw is scheduled. In that case, patients will arrive after an overnight fast and the diabetes medications will be held until the morning blood draw prior to the beginning of the infusion. Patients may consume food and drink during the infusion.

Infusion Schedule

Infusions will be administered on a weekly basis for 40 weeks through a peripheral intravenous line over at least 3 hours. Due to vacations, travels, weather, and illness,

the infusion regimen can be extended but is expected to be completed by 1 year. Clinical sites, however, will be instructed that if a patient has not completed the infusions by 1 year, they should continue trying to get the patient to clinic until the 40th infusion is complete. The total number of infusions is key, not the weekly schedule on 1-year completion. **The overall goal of the treatment schedule is to deliver all 40 infusions; no matter how long it takes.** The DCRI Call Center will follow patients for clinical events, but not for infusion scheduling or other site-centered events beginning 6 and 12 months after randomization, and at 4 month intervals subsequently until the study ends.

Distribution of Vitamins
The TACT2 Central Pharmacy will be responsible for distributing the vitamins every four months to the individual patients. The study vitamins will be mailed directly to the subject's home. In order to better preserve the blind and improve vitamin compliance, the study vitamins will be packaged in one month bottles and in individual dose packs. Vitamin compliance will be estimated by the site personnel and the DCRI Call Center at specific intervals and entered into the Electronic Data Capture (EDC) system. The details will be covered in the MOP.

Managing Patient Safety During the Infusion Encounter
Blood samples for safety will be collected during infusion visits as seen in the schedule below.

Hypoglycemia – Among 839 chelation-treated patients in TACT receiving 27,382 infusions, there was 1 episode of hypoglycemia. However, given that all patients in TACT2 have diabetes, all sites will be instructed in how to suspect, diagnose, and treat hypoglycemia. Patients with diabetes are more likely to have hypo and hyperglycemia based on the nature of their disease and its treatment. There is no expectation that patients with diabetes are prone to hypoglycemia from active or placebo treatment.

Some patients using continuous glucose monitors (CGMs) may have incorrect readings due to the contents of the active/placebo infusion. This could result in either not giving insulin when it is needed or giving too much insulin. To avoid insulin-dosing errors due an incorrect CGM reading, patients will be directed do the following:

- Do not depend on the results of CGMs for insulin dosing while receiving an infusion and for up to 12 hours post infusion; finger stick measurements are recommended in that timeframe.

- Users of a closed loop insulin pump system should set the pump to manual mode for 24 hours starting at the time of the infusion, and use finger stick measurements in that timeframe.

Hypocalcemia – All sites will have training in how to recognize symptoms of hypocalcemia, and additionally have available calcium chloride or calcium gluconate for intravenous administration. Should hypocalcemia be suspected during an infusion, the

infusion will be stopped, and the patient will have blood drawn and be treated. The CCC should be called for a joint evaluation (with the site coordinator and site PI or designee) of the symptoms and result of the serum calcium drawn during the event before scheduling the next infusion. Symptomatic hypocalcemia requiring an ER visit was observed only once in TACT.

Fluid overload – Patients are excluded from TACT2 if they are thought likely to be intolerant to 500 mL fluid infusions weekly or had a heart failure hospitalization within the past 6 months.

When a patient gains more than 5 lbs from their screening weight or more than 3 lbs from their last visit, the site investigator or designee should evaluate the patient for heart failure. If, in the opinion of the site investigator or designee, the patient has heart failure, the infusion will not be started and the patient will be treated or referred for treatment. Heart failure throughout the trial will be considered an event of special interest and carefully tracked and compared between groups.

Recommended Safety Materials
Clinical Sites should ensure the following safety materials are available during administration of infusions:

- Snacks or juice for empiric treatment of hypoglycemia
- Intravenous dextrose 50%
- Intravenous calcium gluconate or chloride
- Glucometer for measuring glucose levels

5.3 Concomitant Interventions

TACT2 will be conducted at a variety of clinical sites and there may be a range of medical therapies employed for secondary prevention. Specific medications for post-MI and diabetes will be recorded, as will injectable bisphosphonates for osteoporosis. TACT2 encourages the use of the latest, evidence-based care for diabetes and post-MI secondary prevention. To this end, the prevalent guidelines will be summarized in study material and made available to the sites. Patients will be encouraged to have an endocrinologist involved in their diabetes care. The Diabetes Science Consultant will evaluate summary data on HbA1c. The CCC will evaluate post-MI guideline compliance based on reports produced by the DCC. Sites will receive a quarterly report regarding the proportion of patients receiving guideline-based post-MI care, and HbA1c. Those placing in the lowest quartile will be notified by the CCC, and assisted in improving their compliance with evidence-based post-MI treatment and diabetes treatment.

5.3.1 Optimal Concomitant Interventions

All post-MI evidence-based medications, specifically anti-platelets, statins, beta-blockers, ACE inhibitors or ARBs, and aldosterone blockers are encouraged, if indicated based on clinical need as assessed by the patient’s physician. All necessary cardiovascular procedures are allowed. All conventional diabetes management is permitted with a focus on optimal glucose control while avoiding episodes of

hypoglycemia. Recommended medical treatment for secondary cardiovascular prevention and diabetes management will form part of a living document outside the study protocol, that will be updated annually (Suggested Management of TACT2 Patients) and circulated to sites.

5.3.2 Prohibited Interventions Following randomization

i. Effective, FDA-approved, oral chelation at any time during the course of the trial.
ii. Intravenous chelation, outside of the TACT2 intervention, at any time during the course of the trial.
iii. Over-the-counter vitamins having any of the same components as do the TACT2 vitamins. In patients who are unwilling to discontinue outpatient vitamins, or whose participation in the infusion regimen is dependent on their continuing to take out of protocol oral vitamins, the CCC may grant a waiver based on discussion with site staff.
iv. Injectable vitamins such as the Myers' cocktail unless medically necessary (i.e. pernicious anemia). In patients whose participation in the infusion regimen is dependent upon their permission to receive a Myers' cocktail no more than once monthly, the CCC may grant a waiver based on discussion with site staff.

Exception: Vitamin D_3 prescribed for bone health, along with calcium, may be taken and will be recorded in the eCRF.

5.4 Adherence Assessment

Assessing infusion adherence: the site coordinators will enter in the eCRF if the infusion was completed. In cases where the infusion was started and not completed, the site coordinator will estimate whether more or less than ½ of the infusion was infused.

Assessing OMVM adherence: During the study, OMVM will be delivered every 4 months in bottles. Each dose pack will have 6 OMVM/placebo pills inside. Each bottle will have 30 dose packs. At designated intervals during the study, the patient's compliance will be assessed.

Assessing low-dose vitamin adherence: The low-dose regimen is to be taken during the infusion phase only. The subjects should take one low-dose pill daily. When patients are non-compliant with this regimen they will be advised by site staff regarding its importance.

6. STUDY PROCEDURES

6.1 Schedule of Evaluations

SCHEDULE OF EVALUATIONS									
		**Clinic Visits**							**DCRI Call Center Data Collection**
		Infusion Phase							Months 6, 12, 16, 20, 24, 28, 32, 36, 40, 44, 48, 52, 56, 60
	Screening	Every Inf	Inf 1	Inf 5	Inf 10	Inf 20	Inf 30	Inf 40	Every Call
Assess Inclusion / Exclusion Criteria	X								
Obtain Informed Consent and Medical Records Release Form	X								
Contact Information	X								
Demographics	X								
Medical History	X								
Complications of Diabetes	X					X		X	X
Laboratory Tests									
Creatinine	X			X	X	X	X	X	
Calcium	X			X	X	X	X	X	
Albumin	X			X	X	X	X	X	
Glucose	X					X		X	
HbA1c	X					X		X	
Microalbumin	X					X		X	
CBC/plt	X					X			
Liver Function	X								
Lipids	X							X	
Blood metals (pre/post infusions)									
Cadmium (urine)			X	X		X		X	
Lead (blood)			X	X		X		X	
Vital Signs									
Blood pressure	X	X							
Heart Rate	X	X							
Pulmonary check	X	X							
Height	X								
Weight	X	X							
Medications									
Concomitant medications	X			X		X		X	X
Study Vitamins				X	X	X	X	X	X
Endpoints									
Hospitalization		X							X
All-cause Mortality		X							X
Safety**		X							
Economic Aims									
EQ-5D	X			X		X		X	X
Resource Utilization		X							X

* EOS = end of study
**Safety assessment = every visit and 30 days post final infusion

6.1.2 Laboratory Procedures

The TACT2 laboratory procedures include safety monitoring, diabetes monitoring labs, and additional labs (metals and biorepository). Abnormalities in the safety lab results collected will be compiled and reported to the DSMB. Biosample processing, analysis, storage, and data management details are covered in the separate MOP for each laboratory.

In brief, there are 2 types of blood and urine specimens that will be sent for analysis:

1. Safety labs will be sent to study central laboratory.
2. Blood and urine for metals analyses and for preparation and storage will be sent to the TACT2 Biorepository. The Biorepository will be responsible for processing and storage of samples during the study and intermittent batch transfer of urine and blood samples to the analysis laboratory.

Safety Laboratory collection at the Clinical Site
Blood and urine samples will be collected at screening, infusion 5, 10, 20, 30 and 40. See Schedule of Evaluations in Section 6.1 for specific laboratory tests. The study central laboratory will provide the supplies for collection and packaging for pick-up. This is explained in greater detail in the MOP.

Biorepository blood and urine sample collection at the Clinical Site
Blood and urine samples will be collected at infusion 1, 5, 20 and 40 to measure metal levels and for long term storage. The biorepository will ship the collection kits to the clinical sites. This is explained in greater detail in the MOP.

6.2 Description of Evaluations

6.2.1 Screening Evaluation

Consenting Procedure
Patients agreeing to participate will be asked to provide informed consent, medical record release form, and the pharmacy agent designation form which permits the pharmacy to ship the study infusion to sites rather than to the patient. Patients will also agree to follow-up by the DCRI Call Center beginning six months after randomization. If a site closes prior to study end, the randomized subjects will be asked to re-consent using an addendum for DCRI Call Center continuation of follow-up calls. Study personnel obtaining consent are specifically trained to explain the study, answer questions and ascertain that the patient understands the key elements of the research.

The original informed consent, pharmacy agent designation and medical release forms will be maintained as a hardcopy at the site in the study file. Copies of the signed forms will be given to the patient, and a scanned copy of the informed consent and medical release form will be emailed or faxed to the DCRI Call Center. The pharmacy agent designation form will be emailed to the central pharmacy. The site will document the version and date of the signed consent in the eCRF.

Screening assessment, enrollment, and randomization

Screening Visit

After providing informed consent, the site will enter the patient in the EDC system where a unique identifier will be assigned. The patient will have the medical history reviewed by study staff, and blood and urine samples will be drawn. Eligibility will be based on the following:

- All inclusion criteria are met
- No exclusion criterion is present

Data collected will include relevant cardiac history, particularly of the qualifying MI (anterior or non-anterior), whether patients have Type 1 or Type 2 diabetes, complications of diabetes, and treatment of diabetes. Sites are encouraged to discuss any questions about qualifying criteria with the CCC. Medication data will include post-MI evidence-based medications and treatments for diabetes. Laboratory test results will be entered to characterize the patient population, in particular with regards to diabetes control, renal function, and safety parameters to be followed during follow-up.

Randomization and scheduling first infusion

A simple randomization scheme reflecting an equal allocation ratio to the four different treatment groups (1 active chelation / active OMVM: 1 active chelation / placebo OMVM: 1 placebo chelation / active OMVM: 1 placebo chelation / placebo OMVM) will maximize the power for detecting treatment differences at the time of the interim and final analyses.

Patients who are confirmed eligible to participate in TACT2 will be notified by the site study personnel. The patient will be randomly assigned to a blinded treatment group by the EDC system (this is the randomization date), and the first infusion visit will be scheduled. The allowable range of time during which all screening activity (defined by date of consent) must be completed is 30 days. After 30 days, if randomization has not taken place, screening labs must be re-drawn. The first infusion, should occur within 2 weeks of randomization.

6.2.2 First infusion visit

This visit will involve collection of urine and blood for metals and biorepository. The biorepository will provide specific labels identifying patient number, infusion number, sample type, including kits with metal-free urine and metal-free blood collection containers. More detail about blood and urine collection and shipping will be found in the MOP.

6.2.3 Subsequent Infusion Visits (2-40)

The detailed schedule and activities of the follow-up visits are covered in Section 6.1 (Table 2). Within the infusion period in Year 1, most visits are associated with an infusion number, and do not have a traditional time-window for performance.

During the infusion year, the only time-dependent visit landmarks in TACT2 are:

1. The first infusion, which should occur within 2 weeks of randomization.
2. In non-adherent patients who have stopped receiving infusions, safety labs will not be checked. However, the study site will make all possible efforts to bring these patients in for blood and urine metal collection as well as scheduled fasting glucose, HbA1c, and microalbumin. During these specimen visits, patients will also complete a clinical visit including history, and a brief exam. Subjects no longer receiving infusions are expected to return at week 5, 20, and 40 post-randomization for these visits. For patients who have stopped receiving infusions, a urine / blood sample within 4 months of the last infusion is informative regarding their metal levels at the time the patient stopped infusions. The CCC should be consulted for all non-adherent patients. The management of the non-adherent patient will be covered in detail in the MOP.

The data collected in each call is covered in the Table in Section 6.1.

6.2.4 Blinding

Blinding

The shipped and refrigerated infusion package will contain an ascorbic acid syringe (or ascorbic acid placebo if the patient is assigned to the placebo arm), one syringe with EDTA (or EDTA placebo if the patient is assigned to the placebo arm), and a bag for intravenous infusion with all the other components mixed (or a bag containing only normal saline if the patient is assigned to the placebo arm). EDTA in solution is clear and of a viscosity indistinguishable by clinical staff from that of water. Thus, the placebo-EDTA syringe will contain normal saline. The ascorbic acid solution is generally a pale yellow color, which, upon mixing (14 ml of ascorbic acid solution in 500 ml) becomes indistinguishable from the clear saline placebo solution. In addition, ascorbic acid, in the concentration provided by the manufacturer, is viscous and provides resistance to transfer into the infusion bag through a 21-gauge needle. A colored but translucent syringe will mask the color of the active ascorbate syringe. Regarding blinding procedures for the vitamin and mineral supplements, placebo and active treatment groups will take identical-appearing pills and capsules. The above blinding was proven successful in TACT.

Procedure for Unmasking

Unblinding is rarely justified or necessary. If the clinical site wishes to unmask for an adverse event, however, the clinical site will contact the CCC PI to discuss the clinical details of the case. Following these discussions, if the participant’s physician still desires unmasking, the CCC PI will contact the DCC Co-PI to request unmasking. The treatment assignment will be made available to the treating physician. The CCC PI will remain blinded, and a request will be made to the treating physician to maintain the patient blind. These requests will be submitted and handled on a case-by-case basis, but will be entered into the eCRF.

Resource Utilization Assessments
Health care resource use will be collected on the clinical trial eCRF covering the period from the first infusion through end of follow-up. For all hospitalizations, length of stay by intensity of care (e.g., number of step down, intensive care nights) will be recorded along with major procedures. Discharge disposition for each acute care stay (e.g., home, skilled nursing, rehabilitation, nursing home, death) will be documented. In addition, dates of care at non-acute care facilities (e.g., skilled nursing, rehabilitation, long-term care) will also be collected to the end of the study follow-up. The dates of significant outpatient diagnostic and therapeutic procedures will be recorded.

6.2.5 Completion/Final Evaluation

All of these assessments will be discussed in detail in site training and will be an important part of the MOP.

7. SAFETY ASSESSMENTS

Institutional Review Boards (IRBs)
All TACT2 sites will submit the study protocol, informed consent form, and other study documents to their corresponding IRB for approval. Any amendments to the protocol, other than minor administrative changes, must be approved by the TACT2 DSMB and each IRB before they are implemented.

7.1 Specification of Safety Parameters

TACT amassed a substantial body of safety data. Edetate disodium was associated with few side effects and there were no differences in serious adverse events compared with placebo.[13,14,34] The safety specifics are summarized below.

- **Calcium**. Hypocalcemia, defined as a calcium level less than 8.5 mg/dL prior to an infusion, was reported in 52 chelation patients (6.2%) and 30 placebo patients (3.5%) (P=0.008). One patient, non-diabetic, had symptomatic hypocalcemia associated with muscle cramping that led to an emergency department visit. With regards to bone health, there were 2 fractures in the patients receiving the full chelation strategy and 2 fractures in those receiving double placebo (P=1.00). Although no safety signal pertaining to bone health was found in TACT, the effect of chelation on calcium makes it appropriate to measure calcium during the infusion period, as well as designate fractures as an event of interest and specifically query for it at regular intervals in the clinic visits and the telephone follow-up in TACT2.
- **Kidney**. An abnormal creatinine during the infusion phase was reported in 3/839 (0.4%) chelation patients and 9/868 (1%) placebo patients (P=0.093). Because EDTA is renally excreted, creatinine will be followed during the infusion period.
- **Liver**. There were no differences in liver enzyme abnormalities in the chelation compared with the placebo group. Liver function will therefore not be monitored after the screening study.

- **Hematology**. There were no significant differences in abnormalities of hematologic parameters (white blood cells, hematocrit, and platelet count) in EDTA compared with placebo groups. Complete blood counts will be assessed at screening, infusion 20, and at infusion 40 or 1 year, whichever is earlier.

Safety in the projected TACT2 population of post-MI diabetic patients: In the patients with diabetes, chelation was as safe as in the non-diabetic population. There were 95 serious adverse events (non–endpoint events) in the population with diabetes mellitus (56 chelation placebo and 39 chelation active).

Safety – Oral multivitamins and multiminerals: Serious adverse events occurred in 124 (15%) vitamin recipients and 103 (12%) placebo recipients (difference, 3 percentage points [95%CI=0.7 to 5.7 percentage points]). Adverse events included 12 (1.4%) incident neoplasms in the vitamin group and 11 (1.3%) in the placebo group (difference, 0.1 percentage point [95%CI=0.8 to 1.3 percentage points]). No evidence suggested harm from vitamin therapy in any category of adverse events.

Routine safety labs: Study staff will review all laboratory values, including safety labs and diabetes monitoring labs. Patients and their physicians will be informed when laboratory values are abnormal if, in the judgment of the research team, a change in medical therapy is warranted, or the abnormal laboratory value has diagnostic value of clinical importance. These procedures are covered in detail in the MOP.

7.2 Methods and Timing for Assessing, Recording, and Analyzing Safety Parameters

Safety Labs and the TACT2 infusion regimen.
The timing of safety labs is covered in section 6.1 (Schedule of Evaluations)

Renal function. Edetate disodium is excreted by the kidneys, and the dose of edetate disodium administered is adjusted based on estimated creatinine clearance.[46] There was no difference in renal function between groups at the end of 30 infusions in TACT. In TACT2, each assessment of serum creatinine will potentially lead to a change in total edetate disodium dose for the next series of infusions, with the following 2 “trigger points.”

1. If estimated creatinine clearance is reduced by 25% or more compared with baseline, there will be a “lab delay” transmitted to the site and to the CCC and DCC. The site will be contacted and asked to review whether there have been medication or over-the-counter supplement changes as well as changes in clinical status that might account for a reduction in renal function, and the lab will be repeated. The dose of edetate disodium will be recalculated before re-administration. This will be entered in the EDC system.
2. If creatinine doubles or exceeds 2.5 mg/dL, whichever is lower, the site will be contacted and asked to review whether there have been medication or over-the-counter supplement changes as well as changes in clinical status that might account for a reduction in renal function, and the lab will be repeated. Infusions

will be resumed when creatinine is <2.0 mg/dL and the edetate disodium dose recalculated at that time. This will be entered in the EDC system.

Calcium levels. Edetate disodium chelates ionized calcium and can lead to hypocalcemia. In TACT, however, there was only one episode of symptomatic hypocalcemia leading to an Emergency Room visit. Calcium levels will be monitored, and if corrected calcium is between 8.0 mg/dL and 8.5 mg/dL, infusions will be administered over 4 hours, instead of 3 hours. If corrected calcium is below 8.0 mg/dL, there will be a lab delay, and infusions will be held until calcium is 8.0 mg/dL or higher, based on bi-weekly blood draws. This information will be entered in the EDC system.

Metals labs
The handling of the trace metals analyses and biorepository will be available in the MOP for each of these study units. If the lead or cadmium levels measured by the Trace Metals Laboratory are outside the upper boundaries for American Conference of Governmental Industrial Hygienists (ACGIH)[47] (after a repeated measurement at the Trace Metals Laboratory) the clinical site and the CCC will be notified. In most cases, this will require repeating the levels in a commercial lab. If levels are still high, the participant will be recommended to contact their local public health department who will follow standard procedures for the evaluation of metal exposures. Because removal from exposure is the primary treatment, this should not interrupt the TACT2 infusions. The study sample is unlikely to have any such values.

Clinical Assessments
In TACT, the principal concern that emerged related to the weekly infusions of 500 ml of fluid and the potential precipitation of heart failure (HF). Patients are not eligible for TACT2 if they have had a hospitalization for heart failure in the 6 months preceding initial eligibility assessment. Nonetheless, like in TACT, in order to reduce ongoing patient risk, weight will be monitored at each infusion. If there is weight gain of 3 lbs or more since the last infusion, or 5 lbs or more since the screening evaluation, the patient will be evaluated for heart failure. A trained study coordinator or infusion nurse may perform this examination. If there is HF present, this will be entered into the EDC, the infusion delayed for 1 week, and a suitable treatment regimen developed with the patient's clinical physician.

7.3 Adverse Events and Serious Adverse Events

Adverse Event (AE)
An adverse event is any undesired, noxious or pathological change in a patient as indicated by signs, symptoms, or laboratory changes that occur in association with the use of trial intervention/medication, whether or not considered drug or biologic related. This definition includes intercurrent illness or injuries, exacerbation of existing conditions, psychological events, psychosocial events, associated temporally associated with the use of a pharmaceutical product or study intervention. Pre-existing conditions, which worsen during a study, are considered adverse events and can become serious if they fulfil one of the seriousness criteria described below.

Note: Diseases, signs, symptoms, and/or laboratory abnormalities already existing at study admission are not considered adverse events when observed during the trial unless they represent an exacerbation in intensity or frequency. (Definition modified from ICH-E2B)

Assessment of Adverse Event Intensity
The intensity of an adverse event is an estimate of the relative severity of the experience made by the investigator based on his or her total clinical experience and familiarity with the literature. The maximal intensity reported during the evaluation period should be recorded. The intensity of adverse events will be characterized as mild, moderate or severe as follows:

Mild	Events are usually transient, require no special treatment, and do not interfere with the patient’s daily activities.
Moderate	Events usually introduce a low level of inconvenience or concern to the patient and may interfere with daily activities, but are usually ameliorated by simple therapeutic measures.
Severe	Events interrupt a patient’s usual daily activity and generally require systemic drug therapy or other treatment.

Assessment of Causal Relationship
A medically-qualified investigator must assess the relationship of any AE to the use of study drug, based on available information, using the following guidelines:

1. Related – There is a reasonable possibility that the adverse event may have been caused by the study intervention
2. Not Related – There is not a reasonable possibility that the adverse event may have been caused by the study intervention

Adverse event collection and recording procedures must be designed to meet DSMB review and regulatory submission requirements, and ensure drug safety.

Expectedness
The expectedness of an adverse event or suspected adverse reaction shall be determined according to the most current investigator’s brochure or product label. “Expected” is defined as an adverse drug experience, the specificity and severity of which is consistent with the current investigator brochure or the risk information in the investigational plan. This term relates only to the drug, not the patient’s underlying condition. Any AE that is not identified in nature, severity, or specificity in the current study drug reference document(s) (e.g., investigator’s brochure) is considered unexpected. Events that are mentioned in the investigator's brochure as occurring with a class of drugs or as anticipated from the pharmacological properties of the drug, but not specifically mentioned as occurring with the particular drug under investigation are considered unexpected. For example: Hepatic necrosis would be unexpected if the

investigational brochure or plan only referred to elevated hepatic enzymes or hepatitis. (Adapted from 21 CFR Part 312. 32)

Serious Adverse Event (SAE)
An adverse event or suspected adverse reaction is considered serious if it results in any of the following outcomes:

1. Death
2. Life-threatening
3. Persistent or significant disability/incapacity,
4. Requires or prolongs hospitalization
5. Congenital anomaly/birth defect
6. Important medical events that may not result in death, be life-threatening, or require hospitalization may be considered serious adverse events when, based upon appropriate medical judgment, they may jeopardize the patient and may require medical or surgical intervention to prevent one of the outcomes listed in this definition. *(Source CFR: 21 CFR 312.32)*

Laboratory Test Abnormalities
For laboratory test abnormalities that meet the definition of an SAE, that required the subject to have the investigational product discontinued or interrupted or required the subject to receive specific corrective therapy, the clinical diagnosis rather than the laboratory term will be used by the reporting investigator (e.g., anemia versus low hemoglobin value).

Life-Threatening
Life-threatening refers to any adverse event that places the patient at immediate risk of death from the reaction as it occurred. It does not include a reaction that, had it occurred in a more severe form, might have caused death. *(Source CFR: 21 CFR 312.32)*

Requires or Prolongs Hospitalization
A patient must be admitted to the hospital for a period greater than 24 hours, for the hospitalization to be considered a serious adverse event. Elective hospital admissions, scheduled prior to the study, are not considered serious adverse events unless the hospitalization is prolonged. Planned admissions (as part of a study), hospitalizations for less than 24 hours, hospitalization for an elective procedure, and Emergency Room/Department visits are not considered serious adverse events. Exception: Any disposition of the patient to the hospital or emergency room within 24 hours following study drug (infusion) therapy.

7.4 Reporting Procedures

The SAE reporting flow chart will be included in the MOP.

Protocol Specific Exceptions to SAE Reporting
Specified outcome events in the study will be recorded in their respective appropriate modules of the eCRF. These events and specific corresponding symptoms will NOT require reporting as serious adverse events on the SAE eCRF. Specifically, the events covered in this exception are as follows:

1. Myocardial infarction
2. Stroke
3. Coronary revascularization
4. Hospitalization for unstable angina

Serious Adverse Event Reporting
The site investigator is responsible for monitoring the safety of participants enrolled into the study. All SAEs must be reported from the time of randomization through 30 days post final infusion, using the electronic data capture system. Non-serious adverse events will not be collected on the eCRF. All SAEs whether or not deemed drug-related or expected must be reported by the investigator or qualified designee within 24 hours of first becoming aware of the event. The investigator or qualified designee will enter the required information regarding the SAE into the appropriate module of the eCRF. If the eCRF system is temporarily unavailable, the event, including the investigator-determined causality to study drug will be reported via the back-up paper SAE form to DCRI Safety Surveillance at 1-866-668-7138. Upon return of the availability of EDC system, the SAE information must be entered into the eCRF.

Adverse events that are serious and drug (infusion) therapy related or result in death will be reviewed by the DCRI Safety Surveillance Department Medical Monitor to assess medical clarity and unexpectedness. DCRI Safety Surveillance will review all SAE data including all deaths, within 1-2 business days to:

1. Ascertain seriousness
2. Ascertain drug (infusion) therapy causal relationship
3. Verify that all data are complete
4. Follow-up with the site for incomplete data and/or data clarification.

This will include, but will not be limited to, ensuring that serious criteria have been met and the SAE data received are reviewed, entered and coded using the MedDRA coding dictionary.

DCRI Safety Surveillance will notify the study sponsors (NIH and TACT2 Principal Investigators from Mount Sinai and Duke Clinical Research Institute), and Data Safety Monitoring Board Chairman (DSMB)) in a blinded fashion, within 1 business day of receipt of the initial notification of the SAE, of:

- All serious adverse events that are drug (infusion) therapy related – defined as taking place within 24 hours of an infusion, and attributed to the infusion by the site PI or by DCRI Medical Monitor, and/or
- All SAEs resulting in death

The DCRI Safety Surveillance will provide the DCRI Regulatory Services with the event specific forms necessary to report the expedited adverse event according to country specific regulatory guidelines. This will include all deaths assessed by the DCRI Medical Monitor as warranting expedited reporting to the regulatory authorities and all adverse events that are serious, unexpected and drug (infusion) therapy related (as assessed by the site investigator, DCRI Medical Monitor and TACT2 study leadership).

Procedures for Enhanced Reporting of Specific Adverse Events
DCRI Safety Surveillance will also report specific adverse and/or serious adverse events, not otherwise eligible for expedited SAE reporting as in Section 7.2, within 2 business days to the DSMB or its designee, and the NIH Program Officers. The adverse events for enhanced scrutiny include:

1. Heart failure hospitalization during the entire infusion phase of the patient's participation in the study, not otherwise subjected to expedited SAE reporting.
2. Any disposition of the patient to the hospital or emergency room within 24 hours following study drug (infusion) therapy, not otherwise subject to expedited SAE reporting.

These events would not be eligible for expedited SAE reporting if the causality criterion were not met. Notification of these events will be made electronically.

7.5 Follow-up for Serious Adverse Events

All reported SAEs will be followed until resolution, stabilization or until 60 days after the last patient enrolled in the trial completes the study drug (infusion) therapy. At the time of database lock, unresolved SAEs may be closed (final outcome assigned as "unresolved") per the discretion of the DCRI Medical Monitor and/or the DCC PI. The site investigator will be responsible for reporting adverse events and unanticipated problems involving risks to subjects to their local IRBs/IECs in accordance with local regulations.

7.6 Safety Monitoring

Data and Safety Monitoring Board
TACT2 will have an independent DSMB appointed by and reporting to NIH. The DSMB will be expected to meet with the study leadership at least 2 times per year to review study progress and overall safety. For ethical reasons, an interim examination of key safety and endpoint data will be performed during the course of the trial. A separate DSMB charter outlining the operating guidelines for the committee and the protocol for evaluation of data will be created prior to study enrollment and agreed upon during the initial meeting of the DSMB.

8. INTERVENTION INTERRUPTION

Reasons for Withdrawal/ Interruption
There may be reasons for subject withdrawal and/or interruption of the study infusions and/or OMVM.

Some possible reasons are:

- Died
- Lost to follow-up
- Informed consent withdrawal

Subject adherence and retention strategies will be included in the site personnel training. Site personnel will be encouraged to have weekly communications with enrolled subjects as a means of continued participation. The site investigator should make every attempt to accommodate the subject to continue the therapy where feasible and encourage subject to allow follow-up by the DCRI Call Center.

Withdrawal Procedures
Site study personnel must immediately communicate, with the CCC PI, all information from subjects that express desire to stop study treatment (either infusions or OMVM), follow-up or withdrawing informed consent. When all attempts by the site study personnel to accommodate the subject are unsuccessful, the final disposition of a subject withdrawal from study treatment and/or follow-up will **require** approval by the CCC PI. See the MOP for details.

9. STATISTICAL CONSIDERATIONS

9.1 General Design Issues

Analysis of the TACT2 2x2 factorial trial will be based on intention to treat (ITT). That is, participants will be analyzed (and endpoints attributed) according to the treatment strategy to which participants are randomized, regardless of subsequent additional post-randomization treatment and medical care. Statistical comparisons will be performed using two-sided significance tests. Treatment dropouts and consent withdrawals will be tracked by verifiable means within the eCRF at each follow-up interval. Baseline demographic and clinical variables will be summarized for each randomized arm of the study. Descriptive summaries of the distribution of continuous variables will be presented in terms of percentiles (e.g., median, 25th and 75th percentiles) along with means and standard deviations. Categorical variables will be summarized in terms of frequencies and percentages.

9.2 Sample Size and Randomization

Participants will be randomized in a 1:1:1:1 allocation ratio using simple randomization. The study was originally designed to enroll a total of 1200 participants with a minimum follow-up of 12 months. The DSMB conducted a blinded review of enrollment aggregate event rates, and projected event rates in July 2019. Following this blinded review, the

DSMB recommended a reduction in the target sample size to 1100 participants and an increase in the minimum follow-up from 1 year to approximately 2.5 years. Due to slowed enrollment related to the COVID-19 pandemic, in the fall of 2020, the DSMB and the NIH leadership agreed to a proposal to complete enrollment by 12/31/2020 with an allowance to follow the study participants until there are enough primary endpoint events to maintain 85+% power.

Several design factors and research objectives have been considered in developing an appropriate sample size for the TACT2 study. First, patient enrollment and follow-up has been determined so there would be a sufficient number of endpoints to provide a high degree of confidence for testing the primary hypothesis. Second, the statistical power for secondary endpoints has been considered. Finally, the sample size has been determined to provide a reasonable level of confidence for detecting clinically important differences in outcome between treatment strategies even if current projections of event rates and hypothesized differences in clinical outcomes between the treatment groups prove to be optimistic.

Table 3 shows the required number of endpoint events to have 80% to 90% power with hazard ratios varying from 0.50 to 0.75. The two factors (EDTA chelation and OMVM) are assumed to be additive for the sample size calculations. Based on TACT we anticipate a hazard ratio of 0.70 (active chelation vs. placebo chelation) to determine the sample size requirements. This hazard ratio assumption is very conservative compared to the observed effect in TACT. Assuming a two-sided 0.05 type I error rate and approximately 5% consent withdrawal or loss-to-follow-up per year, the final sample size of 1000 participants will provide approximately 85% power to detect a hazard ratio of 0.70 for the primary endpoint of time to all-cause mortality, myocardial infarction, stroke, coronary revascularization, or hospitalization for unstable angina comparing active chelation vs. placebo chelation.

Table 3. Required number of events

	HR=0.50	HR=0.55	HR=0.60	HR=0.65	HR=0.70	HR=0.75
80% power	65	88	120	169	247	379
85% power	75	100	138	194	282	434
90% power	87	118	161	226	330	508

Calculations performed using nQuery 7.0 and assume a 0.05 type I error rate (two-sided) with 1:1 randomization.

For other exploratory endpoints, we outline a general power justification for comparisons to be made among the four arms of the 2x2 factorial design. We anticipate that roughly 1000 participants will have blood and urine samples available from baseline and one or more follow-up time points. A sample size of 250 participants per group will provide 80% or greater power for any between group differences greater than ¼ of a standard deviation. These calculations are based on a two-sample t-test and assume a two-sided type I error rate of 0.05.

9.3 Definition of ITT Population

The ITT population will correspond to all randomized participants. The primary and secondary analyses will follow the ITT principle.

9.4 Interim Analyses and Stopping Rules

For ethical reasons, interim examinations of key safety and process data will be performed at regular intervals during the course of the trial. It is anticipated that the DSMB will meet at 6-month intervals to review the accumulating data. Prior to performing interim analyses, aggregate event rates will be assessed to compare predicted vs. actual event rates. The DCC will create regular reports to track patient enrollment reports, rates of adherence with the assigned treatment strategy, and frequency of protocol violations. Prior to each meeting, the data coordinating center will conduct any requested statistical analyses and prepare a summary report along with the following information: patient enrollment reports, rates of adherence with the assigned treatment, and description of SAEs (statistical comparisons of the randomized arms with respect to these SAEs will use chi-square or other appropriate 2-sample or >2-sample methods). Safety data will be reported by all 4 treatment groups as well as active chelation vs. placebo chelation and active OMVM vs. placebo OMVM. The DSMB will review data partially masked by study group (such as X vs. Y and not active chelation vs. placebo chelation).

Safety reports will be prepared for the DSMB approximately once every 3 months. Futility and efficacy monitoring will focus solely on the active chelation vs. placebo chelation comparison. The DSMB will review the active OMVM vs. placebo OMVM data for safety purposes only.

For futility monitoring, TACT2 will employ a modification of the inefficacy monitoring guideline of Freidlin, Korn, and Gray[48] to stop the trial if the chelation strategy is not beneficial. We plan to use a conservative boundary, denoted as LIB0, along with a harm look at 25% of expected information. This approach will include 4 interim looks scheduled at roughly 25%, 40%, 60%, and 80% of expected information. With the proposed design, a total of roughly 280 primary endpoint events are expected and the first interim review for futility and efficacy will be scheduled after approximately 70 primary endpoint events have been observed. The harm look at 25% of expected information would suggest stopping the trial if the data suggests harm for the chelation strategy vs. the placebo chelation with p-values <0.05 for both the primary outcome and the all-cause mortality endpoint. For the interim reviews at 40%, 60%, and 80%, the inefficacy guideline suggests stopping the trial if the chelation infusion strategy has a hazard ratio > 1.0 compared to placebo for both the primary outcome and the all-cause mortality endpoint. This inefficacy monitoring approach will result in a trivial loss of power and requires no sample size adjustment.

The method of Haybittle and Peto[49,50] will be proposed as the primary guide for interpreting interim efficacy analyses. The timing of the interim efficacy analyses will correspond to the futility monitoring reviews (i.e. at roughly 25%, 40%, 60%, and 80% of expected information). The proposed efficacy monitoring guideline requires large critical

values (Z=3, $p \leq 0.001$) for every assessment until the planned final analysis. The TACT2 investigators suggest that both the primary outcome and the all-cause mortality outcome are evaluated at the interim reviews with a focus on the comparison of active chelation vs. placebo.

The CCC PI, DCC PIs, CCC staff, clinical study Co-PIs, site investigators, NIH Program Staff and study participants will remain blinded until database lock. Limited staff will be unblinded to handle randomization codes, deliver the interventions to sites, and prepare DSMB reports. The statistical staff responsible for preparing DSMB reports will not directly interact with the clinical team that delivers care to the study participants or the CEC that determines clinical events.

9.5 Outcomes

9.5.1 Primary Outcome

The primary outcome is the time to the first occurrence of all-cause mortality, myocardial infarction, stroke, coronary revascularization, or hospitalization for unstable angina.

9.5.2 Secondary Outcomes

The three secondary outcomes of TACT2 are:

- the overall rate of events due to all-cause mortality, myocardial infarction, stroke, coronary revascularization, or hospitalization for unstable angina events
- the time to the first occurrence of cardiovascular mortality, recurrent myocardial infarction, or stroke
- the time to all-cause mortality

The TACT2 CEC will review all primary and secondary endpoints.

9.6 Data Analyses

Primary Endpoint Analysis

The primary objective of TACT2 is to determine if the chelation-based strategy increases the time to composite of all-cause mortality, myocardial infarction, stroke, coronary revascularization, or hospitalization for unstable angina compared to the placebo chelation strategy. The statistical comparison will be based on the time from randomization to the first occurrence of all-cause mortality, myocardial infarction, stroke, coronary revascularization, or hospitalization for unstable angina. The Cox proportional hazards regression model will be used to assess outcome differences between the treatments. An initial Cox model will include indicator variables for the active chelation and active OMVM groups, an interaction term for the active chelation and active OMVM, age, sex, and use of insulin. If the interaction term for the active chelation and active OMVM is not statistically significant at the two-sided 0.05 level, the primary analysis will be based on a Cox model which includes indicator variables for the active chelation and

active OMVM groups, age, sex, and use of insulin. The chelation treatment effect will be summarized using hazard ratios and associated 95% confidence intervals. In addition to the statistical hypothesis testing, Kaplan-Meier[51] survival estimates will be constructed based on the time from randomization to the first primary event occurrence.

The OMVM treatment effect from the Cox model will be estimated using the Cox model described above. Given the results of TACT and the focus on chelation therapy, the interpretation of the OMVM treatment effect will be considered exploratory.

Assessment of the proportional hazards assumption will be evaluated using visual analysis (of the survival and log {-log (survival)} functions), as well as tools within the “proportionality test” statement of Proc Phreg in SAS.

Secondary Endpoint Analysis

The analyses for the time-to-event secondary endpoints will be similar to those outlined for the primary endpoint using the time from randomization through the first occurrence of any component of a specific secondary endpoint (or censoring) as the response variable, and assessing group differences using the Cox proportional hazards model. The effect of the chelation treatment strategy vs. placebo chelation on these time-to-event secondary endpoints will be summarized using hazard ratios (with associated confidence intervals) computed from the Cox model. Kaplan-Meier curves will be constructed to display the cumulative event rates of the treatment groups.
To test the individual treatment strategies against the placebo chelation – placebo OMVM group, we will add three indicator variables to the Cox proportional hazards model described above.

Recurrent event analyses encompassing the overall event rate of endpoints for each patient.

The components of this endpoint will include all-cause mortality, myocardial infarction, stroke, coronary revascularization, or hospitalization for unstable angina events. We plan to analyze this endpoint using two statistical techniques. The two approaches are:

a) A generalization of the Cox model to handle recurrent events, developed by Anderson & Gill, with robust standard errors to account for heterogeneity.[52,53]
b) A marginal modeling approach developed by Wie, Lin, and Weissfeld.[54]

The Anderson-Gill approach will be considered the primary analysis for the total number of all-cause mortality, myocardial infarction, stroke, coronary revascularization, or hospitalization for unstable angina events endpoint.

Exploratory Endpoints

The comparison of costs will test mean direct medical costs incurred during the study (index infusion through end of follow-up). To account for staggered entry and incomplete follow-up, costs will be adjusted using the approach of Bang and Tsiatis.[55] Statistical comparisons will be made using a normal approximation with standard errors

estimated using the bootstrap approach. The probability of differences in cost greater than thresholds of policy interest (such as $1000, $2000, or $5000) will be calculated. Differences in cost by treatment group will be interpreted in the context of the trial clinical results, looking for both consistency and plausibility. Follow-up costs will be presented both overall and by category (e.g., inpatient readmission, outpatient procedures, concomitant medications, non-acute institutional care). Cumulative costs over the study period between treatment groups will be compared. Descriptive comparisons of cost according to clinical variables defining subgroups of interest will also be performed.

The analyses for toxic metals in blood and urine in the TACT2 population will proceed in three phases. First, we will evaluate if the chelation regimen has the effect on metal internal dose that we have postulated. To do this, we will compare body metal levels (lead in blood, pre-chelation urine lead and cadmium, and post-chelation urine lead and cadmium, in separate analyses) between active and placebo chelation groups at baseline and over the treatment phase of TACT2. Second, we will relate those levels to changes in the primary clinical endpoint (a composite of death from any cause, recurrent MI, stroke, coronary revascularization, or hospitalization for unstable angina) caused by chelation therapy relative to placebo. Finally, we will perform the exploratory analyses described below.

Over the 1-year period, we hypothesize there will be no change in metal levels in the placebo chelation group and significant reductions in the active chelation group. We also postulate these patterns of change in metal levels will explain, in a statistical sense, the clinical benefits we expect to see in TACT2 (the way LDL-cholesterol level lowering “explains” the benefits of statin therapy) using three complementary analytic approaches: 1] prespecified subgroup analyses (patients in the highest tertile of blood lead, urine cadmium, and post-chelation urine lead and cadmium at infusion 1, will show significantly larger treatment benefits (chelation vs. placebo) than patients with lower baseline levels), 2] landmark analyses (patients in the chelation arm with lower metal levels after 40 infusions/1 year of chelation will show significantly larger clinical benefits than patients with higher levels), and 3] we will apply causal inference modeling to evaluate whether the pattern of metal changes (lead in blood and cadmium in urine) in the chelation arm over the treatment period of TACT2 statistically “explains” the variation in clinical outcomes responsible for the hypothesized superiority of the chelation strategy.

We will also do exploratory analyses to examine the clinical implications of different patterns of metal removal over time on the benefits of chelation therapy (can metal levels be used as a biomarker to guide the number of chelation sessions needed to maximize clinical benefit from this therapy?) and to examine the effects of the oral multivitamin/mineral treatment regimen versus placebo vitamins on metal levels and on the effectiveness of chelation in reducing those levels (does the TACT2 vitamin regimen augment the ability of the chelation regimen to remove metals, or might there be a different mechanism of action altogether?). The latter case could be inferred if there were clinical benefits of OMVM, but no changes in lead and cadmium levels.

Subgroup Analyses
To examine the heterogeneity of the treatment effect, subgroup analyses for the primary and secondary endpoints will be performed in order to explore whether the treatment effect is consistent across subgroups. Subgroup analyses to evaluate variation in the effect of treatments will be performed using interaction terms within the Cox proportional hazards model. For binary variables, we will include the subgroup factor as a stratification variable within the Cox regression model. This model structure will decrease the reliance on the proportional hazards assumption. Additionally, treatment effects within each categorical subgroup will be examined separately using Cox proportional hazards models. Event rates by treatment and HRs with 95% confidence intervals will be reported for each subgroup. Forest plots will be generated displaying the estimated hazard ratios and 95% confidence intervals for each subgroup. For subgroups defined using continuous variables, the analysis based on the continuous form will be considered primary but for display purposes these variables can also be categorized.

We have pre-specified sex, minorities, age greater than 70, myocardial infarction location (anterior MI or not), type of diabetes (1 or 2), insulin treatment of diabetes, use of statin therapy at baseline (for the OMVM analyses only), known peripheral artery disease at baseline as key subgroups of interest. There are also pre-specified analyses by tertiles of lead and cadmium as indicated above. The examination of these pre-specified subgroups will include formal tests of interaction within the Cox regression model.

10. DATA COLLECTION AND QUALITY ASSURANCE

10.1 Data Collection Forms

A validated Electronic Data Capture (EDC) system will be used for this study. The majority of the study data will be transcribed by study personnel from the source documents onto an eCRF and transmitted in a secure manner to the DCC. All data relating to the study must be recorded in eCRFs developed by DCRI and approved by the study sponsor. Data must be entered into eCRFs in English. The investigator’s electronic signature, a compliant platform (21 CFR Part 11) is recorded in the database as verification that all data are complete and accurate.

10.2 Data Management

Data will be processed using a validated computer system conforming to regulatory requirements.

Computerized data will be accessible only by password, and a centralized monitoring system will record and report all access to data. The DCRI computer network is protected by a firewall. Electronic CRFs (eCRFs) will be identified by study number only, to ensure participant anonymity. No participant identifiers will be used in the presentation of data. Except when required by law, participants will not be identified by name, personal identification number (e.g., social security number in US and Canada,

address, telephone number) or any other direct personal identifier in study records. This information will be retained by each individual center and will not be disclosed to the Clinical or Data Coordinating Center except as needed for centralized clinical, quality of life and economic follow-up of the participants. Participants will be informed that the study physician and his/her study team will report the results of study-related tests to the Clinical and Data Coordinating Center and to the NIH. Participants will be informed that their records may be reviewed in order to meet federal, state or regional/local regulations. Reviewers may include the CCC/DCC monitors, IRBs/ECs, the NIH, other government regulators as dictated by local law, or their delegates.

Records of patients, source documents, monitoring visit logs, eCRFs, inventory of study product, regulatory documents, and other sponsor correspondence pertaining to the study must be kept in the appropriate study files at the site. Source documents include all recordings and observations or notations of clinical activities and all reports and records necessary for the evaluation and reconstruction of the clinical study. These records will be retained in a secure file for the period required by the institution or site policy. Prior to transfer or destruction of these records, the DCC must be notified in writing and be given the opportunity to further store such records.

10.3 Quality Assurance

Greater detail will be found in the MOP for the TACT2 and the individual Manuals for the Metals Lab and Biorepository. Steps to be taken to ensure the accuracy and reliability of data include:

- the selection of qualified investigators and appropriate study centers.
- review of protocol procedures with the investigator and associated personnel before the study.
- periodic monitoring visits to clinical sites, central pharmacy, metals lab, and biorepository.
- written instructions will be provided for collection, preparation, and shipment of blood and plasma samples.
- electronic CRF completion guidelines will be provided and reviewed with study personnel before the start of the study.
- The DCRI CRA will review eCRFs for accuracy and completeness during site monitoring; any discrepancies will be resolved with the investigator or designee, as appropriate.

At regular intervals, all data will be transferred from the EDC database to SAS for statistical summarization, data description, and data analysis. Further cross-checking of

the data will be performed in SAS, and discrepant observations flagged and appropriately resolved through a data query system.

10.3.1 Training

Training procedures will be described in the MOP. Site training, in particular, will be provided via in-person study meetings as well as online training.

10.3.2 Quality Control

The Data Coordinating Center will perform internal database quality-control checks, and data audits throughout the course of the trial, as well as coordinate blinded repeat metals analyses in a statistically valid proportion of samples.

10.3.3 Metrics

The Clinical Events Committee at the Brigham and Women's Hospital will adjudicate all components of the primary endpoint except for coronary revascularization. Coronary revascularization will be clinically confirmed by the Clinical Events Committee.

10.3.4 Protocol Deviations

Documentation and management of protocol deviations will be the responsibility of the DCRI Site Management team. Incidences identified will be discussed with the site investigator and study team for corrective action and follow-up with their IRB.

10.3.5 Monitoring

The DCRI Site Management team will be responsible for:

- verifying that the appropriate assurances and certification of training in the protection of human subjects are in place prior to the initiation of any protocol.

- providing education to all site staff regarding the conduct of clinical studies according to good clinical practices.

- assisting sites in the development of informed consent templates to make sure that they contain appropriate elements.

- ensuring that the CRA monitors each site according to the approved comprehensive monitoring plan.

- assuring during the site initiation visit that the site has adequate facilities/staff integral to the successful operation of the study. The site initiation visit may also be utilized to conduct training regarding regulatory compliance or individual protocol procedures.

- tracking recruitment of patients entered updating relevant trial information on the trial web site.

10.4 Medical Information Coding

For medical information, the following thesauri will be used:

- Latest version of MedDRA for adverse events, and
- World Health Organization Drug Dictionary for concomitant medications.

11. PARTICIPANT RIGHTS AND CONFIDENTIALITY

11.1 Institutional Review Board (IRB) Review

All TACT2 sites will submit the study protocol, informed consent form, and other study documents to their corresponding IRB for approval. Any amendments to the protocol, other than minor administrative changes, must be approved by each IRB before they are implemented.

11.2 Informed Consent Forms

TACT2 sites will obtain informed consent from each study participant prior to randomization. Trained personnel as listed in the Delegation of Authority log will obtain consent. In all cases in which the patient does not have an understanding of English, sites will obtain an IRB approved translation of the study consent to present to the patient. Patients will have ample opportunity to review the consent form, ask questions, and make an informed decision on risks and benefits.

11.3 Participant Confidentiality

Overall policy: Any data, specimens, forms, reports, video recordings, and other records that leave the site will be identified only by a participant identification number (Participant ID, PID) to maintain confidentiality. All records will be kept in a locked file cabinet. All computer entry and networking programs will be done using PIDs only. Information will not be released without written permission of the participant, except as necessary for monitoring by IRB, the FDA, the NCCIH, and the OHRP.

Exceptions to the policy:

1. The Study Pharmacy, following standard regulatory procedures, will deliver a -specific prescription for each study infusion. As such, they will know PHI for each patient. The PHI for each patient will be kept in a secured pharmacy computer, behind a firewall, and will be known to a minimum number of pharmacy personnel.
2. The DCRI Call Center will make the initial patient follow-up by phone. As such, they will need to know PHI including patient name, next-of-kin, etc. Again, this information will be kept secured in the DCRI Call Center.

11.4 Study Discontinuation

Procedures for study discontinuation will be detailed in the TACT2 MOP.

12. COMMITTEES

The Steering Committee
The main governing body and decision-making committee for the trial and assumes overall responsibility for the design, quality control, and conduct of the study. All major scientific decisions will be discussed with the Steering Committee. The Steering Committee will review the baseline characteristics and pooled event rate after 70 primary endpoint events have occurred. The Steering Committee will determine whether the patient population or sample size needs to be modified.

Executive Committee
The Executive Committee is advisory to the Steering Committee and is responsible for objective evaluation for the course of the trial, design and conduct. Recommendations for all scientific decisions will be made to the Steering Committee.

Biorepository Committee
This committee will coordinate communication between Columbia University, the CDC and DCRI on aspects related to sample collection, shipments and laboratory analysis (for metals); keep track of shipments (both specimen collection kits and samples) and samples collected, received, processed, stored and analyzed (for metals); troubleshoot any problems as they arise and report to the TACT2 operations committee.

Operations Committee
This subcommittee of the Steering Committee will provide oversight for the day-to-day progress of the study.

Publications, Presentations and Ancillary Studies (PPAS) Committee
The objectives of this committee will be to facilitate timely dissemination of study findings, maintain high scientific standards for published material, prioritize the order of publication and presentations, and ensure equitable investigator participation and attribution of authorship. The committee will ensure publications are well-aligned with the trial's research agenda and are not redundant. The PPAS committee will review all proposals for data analysis, as well as research abstracts, presentations, and manuscripts before submission. The committee will also review proposals for ancillary studies. The committee will also ensure that each publication that meets NIH Open Access criteria is deposited in PubMed Central.

Committee for the Recruitment of Women and Minority Subjects
Recruitment of women and minority subjects is a critically important part of developing a clinical trial whose results can be generalized to the overall US population. The TACT2 leadership has therefore constituted a committee of experts to assist in enhancing recruitment of women and minority subjects. They will report to the Steering Committee

with suggestions as to how to increase the enrollment of women and minorities. Some of these activities are covered earlier in this document.

Data-Driven Trial Management Committee
The objectives of this committee will be to:

- review the established mapped key risk indicators and triggers (thresholds)
- modify the risk levels where indicated
- document actions taken and results
- provide summary report of findings by subject, site, and study levels to study leadership.

13. PUBLICATION OF RESEARCH FINDINGS

Publication of the results of this trial will be governed by the policies and procedures developed by the Steering Committee, and the PPAS Committee. Both Committees will have NIH scientists as members. Any abstract, manuscript or major presentation will be made available for review by NIH, in accordance with NIH policies, prior to submission. Per NIH policies, a public data set that is not identifiable will become available when the study has been completed.

14. REFERENCES

1. Clarke CN, Clarke NE, Mosher RE. Treatment of angina pectoris with disodium ethylene diamine tetraacetic acid. Am J Med Sci. 1956;232(6):654-66. PubMed PMID: 13372537.
2. Kitchell JR, Meltzer LE, Seven MJ. Potential uses of chelation methods in the treatment of cardiovascular diseases. Prog Cardiovasc Dis. 1961;3:338-49. PubMed PMID: 13756462.
3. Kitchell JR, Palmon F, Jr., Aytan N, Meltzer LE. The treatment of coronary artery disease with disodium EDTA. A reappraisal. Am J Cardiol. 1963;11:501-6. PubMed PMID: 14033183.
4. Lamar CP. Chelation Therapy of Occlusive Arteriosclerosis in Diabetic Patients. Angiology. 1964;15:379-95. PubMed PMID: 14210345.
5. Olszewer E, Carter JP. EDTA chelation therapy in chronic degenerative disease. Med Hypotheses. 1988;27(1):41-9. PubMed PMID: 3144646.
6. Casdorph HR, Farr CH. EDTA chelation therapy III: treatment of peripheral arterial occlusion, an alternative to amputation. J Holistic Med. 1981;3:101-17.
7. Guldager B, Jelnes R, Jorgensen SJ, Nielsen JS, Klaerke A, Mogensen K, Larsen KE, Reimer E, Holm J, Ottesen S. EDTA treatment of intermittent claudication--a double-blind, placebo-controlled study. J Intern Med. 1992;231(3):261-7. PubMed PMID: 1556523.
8. van Rij AM, Solomon C, Packer SG, Hopkins WG. Chelation therapy for intermittent claudication. A double-blind, randomized, controlled trial. Circulation. 1994;90(3):1194-9. PubMed PMID: 8087928.
9. Knudtson ML, Wyse DG, Galbraith PD, Brant R, Hildebrand K, Paterson D,

Richardson D, Burkart C, Burgess E, Program to Assess Alternative Treatment Strategies to Achieve Cardiac Health I. Chelation therapy for ischemic heart disease: a randomized controlled trial. JAMA. 2002;287(4):481-6. PubMed PMID: 11798370.
10. Lamas GA, Ackermann A. Clinical evaluation of chelation therapy: is there any wheat amidst the chaff? Am Heart J. 2000;140(1):4-5. doi: 10.1067/mhj.2000.107549. PubMed PMID: 10874253.
11. Lewin MR. Chelation therapy for cardiovascular disease. Review and commentary. Tex Heart Inst J. 1997;24(2):81-9.
12. Lamas GA, Goertz C, Boineau R, Mark DB, Rozema T, Nahin RL, Drisko JA, Lee KL. Design of the Trial to Assess Chelation Therapy (TACT). Am Heart J. 2012;163(1):7–12.
13. Lamas GA, Goertz C, Boineau R, Mark DB, Rozema T, Nahin RL, Lindblad L, Lewis EF, Drisko J, Lee KL, Investigators T. Effect of disodium EDTA chelation regimen on cardiovascular events in patients with previous myocardial infarction: the TACT randomized trial. JAMA. 2013;309(12):1241-50. doi: 10.1001/jama.2013.2107. PubMed PMID: 23532240; PMCID: 4066975.
14. Escolar E, Lamas GA, Mark DB, Boineau R, Goertz C, Rosenberg Y, Nahin RL, Ouyang P, Rozema T, Magaziner A, Nahas R, Lewis EF, Lindblad L, Lee KL. The effect of an EDTA-based chelation regimen on patients with diabetes mellitus and prior myocardial infarction in the Trial to Assess Chelation Therapy (TACT). Circ Cardiovasc Qual Outcomes. 2014;7(1):15-24. doi: 10.1161/CIRCOUTCOMES.113.000663. PubMed PMID: 24254885; PMCID: 4111470.
15. Lamas GA, Boineau R, Goertz C, Mark DB, Rosenberg Y, Stylianou M, Rozema T, Nahin RL, Terry Chappell L, Lindblad L, Lewis EF, Drisko J, Lee KL. EDTA chelation therapy alone and in combination with oral high-dose multivitamins and minerals for coronary disease: The factorial group results of the Trial to Assess Chelation Therapy. Am Heart J. 2014;168(1):37-44 e5. doi: 10.1016/j.ahj.2014.02.012. PubMed PMID: 24952858; PMCID: 4069605.
16. Navas-Acien A, Guallar E, Silbergeld EK, Rothenberg SJ. Lead exposure and cardiovascular disease--a systematic review. Environ Health Perspect. 2007;115(3):472-82. doi: 10.1289/ehp.9785. PubMed PMID: 17431501; PMCID: 1849948.
17. Menke A, Muntner P, Batuman V, Silbergeld EK, Guallar E. Blood lead below 0.48 micromol/L (10 microg/dL) and mortality among US adults. Circulation. 2006;114(13):1388-94. doi: 10.1161/CIRCULATIONAHA.106.628321. PubMed PMID: 16982939.
18. Tellez-Plaza M, Guallar E, Howard BV, Umans JG, Francesconi KA, Goessler W, Silbergeld EK, Devereux RB, Navas-Acien A. Cadmium exposure and incident cardiovascular disease. Epidemiology. 2013;24(3):421-9. doi: 10.1097/EDE.0b013e31828b0631. PubMed PMID: 23514838; PMCID: 4142588.
19. Tellez-Plaza M, Jones MR, Dominguez-Lucas A, Guallar E, Navas-Acien A. Cadmium exposure and clinical cardiovascular disease: a systematic review. Curr Atheroscler Rep. 2013;15(10):356. doi: 10.1007/s11883-013-0356-2. PubMed PMID: 23955722; PMCID: 3858820.

20. Tellez-Plaza M, Navas-Acien A, Menke A, Crainiceanu CM, Pastor-Barriuso R, Guallar E. Cadmium exposure and all-cause and cardiovascular mortality in the U.S. general population. Environ Health Perspect. 2012;120(7):1017-22. doi: 10.1289/ehp.1104352. PubMed PMID: 22472185; PMCID: 3404657.
21. American Diabetes Association. Diabetes Fast Facts. 2014. Professional.diabetes.org/facts. Last accessed on December 1, 2015.
22. American Diabetes Association. Economic costs of diabetes in the US in 2012. Diabetes Care 2013;36:1033 46.http://care.diabetesjournals.org /content/early/2013/03/05/dc12-2625.
23. Solenkova NV, Newman JD, Berger JS, Thurston G, Hochman JS, Lamas GA. Metal pollutants and cardiovascular disease: mechanisms and consequences of exposure. Am Heart J. 2014;168(6):812-22. doi: 10.1016/j.ahj.2014.07.007. PubMed PMID: 25458643; PMCID: 4254412.
24. Cosselman KE, Navas-Acien A, Kaufman JD. Environmental factors in cardiovascular disease. Nat Rev Cardiol. 2015;12(11):627-42. doi: 10.1038/nrcardio.2015.152. PubMed PMID: 26461967.
25. Arenas I, Navas-Acien A, Lamas GA. Enhanced Vasculotoxic Metal Excretion in Post-Myocardial Infarction Patients Receiving Edetate Disodium-Based Infusion. J Am Coll Cardiol. 2016;67(13_S):2125-2125 (abstract).
26. Waters RS, Bryden NA, Patterson KY, Veillon C, Anderson RA. EDTA chelation effects on urinary losses of cadmium, calcium, chromium, cobalt, copper, lead, magnesium, and zinc. Biol Trace Elem Res. 2001;83(3):207-21. doi: 10.1385/BTER:83:3:207. PubMed PMID: 11794513.
27. Foreman H, Trujillo TT. The metabolism of C14 labeled ethylenediaminetetraacetic acid in human beings. J Lab Clin Med. 1954;43(4):566-71. PubMed PMID: 13163555.
28. Popovici A, Geschickter CF, Reinovsky A, Rubin M. Experimental control of serum calcium levels in vivo. Proc Soc Exp Biol Med. 1950;74(2):415-7. PubMed PMID: 15440839.
29. Perry HM, Jr., Perry EF. Normal concentrations of some trace metals in human urine: changes produced by ethylenediaminetetraacetate. J Clin Invest. 1959;38(8):1452-63. doi: 10.1172/JCI103922. PubMed PMID: 13673103; PMCID: 442101.
30. Guldager B, Jorgensen PJ, Grandjean P. Metal excretion and magnesium retention in subjectsubjects with intermittent claudication treated with intravenous disodium EDTA. Clin Chem. 1996;42(12):1938-42. PubMed PMID: 8969629.
31. Spencer H, Vankinscott V, Lewin I, Laszlo D. Removal of calcium in man by ethylenediamine tetra-acetic acid. A metabolic study. J Clin Invest. 1952;31(12):1023-7. doi: 10.1172/JCI102694. PubMed PMID: 13011191; PMCID: 436507.
32. Bearn AG, Kunkel HG. Abnormalities of copper metabolism in Wilson's disease and their relationship to the aminoaciduria. J Clin Invest. 1954;33(3):400-9. doi: 10.1172/JCI102912. PubMed PMID: 13143088; PMCID: 1072519.
33. http://www.fda.gov/Drugs/DrugSafety/PostmarketDrugSafetyInformationforPatientsandProviders/ucm113738.htm. Last accessed May 7, 2016.
34. Abstract 10466: Post myocardial Infarction Treatment with Edetate Disodium

was Safe in the Trial to Assess Chelation Therapy. Jeanne Drisko, Karen P Alexander, Rhonda S Roberts, L. Terry Chappell, Kerry L Lee, Robin Boineau, Daniel B Mark, Richard L Nahin, Christine Goertz, Yves Rosenberg, Gervasio A Lamas. Circulation. 2015;132:A10466.
35. Thygesen K, Alpert JS, Jaffe AS, Simoons ML, Chaitman BR, White HD: the Writing Group on behalfof the Joint ESCA/ACCF/AHA/WHF Task Force for the Universal Definition of Myocardial Infarction. . Third universal definition of myocardial infarction. Circulation. 2012;126(16):2020-35. doi: 10.1161/CIR.0b013e31826e1058. PubMed PMID: 22923432.
36. US Census Bureau Population Estimates. Historical Data: 2000s. US Census Bureau Web Site. Available at: http://www.census.gov/poptest/data/historical/2000s/index.html. Accessed January 27, 2015.
37. Weitzman S, Wang C, Rosamond WD, et al. Is diabetes an independent risk factor for mortality after myocardial infarction? The ARIC (Atherosclerosis Risk in Communities) Surveillance Study. *Acta Diabetol*. 2004;41(2). doi:10.1007/s00592-004-0148-9.
38. Go AS, Mozaffarian D, Roger VL, et al. Heart disease and stroke statistics--2014 update: a report from the American Heart Association. *Circulation*.
39. Blackwell DL, Lucas JW, Clarke TC. Summary health statistics for U.S. adults: national health interview survey, 2012. *Vital Health Stat 10*. 2014;(260):1–161.
40. Gore MO, Patel MJ, Kosiborod M, Parsons LS, Khera A, de Lemos JA, Rogers WJ, Peterson ED, Canto JC, McGuire DK, National Registry of Myocardial Infarction I. Diabetes mellitus and trends in hospital survival after myocardial infarction, 1994 to 2006: data from the national registry of myocardial infarction. Circ Cardiovasc Qual Outcomes. 2012;5(6):791-7. doi:
41. Farkouh ME, Domanski M, Sleeper LA, Siami FS, Dangas G, Mack M, Yang M, Cohen DJ, Rosenberg Y, Solomon SD, Desai AS, Gersh BJ, Magnuson EA, Lansky A, Boineau R, Weinberger J, Ramanathan K, Sousa JE, Rankin J, Bhargava B, Buse J, Hueb W, Smith CR, Muratov V, Bansilal S, King S, 3rd, Bertrand M, Fuster V, Investigators FT. Strategies for multivessel revascularization in patients with diabetes. N Engl J Med. 2012;367(25):2375-84. doi: 10.1056/NEJMoa1211585. PubMed PMID: 23121323.
42. Hausmann LRM, Gao S, Mor MK, Schaefer JH, Fine MJ. Understanding racial and ethnic differences in patient experiences with outpatient health care in Veterans Affairs Medical Centers. *Med Care*. 2013;51(6):532–539. doi:10.1097/MLR.0b013e318287d6e5.
43. Shaw LJ, Shaw RE, Merz CNB, et al. Impact of ethnicity and gender differences on angiographic coronary artery disease prevalence and in-hospital mortality in the American College of Cardiology-National Cardiovascular Data Registry. *Circulation*. 2008;117(14):1787–1801. doi:10.1161/CIRCULATIONAHA.107.726562.
44. Devine, BJ. Gentamicin therapy. Drug Intell Clin Pharm. 1974; 8 (11): 650–5. doi:10.1177/106002807400801104
45. Payne RB, Little AJ, Williams RB, Milner JR. Interpretation of serum calcium in patients with abnormal serum proteins. Br Med J. 1973;4(5893):643-6. PMID:

4758544; PMCID: 1587636
46. Rozema T. Special Issue: Protocols for Chelation Therapy. J Adv Med. 1997;(10):5–90.
47. Documentation of the Threshold Limit Values for Chemical Substances and Physical Agents and Biological Exposure Indices. American Conference of Governmental Industrial Hygienists 2004. Cincinnati, OH.
48. Freidlin B, Korn EL, Gray R. A general inefficacy interim monitoring rule for randomized clinical trials. Clin Trials. 2010;7(3):197-208. doi: 10.1177/1740774510369019. PubMed PMID: 20423925
49. Haybittle JL. Repeated assessment of results in clinical trials of cancer treatment. Br J Radiol 1971;44:793-7.
50. Peto R, Pike MC, Armitage P, Breslow NE, Cox DR, Howard SV, Mantel N, McPherson K, Peto J, Smith PG. Design and analysis of randomized clinical trials requiring prolonged observation of each patient. I. Introduction and design. Br J Cancer 1976;34:585-612.
51. Kaplan EL, Meier P. Nonparametric estimation from incomplete observations. J Am Stat Assoc. 1958; 53:457-481.
52. Anderson PK, Gill RD. Cox's regression model for counting processes: a large sample study. Ann Stat 1982;10:1100-1120.
53. Lin DY, Wei LJ. The robust inference for the Cox proportional hazards model. J Am Stat Assoc 1989;84:1074-1078.
54. Wei LJ, Lin DY, Weissfeld L. Regression analysis of multivariate incomplete failure time data by modeling marginal distributions. J Am Stat Assoc 1989;84:1064-1072.
55. Bang H, Tsiatis AA. Estimating Medical Costs with censored data. Biometrika. 2000; 87 (2): 329-343.

Statistical Analysis Plan

Protocol Version:	Amendment 3
Protocol Date:	November 1, 2019
Analysis Plan Version:	2.0
Analysis Plan Date:	September 12, 2023

TABLE OF CONTENTS

1 Introduction 6
1.1 Study Design 6
1.2 Study Objectives 6
1.2.1 Primary Objective 6
1.2.2 Secondary Objectives 6
1.2 Sample size Considerations 7
2 General Considerations for Data Analyses 9
2.1 Analysis Populations 9
2.1.1 Intention-to-treat Population (ITT) 9
2.1.2 Modified Intention-to-treat Infusion Population (mITT) 9
2.2 Data Sources 9
2.3 General Analysis Conventions 9
2.4 Disposition of Participants 10
2.5 Demographic and Baseline Characteristics 10
2.6 Handling of Missing Data 10
3 Analysis of Clinical Endpoints 11
3.1 General 11
3.1.1 Estimands and Censoring Scheme 11
3.1.2 Event Data 16
3.1.3 Assessment of Model Assumptions 16
3.1.4 Multiple Comparisons and Composite Endpoints 17
3.2 Primary Endpoint Analysis 17
3.2.1 Primary Analysis 17
3.2.2 Sensitivity Analyses of the Primary Outcome 18
3.3 Analysis of the Secondary Endpoints 19
3.3.1 Recurrent-event analysis of the primary endpoint 19
3.3.2 Time to Event Analysis of the Secondary Endpoints 20
3.3.3 Sensitivity Analyses of the Secondary Outcomes 21
3.4 Subgroup Analyses 21
3.5 Safety Analyses 22
4 References 23
5 SAP Revision 24

6 Appendices 25

Signature page

Prepared by:

Hayley Nemeth

Electronically signed by: Hayley Nemeth
Reason: Approved
Date: Sep 19, 2023 13:06 EDT

………………………………………… ……………………………………

Hayley Nemeth, MS
DCRI Statistics Representative
Biostatistician III
Duke Clinical Research Institute

Date

Reviewed by:

Kevin J. Anstrom

Electronically signed by: Kevin J. Anstrom
Reason: Approved
Date: Sep 19, 2023 18:29 EDT

………………………………………… ……………………………………

Kevin J. Anstrom, PhD
Co-Principal Investigator, DCC
University of North Carolina Gillings
School of Global Public Health

Date

Approved by:

Gervasio Lamas

Electronically signed by: Gervasio Lamas
Reason: Approved
Date: Sep 21, 2023 16:42 EDT

………………………………………… ……………………………………

Gervasio A. Lamas, MD,
Principal Investigator, CCC
Mount Sinai Medical Center and Columbia
University Medical Center

Date

Daniel Mark

Electronically signed by: Daniel Mark
Reason: Approved
Date: Sep 23, 2023 18:08 EDT

………………………………………… ……………………………………

Daniel B. Mark, MD, MPH,
Co-Principal Investigator, DCC
Duke Clinical Research Institute

Date

Mario Stylianou

Electronically signed by: Mario Stylianou
Reason: Approved
Date: Sep 25, 2023 12:07 EDT

………………………………………… ……………………………………

Mario Stylianou, PhD
NIH Representative
National Heart, Lung, and Blood
Institute/National Institutes of Health

Date

Abbreviations

Abbreviation	Definition
CEC	Clinical Events Committee
CI	Confidence Interval
CONSORT	Consolidated Standards of Reporting Trials
CRF	Case Report Form
CSR	Clinical Study Report
DCRI	Duke Clinical Research Institute
EDC	Electronic Data Capture
FU	Follow-Up
HR	Hazard Ratio
ITT	Intention-To-Treat
MI	Myocardial Infarction
mITT	Modified Intention-To-Treat
NDI	National Death Index
OMVM	Oral MultiVitamins and Multiminerals
SAE	Serious Adverse Event
SAP	Statistical Analysis Plan
TACT2	Trial to Assess Chelation Therapy 2
WLW	Wei, Lin, and Weissfeld

1 Introduction

The purpose of this Statistical Analysis Plan (SAP) is to describe the analyses and data presentations that will be used for the creation of the primary manuscript of the study which will focus on the comparison of active chelation vs. placebo chelation.

1.1 Study Design

The Trial to Assess Chelation Therapy 2 (TACT2) is a randomized, double blinded, placebo-controlled 2x2 factorial clinical trial. The study treatments are edetate disodium-based chelation (weekly infusion for 40 weeks) and high-dose oral multivitamins and multi-minerals (OMVM) (twice daily for up to 5 years). Subjects with diabetes mellitus with a prior myocardial infarction (MI) are recruited from participating sites in the US and Canada and randomized in a 1:1:1:1 fashion to 4 groups:

i. Active chelation + active OMVM
ii. Active chelation + placebo OMVM
iii. Placebo chelation + active OMVM
iv. Placebo chelation + placebo OMVM.

Patients will be followed for clinical events until the earlier of database lock or five years from randomization. Clinical outcomes will be collected by study sites at each infusion visit and by the DCRI Call Center at month 6 and 12, and every 4 months afterwards with the last assessment at month 60.

TACT2 is an event driven trial with a plan to continue until at least 282 primary endpoint events are identified. The original planned sample size was 1200 participants. The sample size was later modified to 1100. The final enrollment is 1000, but with an increased follow-up time to preserve statistical power. More details on sample size and power calculation are included later in the SAP.

1.2 Study Objectives

TACT2 seeks to replicate TACT, which found a reduction of recurrent cardiovascular events most prominent in the subgroup of post-MI diabetic patients receiving edetate disodium-based chelation therapy.

1.2.1 Primary Objective

The primary objective of TACT2 is to determine if the chelation-based strategy increases the time to the first occurrence of any of the components of the primary composite endpoint of all-cause mortality, MI, stroke, coronary revascularization, or hospitalization for unstable angina compared with the placebo chelation strategy.

1.2.2 Secondary Objectives

The secondary objectives of TACT2 are to determine:

i. if the chelation-based strategy reduces the overall rate of occurrence of the events which define the primary composite endpoint of all-cause mortality, MI, stroke, coronary revascularization, or hospitalization for unstable angina events compared with the placebo chelation strategy.

ii. to determine if the chelation-based strategy increases the time to the first occurrence of a secondary composite endpoint of cardiovascular mortality, MI, or stroke compared with the placebo chelation strategy.

iii. to determine if the chelation-based strategy increases the time to all-cause mortality compared with the placebo chelation strategy.

1.2 Sample size Considerations

The study was originally designed to enroll 1200 participants with a minimum follow-up of 12 months. Since the enrollment was slower than projected, the DSMB conducted a blinded review of enrollment aggregate event rates, and projected event rates in July 2019. The study team proposed a new sample size based on the updated projected event rate, an assumed hazard ratio of 0.7 comparing chelation and placebo, and a dropout rate of 5% (Table 1). While the sample size was reduced to 1100 with an extended enrollment time by December 2020, the follow-up (FU) period was extended to 2 years to ensure to have the targeted power of 85%.

The final study enrollment was 1000. The study team evaluated the study power with a newly extended FU period of 2.5 years assuming various dropout rates (Table 2). Even with a dropout rate of 10%, the study power exceeds the targeted power of 85% with 1000 participants and 2.5 years of follow-up.

The full details around sample size adjustment and study power are included in Appendix I.

Table 1. Original and modified sample size and study power

Sample size	Note	Power	Overall event rate (%/pt-yr)	Assumed event rate in EDTA (%/pt-yr)	Assumed event rate in Placebo (%/pt-yr)	Dropout rate	Enrollment year	FU year	Total # events
1200	Original sample size	85%	10.9%	8.97%	12.81%	2%	3	1	282
1100	Modified sample size	88%	9.2%	7.56%	10.82%	5%	4.25	2	308

Table 2. Study power with the final 1000 participants enrolled

Scenario	Overall event rate (%/pt-yr)	Assumed event rate in EDTA (%/pt-yr)	Assumed event rate in Placebo (%/pt-yr)	Overall dropout rate	Mean power	Mean # events
Projected	8.8	7.2	10.4	5%	89%	299
				7%	88%	295
				10%	87%	288

2 General Considerations for Data Analyses

2.1 Analysis Populations

2.1.1 Intention-to-treat Population (ITT)

The ITT population will include all randomized participants, including those who are later found not to meet study eligibility criteria. There were two participants who mistakenly "re-joined" the trial after infusions ended and were re-randomized in a second site, near their original site. The detail for the two participants is in Appendix II. They will only be counted once in the ITT population. Their data from the 2nd randomization will not be included the analysis.

2.1.2 Modified Intention-to-treat Infusion Population (mITT)

All participants in the ITT population who have completed at least one infusion will be included in the modified intention-to-treat population. The mITT population will be the primary analysis population.

2.2 Data Sources

The data used for analysis will come from 5 sources: the randomization data from the Interactive Voice/Web System (IXRS), the e-CRF data collected by the iMedidata Rave electronic data capture system (RAVE), the adjudicated endpoint data from the clinical events committee database (CEC), the site-level information stored in the Clinical Trial Management System (CTMS) database, and the CDC National Death Index (NDI).

Masked-source NDI search results will be used to focus a search for supporting clinical and other documentation. If supporting documentation is obtained, the data will be submitted to the CEC process prior to being used in the analysis. If needed, a Mortality Review Committee will be assigned to classify potential death events when there are insufficient/incomplete or contradictory NDI and/or Call Center data to make a reliable determination. Additional details will be described in a separate charter.

2.3 General Analysis Conventions

Statistical comparisons will be performed using two-sided significance tests. An alpha level of 0.05 will determine significance unless otherwise specified.

Continuous variables will be presented in terms of percentiles (e.g., median, 25th percentile, and 75th percentiles) along with means and standard deviations. Categorical variables will be summarized in terms of frequencies and percentages.

All programs written to create analysis datasets or perform analyses will be validated according to SOPs by the Statistical Programming group.

All statistical analysis will be performed using SAS® v9.4 or higher (Cary, NC USA) and/or other proper software, e. g. R.

2.4 Disposition of Participants

Disposition of participants (number randomized, number who received treatment, number of infusions received per patient, number of withdrawals and dropouts from the study, number lost to follow-up, length of follow-up, time since last contact, number unmasked) will be summarized by active chelation vs placebo chelation groups using a CONSORT flow diagram and subject disposition table.

Lost to follow-up among alive and non-withdrawn participants will be reported in the CONSORT diagram as described above. The number of participants lost to follow-up with a primary endpoint will be included in the total and also reported separately. Participants alive and not withdrawn or lost to follow-up will be defined as completing the study.

The definition for lost to follow-up will be:

No contact within 12 months of the end of study. End of study is defined as the 5-year informed consent expiration date or administrative end of study date (June 30, 2023). Last contact is defined as the last known alive date.

2.5 Demographic and Baseline Characteristics

Demographic and baseline characteristics will be summarized by active and placebo chelation groups and for the overall mITT population. These include cardiovascular risk factors, comorbidities, relevant descriptors from the history, concomitant medical care including post-MI medications, treatments for diabetes, over-the-counter supplements and herbal remedies. Physical examination, laboratory data, cardiopulmonary symptoms, and past clinical events. Blood and urine metals pre- and post-chelation are discussed in the SAP for the Trave Metals Biorepository Core Lab.

2.6 Handling of Missing Data

Every effort will be made to obtain complete data during the course of the trial. If missing data remains despite all the efforts, rules on how to handle the missing data will be implemented.

If the proportion of subjects with missing values in any of the covariates for the primary analysis, i.e. age, sex, and baseline insulin use, is greater than 1%, then the missing values will be imputed to the mode.

Since the clinical endpoints are adjudicated by the CEC, we don't anticipate missing values for positively adjudicated outcomes. In the very rare occasion where the event day is missing, it will be imputed as the last day of the month. If missing day and month, then December 31st of the provided year will be used. If the year is missing, no imputation will be performed.

3 Analysis of Clinical Endpoints

3.1 General

3.1.1 Estimands and Censoring Scheme

Summaries of the descriptions of the estimand attributes and censoring schemes are shown in **Table 3**. Like TACT, the primary analysis will include all identified events obtained as part of the planned follow-up from infusion visits and call center interviews.

Table 3. Key Study Estimands and Censoring Scheme.

Objective Description / Study Population	Endpoint	Summary statistics	Events to be included in the endpoint derivation	Censoring date
Primary Objective/ mITT	Time from randomization to the first occurrence of primary composite endpoint of all-cause mortality, MI, stroke, coronary revascularization, or hospitalization for unstable angina	Hazard ratio estimate based on a Cox proportional hazards model.	All positively adjudicated primary endpoint events post-randomization except those occurring after withdrawal of consent or ICF expiration.<br><br>Events occurring after withdrawal consent or ICF expiration will be censored. Subjects with events after withdrawal or ICF expiration will be censored at the min(date withdrew consent, date of last contact with patient/proxy where event status was assessed).	Among subjects not experiencing endpoint of interest:<br>Censoring date = min(date withdraw consent, date of last contact with patient/proxy where event status was assessed)<br><br>Date of last contact defined as:<br>If the last contact occurred during the study infusion phase - use last infusion visit date where survival status and event status were assessed (yes or no to event).<br><br>If last contact occurred during call center follow up period – use 'complete'/'incomplete' status date by patient/proxy where survival status and hospitalization status were assessed (yes or no to hospitalization).

				For composite endpoints, date of last assessment = the earliest of the last assessment date among all components.
Secondary Objective / mITT	Overall rate of recurrent events due to all-cause mortality, MI, stroke, coronary revascularization, or hospitalization for unstable angina	Hazard ratio estimate based on a proportional intensity model by Andersen-Gill and a marginal model by Wei, Lin and Weissfeld.	*same as primary endpoint	*same as primary endpoint
Secondary Objective / mITT	Time from randomization to the first occurrence of the secondary composite endpoint of cardiovascular mortality, MI, or stroke	Cause specific hazard ratio estimate based on a cause-specific Cox proportional hazards model.	All positively adjudicated endpoint events post-randomization except those occurring after withdrawal of consent or ICF expiration date.<br><br>Non-CV death or undetermined cause of death is considered a competing risk.<br><br>Among subjects experiencing competing risk (non-CV death or undetermined death is a competing risk of CV death or MI/stroke if a subject died without having any	Among subjects not experiencing endpoint of interest or competing event:<br>Censoring date = min(date withdraw consent, date of last contact with patient/proxy where event status was assessed)<br><br>*Date of last contact defined in 1st row, 5th column (primary endpoint)

			of these events beforehand):<br>Event date = non-CV or undetermined death date;	
Secondary Objective / mITT	Time from randomization to all-cause mortality	Hazard ratio estimate based on a Cox proportional hazards model.	All deaths post-randomization (including adjudicated deaths obtained from the NDI search)<br><br>A death date after withdrawn consent or ICF expiration (obtained from public data source) is considered an event.	Among subjects who are alive:<br>Censoring date = (date last known alive)<br><br>Date of last known alive defined as:<br>If the last contact occurred during the study infusion phase - use last infusion visit date.<br><br>If last contact occurred during call center follow up period – use 'complete'/'incomplete' status date by patient/proxy **or ('not done' status date where source = patient)** or last alive date from Call Center where death status was assessed.
Secondary Objective / mITT	Total number of SAE and percentage of patients experiencing at least 1 SAE.	Number and proportions by treatment group. Confidence intervals will be based on the Miettinen-Nurminen method.	Randomization to min(withdraw consent, 30 days post final infusion)	N/A

Sensitivity Analysis of the Primary Outcome / Bayesian analysis using TACT results as one of the priors / mITT	*Same as primary analysis	Treatment effect mean posterior hazard ratio based on a Bayesian time to event model	*Same as primary analysis	*Same as primary analysis
Sensitivity Analysis of the Secondary All-Cause Mortality Outcome / Bayesian analysis using TACT results as one of the priors / mITT	*Same as primary analysis	Treatment effect mean posterior hazard ratio based on a Bayesian time to event model	*Same as primary analysis	*Same as primary analysis
Sensitivity Analysis of the Primary Outcome / **ITT**	*Same as primary analysis	*Same as primary analysis	*Same as primary analysis	*Same as primary analysis
Sensitivity Analysis of the Primary Outcome / mITT with imputed event status for subjects withdrew /lost to follow up	*Same as primary analysis	*Same as primary analysis	*Same as primary analysis except that the event status will be imputed for subjects who withdrew consent or lost to follow up before study completion	*Same as primary analysis

Sensitivity Analysis of the Primary Outcome/ mITT with the exclusion of positively adjudicated events > 1 year after the last contact where all components of the composite were assessed	*Same as primary analysis	*Same as primary analysis	*Same as primary analysis but exclude positively adjudicated events more than 1 year from the most recent study contact where all components of the composite endpoint were assessed.	Among subjects not experiencing endpoint of interest or experiencing first CEC event > 1 year after event assessment date: Censoring date = min(date withdraw consent, date of last contact with patient/proxy where event status was assessed)<br><br>*Date of last contact defined in 1st row, 5th column (primary endpoint)
Sensitivity Analysis of the Secondary Composite Outcome/ Where undetermined death is considered CV-death / mITT	*same as secondary composite endpoint	*same as secondary composite endpoint, where:<br><br>Undetermined death is analyzed as CV-death.	*same as secondary composite endpoint	*same as secondary composite endpoint
Sensitivity Analysis of the Secondary Composite Objective / Cause of death based on best available data (from CEC or CRF if CEC is unavailable) / mITT	*Same as secondary composite analysis	*Same as secondary composite analysis	*Same as secondary composite analysis, where:<br><br>Cause of death is determined based on best available data. If cause of death from CEC is available, use CEC cause of death. Otherwise, if CRF cause of death available, use CRF cause of death.	*Same as secondary composite analysis

			If cause of death is unavailable in CEC and CRF, use NDI cause of death.	
Sensitivity Analysis of the Secondary All-Cause Mortality Outcome / Censor at the date of NDI assessment / mITT	*Same as secondary all-cause mortality analysis	*Same as secondary all-cause mortality analysis	*Same as secondary all-cause mortality analysis	Among subjects who are alive:<br>Censoring date = max(date of NDI assessment, last known alive date)<br><br>Date of NDI assessment is defined as:<br>The last date that NDI results were assessed. This will be consistent for all participants without an event.

*See section 2.1 for the definition of ITT and mITT.

One participant who was randomized twice and received infusions from the second site will be censored at the second randomization. The other participant who was lost to follow-up at the first site and then was randomized at the second site will be censored at the time of lost to follow-up at the first site.

3.1.2 Event Data

Analyses of the clinical endpoints will use the CEC-adjudicated data for death, MI, stroke, coronary revascularization, or hospitalization for unstable angina.

3.1.3 Assessment of Model Assumptions

The validity of the proportional hazards assumption will be examined using a standard graphical method such as Schoenfeld residual plots. If the assumption holds, the survival curves should be approximately parallel to each other. An additional analytical method that includes the supremum test as implemented by using the ASSESS statement in PROC PHREG in SAS version 9.4 may be utilized. A *P*-value of < 0.05 indicates violation of the proportional hazards assumption. If there is evidence of non-proportionality, a time dependent covariate will be included in the model, or a restricted mean survival time model will be performed.

3.1.4 Multiple Comparisons and Composite Endpoints

With the number of primary hypotheses and the various secondary endpoints to be analyzed and compared, there is an increased probability that at least one of the comparisons could be "significant" by chance. We have pre-specified the primary and secondary outcome variables to help avoid over-interpretation and to reduce the problems inherent with multiple testing. We will be conservative in the interpretation of these analyses, taking into account the degree of significance, and looking for consistency across endpoints.

3.2 Primary Endpoint Analysis

The primary endpoint is the composite of all-cause mortality, MI, stroke, coronary revascularization, or hospitalization for unstable angina.

If two of the component events of the primary composite endpoint occur on the same day, the following hierarchy will be used when reporting the components of the primary endpoint. For example, a patient with stroke and all-cause mortality on the same date will be considered as having stroke when summarizing individual events within the primary composite outcome. This hierarchy will only be used for summarizing events within the primary composite outcome and will not affect the primary analysis.

i. MI
ii. Stroke
iii. Hospitalization for unstable angina
iv. Coronary revascularization
v. All-cause mortality

3.2.1 Primary Analysis

The primary objective is to determine if the chelation-based strategy increases the time to composite endpoint compared with the placebo chelation strategy. The primary composite endpoint will be analyzed using a Cox proportional hazards regression model of time from randomization to the first occurrence of all-cause mortality, MI, stroke, coronary revascularization, or hospitalization for unstable angina.

The primary analysis will be based on a Cox model which includes indicator variables for the active chelation as the primary variable of interest and active OMVM groups, age, sex, and baseline insulin use as covariates. Age will be included in the model in the form of cubic splines using three knots at 10th, 50th, and 90th percentiles. Two separate manuscripts will explore the effect of active OMVM and the effects of chelation by OMVM.

The chelation treatment effect will be summarized using hazard ratios and associated 95% CIs. A hazard ratio of < 1 would suggest a benefit for the chelation-based therapy. Kaplan Meier survival estimates will be constructed based on the time from randomization to the first

primary event occurrence. Events occurring after withdrawal of consent or informed consent expiration will be censored.

The OMVM treatment effect from the Cox model will be estimated using the Cox model described above. A hazard ratio of < 1 would suggest a benefit for OMVM treatment. Given the focus on chelation therapy, the interpretation of the OMVM treatment effect will be considered exploratory. A full set of analyses of the 2x2 design will be reported in a separate manuscript.

Four interim analyses were conducted at 25%, 40%, 60%, and 80% of expected information using a Haybittle-Peto-type boundary for efficacy (i.e. p<=0.001, Z=3). For this reason, the adjusted two-sided significance level for the final analysis is 0.05 (i.e., the corresponding critical value, Z = -1.96).

Formal statistical hypothesis testing will follow a hierarchy based on statistical significance of the prior endpoint. If the prior endpoint is statistically significant at the 0.05 level, the next endpoint's p-value will be tested for significance. The outcome hierarchy will be evaluated as follows:

1) Primary composite outcome
2) Secondary all-cause mortality outcome (with conservative censoring)
3) Secondary composite outcome of cardiovascular mortality, MI, or stroke
4) Secondary recurrent event analysis (Andersen & Gill method)

3.2.2 Sensitivity Analyses of the Primary Outcome

The primary hypothesis for the primary composite endpoint will be tested using the ITT population as a sensitivity analysis.

A sensitivity analysis was initially planned to investigate the potential effect of the COVID-19 outbreak on the primary analysis. Instead, the potential effect of COVID-19 may be explored in a separate manuscript.

A sensitivity analysis will use Bayesian methods to estimate the primary outcome treatment effect posterior distribution using the first TACT trial results and other related information to develop a set of priors for the treatment effect (Zampieri, 2020). As suggested by Zampieri, these analyses will include at least one prior distribution for the effect of chelation vs. placebo in each of the following categories: skeptical, pessimistic, and optimistic. The priors will be assumed to follow a normal distribution and will represent the log hazard ratio from the Cox model. There will be two skeptical (non-informative) priors for the log hazard ratio with a mean of zero and differing standard deviations (e.g., one standard deviation will be 10 and the other will be 100). Two pessimistic priors for the log hazard ratio will also be included, both will be centered around zero and allow for low probability of a favorable treatment effect (e.g., 0.5%

and 2.5% probability of the hazard ratio for chelation of 0.80 compared to placebo). The two optimistic priors will include the overall treatment effect from TACT (e.g., HR of 0.82 with a 95% CI of 0.69 to 0.99) and diabetes subgroup treatment effect from TACT (e.g., HR of 0.59 with a 95% CI of 0.44 to 0.79). In all of the above models, the priors for the parameters associated with other model covariates will be non-informative with large standard deviations (e.g., standard deviation of 100) and center at zero for the associated log hazard ratios. The chelation treatment effect will be summarized using mean posterior hazard ratios and associated 95% highest posterior density credible intervals. A mean posterior hazard ratio of < 1 would suggest a benefit for the chelation-based therapy.

Another sensitivity analysis is planned to assess how the primary analysis would be affected by various assumptions regarding the occurrence of primary endpoint events among those who withdrew consent or were lost to follow-up, as was performed and published in TACT. Baseline characteristics by treatment group will be assessed for the patients who did versus did not withdraw consent or were lost to follow-up. Events will be imputed by simulation only for the consent withdrawal or lost patients who did not have a documented occurrence of one of the primary events prior to the withdrawn consent/lost to follow-up. The different percentages of the withdrawn or lost patients will be assumed to have an event at their censoring time. The event rates among patients that withdrew or were lost to follow-up in each treatment group will be varied across a broad spectrum and include scenarios that were markedly unfavorable to chelation. The imputed event rates for patients who withdrew consent or were lost to follow-up will be combined with the observed event rates of patients who completed the study to assess the treatment effect via a hazard ratio. These analyses will focus on scenarios in which events among withdrawn or lost patients in the active arm are assumed to occur at a higher rate than withdrawn or lost patients in the placebo arm.

Another sensitivity analysis is planned for the primary endpoint to censor, at the point of last contact, those deaths that were identified more than one year following the most recent study contact.

3.3 Analysis of the Secondary Endpoints

The three secondary endpoints are:

i. Recurrent events of the primary composite endpoint
ii. All-cause mortality
iii. Composite of cardiovascular mortality, MI, or stroke

3.3.1 Recurrent-event analysis of the primary endpoint

Recurrent event analyses include all positively adjudicated primary endpoint events attributed to a participant: all-cause mortality, recurrent MI, stroke, coronary revascularization, or hospitalization for unstable angina events. The number of patients with a primary composite endpoint and the frequency of the primary composite endpoint per each component will be

summarized by active chelation and placebo. We analyze this endpoint using two statistical techniques:

i. The generalization of the Cox model to handle recurrent events developed by Andersen & Gill (1982) is the most frequently used approach to estimate treatment differences with total and recurrent events. This approach models gap time (time from enrollment to first event or from one event to the next) and assumes independence of observed event times within the same subject with the adjustment of the covariates. Our modeling will use robust standard errors to account for individual patients' heterogeneity and covariance across event times. The treatment effect will be presented as a hazard ratio (EDTA vs. placebo) and associated 95% CIs and *P*-value. It will be the primary analysis for the total number of all-cause mortality, recurrent MI, stroke, coronary revascularization, or hospitalization for unstable angina events endpoint.

ii. The stratified marginal Cox modeling approach developed by Wei, Lin, and Weissfeld (WLW) (1989) considers each event recurrence separately and models all the available data for the specific event. Therefore, each subject is considered to be at risk for all events, regardless of how many events each subject actually experienced. A different baseline hazard function is assumed for each event, through use of strata for each event. The WLW will model the marginal hazard of each event's failure time. The time interval for each event for each subject starts at date of randomization and ends at the time of the specific event or censoring. A treatment effect will be presented as a hazard ratio (EDTA vs. placebo) and a 95% CI and *P*-value. This will be considered as a sensitivity analysis.

Both analyses will be adjusted for age, sex, and baseline insulin use. In either analysis, a hazard ratio of < 1 would suggest a benefit for the chelation-based therapy.

An event rate of the endpoint for each treatment group will be calculated as percentage per 100 patient years, accounting for differential follow-up duration. A 95% CI for the event rates will be calculated as

$$\left(\frac{r_C}{T_C}\right) \pm 1.96\sqrt{\frac{r_C}{T_C^2}},$$

where subscript C denotes the chelation arm, and r is the total number of event occurrences, and T is the total follow-up time among all participants.

3.3.2 Time to Event Analysis of the Secondary Endpoints

The analyses for the time to the first occurrence of secondary endpoints (1) time to the first occurrence of the composite of cardiovascular mortality, MI, or stroke; 2) time to all-cause

mortality) will be performed in the same manner as the primary analysis in Section 4.1, replacing the primary composite endpoint with secondary endpoints.

Competing risk of non-CV death or undetermined death will be taken into account for the secondary composite endpoint (time to the first occurrence of the composite of cardiovascular mortality, MI, or stroke). Follow-up will be censored at the time of non-CV or undetermined death if there is no prior composite event. For this endpoint, the treatment effect will be presented as a cause-specific hazard ratio.

All deaths post-randomization will be included in the secondary all-cause mortality endpoint. For this endpoint, a death date after withdrawal of consent or ICF expiration (obtained from public data sources) is considered an event.

3.3.3 Sensitivity Analyses of the Secondary Outcomes

A sensitivity analysis is planned for the secondary composite endpoint where undetermined death will be classified as CV-death.

Another sensitivity analysis is planned for the secondary composite endpoint where cause of death is determined based on the best available data. Cause of death will first be determined based on the data from the CEC. If cause of death from the CEC is unavailable, cause of death will be determined from the CRF data. If cause of death from the CEC and CRF is unavailable, cause of death will be determined based on the NDI data.

A sensitivity analysis is planned for the secondary all-cause mortality endpoint where participants with no event will be censored at the last date that NDI results were assessed. The censoring date will be consistent for all participants without an event.

A sensitivity analysis will use Bayesian methods to estimate the secondary all-cause mortality outcome treatment effect posterior distribution. These analyses will be performed in an analogous manner as the Bayesian analysis of the primary outcome (section 3.2.2).

3.4 Subgroup Analyses

To examine the heterogeneity of the treatment effect, subgroup analyses for the primary and secondary endpoints will be performed using interaction terms within the Cox proportional hazards model. The pre-specified key subgroups of interest are following.

- Sex: female vs. male
- Race: White vs. Black vs. Other
- Ethnicity: Hispanic vs. non-Hispanic (unknown ethnicity is considered missing)
- Age: ≤ 70 years vs. > 70 years
- MI location: anterior MI vs. non-anterior MI

- Pharmacologic treatment of diabetes (two subgroups analyses: no insulin vs. insulin, SGLT2i or GLP-1a vs. neither)
- Known peripheral artery disease at baseline: yes vs. no
- Tertiles of lead (in blood) and cadmium (in urine)

For categorical variables, if the proportional hazards assumption is violated, we will include the subgroup factor as a stratification variable within the Cox regression model. This model structure will decrease the reliance on the proportional hazards assumption. Event rates by treatment and HRs with 95% CIs will be reported for each subgroup. Forest plots will be generated displaying the estimated hazard ratios and 95% CIs for each subgroup. For subgroups defined using continuous variables, the analysis based on the continuous form will be considered primary but for display purposes these variables can also be categorized.

To further analyze the heterogeneity of treatment effects at the patient level, a separate manuscript will use the PATH statement approach for subgroup analyses.

The use of statin treatment at baseline will be evaluated as a subgroup analysis in the OMVM manuscript.

3.5 Safety Analyses

Using the mITT population, the total number of serious adverse events (SAEs) and percentage of participants experiencing at least one SAE from randomization to 30 days post final infusion in each patient group will be compared with confidence intervals based on the Miettinen-Nurminen method. Whether SAEs were associated with treatment and required hospitalization (inpatient hospitalization or prolongation of existing hospitalization) will be summarized per Body System or Organ Class and Dictionary-Driven Term.

4 References

Andersen, P. K., & Gill, R. D. (1982). Cox's regression model for counting processes: a large sample study. *The Annals of Statistics*, 1100-1120.

Finkelstein, D. M., & Schoenfeld, D. A. (1999). Combining mortality and longitudinal measures in clinical trials. *Statistics in Medicine*, 18(11), 1341-1354.

Lamas GA, Anstrom KJ, Navas-Acien A, Boineau R, Kim H, Rosenberg Y, Stylianou M, Jones TLZ, Joubert BR, Santella RM, Escolar E, Aude YW, Fonseca V, Elliott T, Lewis EF, Farkouh ME, Nathan DM, Mon AC, Gosnell L, Newman JD, Mark DB; TACT2 Investigators. The trial to assess chelation therapy 2 (TACT2): Rationale and design. *Am Heart J.* 2022 Oct;252:1-11. doi: 10.1016/j.ahj.2022.05.013. Epub 2022 May 19. PMID: 35598636.

Lamas GA, Goertz C, Boineau R, Mark DB, Rozema T, Nahin RL, Drisko JA, Lee KL. Design of the Trial to Assess Chelation Therapy (TACT). *Am Heart J.* 2012 Jan;163(1):7-12. doi: 10.1016/j.ahj.2011.10.002.

Luo, X., Tian, H., Mohanty, S., & Tsai, W. Y. (2015). An alternative approach to confidence interval estimation for the win ratio statistic. *Biometrics*, 71(1), 139-145.

TACT Final Study SAP. (NCT00044213) https://classic.clinicaltrials.gov/ct2/show/NCT00044213

https://biolincc.nhlbi.nih.gov/studies/tact/#:~:text=Design,regimen%20or%20an%20oral%20placebo.

Wei, L. J., Lin, D. Y., & Weissfeld, L. (1989). Regression analysis of multivariate incomplete failure time data by modeling marginal distributions. *Journal of the American Statistical Association*, 84(408), 1065-1073.

Zampieri FG, Casey JD, Shankar-Hari M; Harrell Jr. FE, and Harhay MO (2021). “Using Bayesian Methods to Augment the Interpretation of Critical Care Trials: An Overview of Theory and Example Reanalysis of the Alveolar Recruitment Trial.” *Am J Respir Crit Care Med*; 203(5): 543-552.

5 SAP Revision

Revision Date MMM dd, yyyy	Section	Summary of Revision	Reason for Revision
SEP 12, 2023	2.2 Data sources, 2.6 Missing data, 3.1.1 Estimands and censoring, 3.2.1 Primary Analysis, 3.2.2, Sensitivity, 3.3.1 Recurrent events, 3.4 Subgroup analyses, Appendix III	Added NDI as a data source. Updated imputation for missing data. Added sensitivity analyses. Added hierarchy for formal hypothesis testing. Added event rate equation for recurrent event analysis. Updated subgroup analyses. Added Bayesian sensitivity analysis.	Additional information on NDI. Missing dates in the data. Additional sensitivity analyses requested. Requests from PIs.

6 Appendices

Appendix I. TACT2 Sample size and study power projections as of March 1, 2021

1. **Enrollment and timeline**
    a. 1000 patients enrolled.

Last rand subject infusion	Last rand subject Follow-up	Last endpoint adjudicated	Database lock
December 2021	June 2023	August 2023	October 2023

2. **Event rate in the TACT diabetic cohort (n=633)**
    a. EDTA: 7.8%/pt-yr = 80 events / 1031 patient-years
    b. Placebo: 13.2%/pt-yr = 117 events / 886 patient-years
    c. Hazard ratio: 0.59

3. **Original and modified sample size and power**

Sample size	Enrollment	FU	Power	Hazard ratio (EDTA/placebo)	Overall event rate (%/pt-yr)	Assumed event rate in EDTA (%/pt-yr)	Assumed event rate in Placebo (%/pt-yr)	Total # events
1200	3 years	1 year	85%	0.7	10.9	8.97	12.81	282
1100	3 years + 15 months	2 years	88%	0.7	9.2	7.56	10.82	308

4. **Statistical power with 1000 enrolled participants as of March 1, 2021**
    a. As of February 23, 2021, 137 patients with at least one adjudicated primary endpoint, 1990 patient-years from 1000 enrolled patients
        i. Proportion of reported events adjudicated as primary endpoints = 69%
        ii. Projected overall event rate: If 39 patients had an adjudicated primary endpoint out of 57 additional patients with at least one reported event, 176/1990 = 8.8%/pt-yr
        iii. Minimum overall event rate: 137/1990 = 6.9%/pt-yr
        iv. Optimistic overall event rate: If 45 patients had an adjudicated primary endpoint out of 57 additional patients with at least one reported event, 182/1990 = 9.1%/pt-yr
    b. Assumptions for simulations with 10K iterations per scenario
        i. No re-consent for those who run out 5 years from the initial consent
        ii. Constant parameters: a two-sided alpha=0.05, hazard ratio of EDTA to placebo=0.7, sample size=1000, FU=2.5 years
        iii. Overall dropout rate: 5, 7, 10%
        iv. Overall event rate: 6.9, 8.8, 9.1%/pt-yr

Scenario	Overall event rate (%/pt-yr)	Assumed event rate in EDTA (%/pt-yr)	Assumed event rate in Placebo (%/pt-yr)	Overall dropout rate	Mean power	Mean # events
Projected	8.8	7.2	10.4	5%	89%	299
				7%	88%	295
				10%	87%	288
Minimum	6.9	5.7	8.1	5%	78%	244
				7%	77%	240
				10%	76%	235
Optimistic	9.1	7.5	10.7	5%	88%	307
				7%	87%	303
				10%	86%	297

Appendix II. Details of two participants who were randomized from two different sites

Double randomization case 1

One participant was randomized again in a neighboring site right after completing 40 infusions from the first site where randomized for the first time. The study team found out the second randomization after the participant finished 35 infusions at the second site. This participant has been actively followed up by the Call Center. This participant will be censored at the time of the second randomization for any analyses using the ITT or mITT populations.

Double randomization case 2

The other participant was lost to follow up after the third infusion at the first site and then randomized again in a neighboring site after a break of longer than a year. The participant did not receive any infusion from the second site. The Call Center was not able to locate the participant for any follow-up calls. This participant will be censored at the time of lost to follow-up at the first site for any analyses using the ITT or mITT populations.

Appendix III. TACT2 Statistical Team

Team	Name	Email	Attends DSMB Meetings
**Blinded Statistical Team**	Hayley Nemeth	Hayley.nemeth@duke.edu	X
	Jun Wen	Jun.Wen@duke.edu	X
	Zhen Huang	zhen.huang@duke.edu	X
	Hwasoon Kim	hwasoon.kim@duke.edu	X (left DCRI in June, 2022)
	Kevin Anstrom	kevin.anstrom@unc.edu	X
**Unblinded Statistical Team**	Yuliya Lokhnygina	yuliya.lokhnygina@duke.edu	X
	Dianne Gallup	dianne.gallup@duke.edu	X
	Peter Merrill	Peter.merrill@duke.edu	X (left DCRI in October, 2022
**Metals Manuscript**	Zhen Huang	zhen.huang@duke.edu	X
	Steve McNulty	steven.mcnulty@duke.edu	
	Peter Merrill	Peter.merrill@duke.edu	X (left DCRI in October, 2022)
	Yuliya Lokhnygina	yuliya.lokhnygina@duke.edu	X
**NHLBI Biostatistician**	Mario Stylianou	stylianm@nhlbi.nih.gov	X
**TACT2 Project Leaders**	Shelby Morgan	Shelby.morgan@duke.edu	X
	Anne-Marie Elliott	Ann.elliott@duke.edu	X
	Ana Mon	Ana.Mon@msmc.com	X
	Leigh Gosnell	Leigh.gosnell@duke.edu	X (Prior Project Leader)
	Wanda Parker	Wanda.parker@duke.edu	X (Prior Project Leader)

TACT2_SAP_primary_manuscript_V2.0_2023_09_12

Final Audit Report 2023-09-25

Created:	2023-09-19
By:	Hayley Nemeth (hn58@duke.edu)
Status:	Signed
Transaction ID:	CBJCHBCAABAAAUNiGrumb3uY8zI_vHj6RCHwHfRwUk39

"TACT2_SAP_primary_manuscript_V2.0_2023_09_12" History

Document created by Hayley Nemeth (hn58@duke.edu)

2023-09-19 - 5:03:57 PM GMT- IP address: 136.226.51.16

Document emailed to hayley.nemeth@duke.edu for signature

2023-09-19 - 5:05:19 PM GMT

Email viewed by hayley.nemeth@duke.edu

2023-09-19 - 5:05:45 PM GMT- IP address: 136.226.51.16

Signer hayley.nemeth@duke.edu entered name at signing as Hayley Nemeth

2023-09-19 - 5:06:03 PM GMT- IP address: 136.226.51.16

Document e-signed by Hayley Nemeth (hayley.nemeth@duke.edu)

Signing reason: Approved

Signature Date: 2023-09-19 - 5:06:05 PM GMT - Time Source: server- IP address: 136.226.51.16

Document emailed to kevin.anstrom@unc.edu for signature

2023-09-19 - 5:06:07 PM GMT

Email viewed by kevin.anstrom@unc.edu

2023-09-19 - 10:28:55 PM GMT- IP address: 152.2.57.199

Signer kevin.anstrom@unc.edu entered name at signing as Kevin J. Anstrom

2023-09-19 - 10:29:14 PM GMT- IP address: 152.2.57.199

Document e-signed by Kevin J. Anstrom (kevin.anstrom@unc.edu)

Signing reason: Approved

Signature Date: 2023-09-19 - 10:29:16 PM GMT - Time Source: server- IP address: 152.2.57.199

Document emailed to gervasio.lamas@msmc.com for signature

2023-09-19 - 10:29:19 PM GMT

Email viewed by gervasio.lamas@msmc.com

2023-09-21 - 8:41:28 PM GMT- IP address: 198.179.5.251

Signer gervasio.lamas@msmc.com entered name at signing as Gervasio Lamas

2023-09-21 - 8:42:23 PM GMT- IP address: 198.179.5.251

Document e-signed by Gervasio Lamas (gervasio.lamas@msmc.com)

Signing reason: Approved

Signature Date: 2023-09-21 - 8:42:25 PM GMT - Time Source: server- IP address: 198.179.5.251

Document emailed to daniel.mark@duke.edu for signature

2023-09-21 - 8:42:27 PM GMT

Email viewed by daniel.mark@duke.edu

2023-09-21 - 8:45:46 PM GMT- IP address: 104.28.39.144

Email viewed by daniel.mark@duke.edu

2023-09-22 - 10:10:29 PM GMT- IP address: 76.182.14.163

Email viewed by daniel.mark@duke.edu

2023-09-23 - 10:03:56 PM GMT- IP address: 76.182.14.163

Signer daniel.mark@duke.edu entered name at signing as Daniel Mark

2023-09-23 - 10:08:11 PM GMT- IP address: 76.182.14.163

Document e-signed by Daniel Mark (daniel.mark@duke.edu)

Signing reason: Approved

Signature Date: 2023-09-23 - 10:08:13 PM GMT - Time Source: server- IP address: 76.182.14.163

Document emailed to stylianm@nhlbi.nih.gov for signature

2023-09-23 - 10:08:14 PM GMT

Email viewed by stylianm@nhlbi.nih.gov

2023-09-23 - 10:18:51 PM GMT- IP address: 128.231.125.178

Email viewed by stylianm@nhlbi.nih.gov

2023-09-25 - 3:44:35 AM GMT- IP address: 172.226.66.107

Signer stylianm@nhlbi.nih.gov entered name at signing as Mario Stylianou

2023-09-25 - 4:07:23 PM GMT- IP address: 108.51.245.87

Document e-signed by Mario Stylianou (stylianm@nhlbi.nih.gov)

Signing reason: Approved

Signature Date: 2023-09-25 - 4:07:25 PM GMT - Time Source: server- IP address: 108.51.245.87

Agreement completed.

2023-09-25 - 4:07:25 PM GMT

Adobe Acrobat Sign
