# Supplementary material for: Multivitamins After Myocardial Infarction in Patients With Diabetes: A Randomized Clinical Trial
Source: JAMA Intern Med. 2025 Mar 3;185(5):540–8. doi: 10.1001/jamainternmed.2024.8408 (PMC11877407; doi:10.1001/jamainternmed.2024.8408)
Supplement: Supplement 2. — eTable 1. Demographics and Baseline Clinical Characteristics for the 4 Treatment Groups (in the Primary Analysis Population) eFigure 1. A, Active OMVM vs Placebo OMVM: Subgroup Analysis of the Primary Outcome (in the Primary Analysis Population); B, Active OMVM vs Placebo OMVM: Subgroup Analysis of the Primary Outcome: Statins (in the Primary Analysis Population) eTable 2. 4 Treatment Group Analysis: Primary and Key Secondary End Points (in the Primary Analysis Population) eFigure 2. Cumulative Incidence of Time to First Event: Myocardial Infarction, Stroke, Hospitalization for Unstable Angina, Coronary Revascularization, or Death from Any Cause by 4 Treatment Groups (in the Primary Analysis Population) eTable 3. Participant OMVM Intervention Exposure Over Time (in the Primary Analysis Population) eFigure 3. Screening, Randomization, and Follow-Up (Screening Population) eFigure 4. Preinfusion Metal Levels at Baseline and Infusion Period for Urine Lead (µg of metal/g of creatinine), Blood Lead (µg/L), Urine Cadmium (µg of metal/g of creatinine), Blood Cadmium (µg/L), in the Primary Analysis Population eFigure 5. Preinfusion Metal Levels at Baseline and Infusion Period for Urine Molybdenum (µg of metal/g of creatinine), Blood Selenium (µg/L), and Blood Manganese (µg/L) (in the Primary Analysis Population) eTable 4. Overview of Serious Adverse Nonoutcome Events (Among Randomized Participants with One or More Infusion) [file jamainternmed-e248408-s002.pdf]

Supplemental Online Content

Ujueta F, Lamas GA, Anstrom KJ, et al; for the TACT2 Investigators. Multivitamins After Myocardial Infarction in Patients With Diabetes. *JAMA Intern Med.* Published online March 3, 2025. doi:10.1001/jamainternmed.2024.8408

eTable 1. Demographics and Baseline Clinical Characteristics for the 4 Treatment Groups (in the Primary Analysis Population)

eFigure 1. A, Active OMVM vs Placebo OMVM: Subgroup Analysis of the Primary Outcome (in the Primary Analysis Population); B, Active OMVM vs Placebo OMVM: Subgroup Analysis of the Primary Outcome: Statins (in the Primary Analysis Population)

eTable 2. 4 Treatment Group Analysis: Primary and Key Secondary End Points (in the Primary Analysis Population)

eFigure 2. Cumulative Incidence of Time to First Event: Myocardial Infarction, Stroke, Hospitalization for Unstable Angina, Coronary Revascularization, or Death from Any Cause by 4 Treatment Groups (in the Primary Analysis Population)

eTable 3. Participant OMVM Intervention Exposure Over Time (in the Primary Analysis Population)

eFigure 3. Screening, Randomization, and Follow-Up (Screening Population)

eFigure 4. Preinfusion Metal Levels at Baseline and Infusion Period for Urine Lead (µg of metal/g of creatinine), Blood Lead (µg/L), Urine Cadmium (µg of metal/g of creatinine), Blood Cadmium (µg/L), in the Primary Analysis Population

eFigure 5. Preinfusion Metal Levels at Baseline and Infusion Period for Urine Molybdenum (µg of metal/g of creatinine), Blood Selenium (µg/L), and Blood Manganese (µg/L) (in the Primary Analysis Population)

eTable 4. Overview of Serious Adverse Nonoutcome Events (Among Randomized Participants with One or More Infusion)

This supplemental material has been provided by the authors to give readers additional information about their work.

© 2025 Ujueta F et al. *JAMA Internal Medicine*.

eTable 1. Demographics and Baseline Clinical Characteristics for the 4 Treatment Groups (in the Primary Analysis Population)

Characteristic	EDTA Chelation + Active OMVM (N=250)	EDTA Chelation + Placebo OMVM (N=249)	Placebo Infusion + Active OMVM (N=250)	Placebo Infusion + Placebo OMVM (N=251)
Demographics				
Age - years				
Median (IQR)	66 (59 - 72)	67 (61 - 72)	67 (61 - 72)	67 (61 - 72)
Sex - no. / total no. (%)				
Female	61 (24.4)	79 (31.7)	75 (30.0)	55 (21.9)
Male	189 (75.6)	170 (68.3)	175 (70.0)	196 (78.1)
Race1 - no. / total no. (%)				
American Indian or Alaska Native	0 (0.0)	1 (0.4)	3 (1.2)	1 (0.4)
Asian	15 (6.0)	11 (4.4)	12 (4.8)	16 (6.4)
Black / African American	34 (13.6)	22 (8.8)	19 (7.6)	26 (10.4)
Multi-race	1 (0.4)	3 (1.2)	1 (0.4)	1 (0.4)
Native Hawaiian or other Pacific Islander	5 (2.0)	3 (1.2)	5 (2.0)	5 (2.0)
White / Caucasian	186 (74.4)	194 (77.9)	204 (81.6)	194 (77.3)
Other	9 (3.6)	15 (6.0)	6 (2.4)	8 (3.2)
Ethnicity- no. / total no. (%)				
Hispanic or Latino	46 / 246 (18.7)	53 / 244 (21.7)	53 (21.2)	46 / 247 (18.6)
Not Hispanic or Latino	200/ 246 (81.3)	191 / 244 (78.3)	197 (78.8)	201/247 (81.4)
Medical History2 - no. / total no. (%)				
Diabetes				
Type I	11 (4.4)	7 (2.8)	15 (6.0)	7 (2.8)
Type II	239 (95.6)	242 (97.2)	235 (94.0)	244 (97.2)
Time from diabetes diagnosis to randomization - years3				
Median (IQR)	13 (7 - 20)	15 (8 - 21)	15 (7 - 24)	14 (7 - 21)
Time from qualifying MI to randomization - years				
Median (IQR)	5 (2 - 9)	5 (2 - 10)	5 (2 - 11)	5 (2 - 10)
Anterior MI	71 (28.4)	73 (29.3)	80 (32.0)	86 (34.3)
Congestive heart failure	50 (20.0)	63 (25.3)	48 (19.2)	46 (18.3)
Stroke	28 / 249 (11.2)	21 / 247 (8.5)	24 / 248 (9.7)	20 (8.0)

© 2025 Ujueta F et al. *JAMA Internal Medicine*.

Peripheral vascular disease	29 / 245 (11.8)	40 / 243 (16.5)	47 / 245 (19.2)	44 / 247 (17.8)
Hypertension (requiring treatment)	228 (91.2)	226 (90.8)	231 / 249 (92.8)	233 (92.8)
Hypercholesterolemia4	231 / 249 (92.8)	221 / 247 (89.5)	224 / 247 (90.7)	228 (90.8)
Any cardiac revascularization (CABG or PCI)	209 / 248 (84.3)	198 (79.5)	199 / 248 (80.2)	207 / 250 (82.8)
Complications of Diabetes[5]	114/249 (45.8)	97/248 (39.1)	129/249 (51.8)	113/247 (45.7)
Cardiovascular Medications - no. / total no. (%)				
Aspirin, warfarin, or P2Y12 inhibitor	223 (89.2)	224 (90.0)	225 / 248 (90.7)	225 (89.6)
Statin	210 (84.0)	220 (88.4)	209 / 249 (83.9)	221 (88.0)
Beta-blocker	196 / 249 (78.7)	191 (76.7)	197 / 249 (79.1)	209 (83.3)
Angiotensin converting enzyme inhibitor or angiotensin receptor blocker	157 (62.8)	168 (67.5)	156 / 248 (62.9)	155 (61.8)
PCSK9 inhibitor	14 / 249 (5.6)	3 (1.2)	10 / 247 (4.0)	2 (0.8)
Diabetes Medications - no. / total no. (%)				
Insulin	117 (46.8)	125 (50.2)	122 (48.8)	104 (41.4)
Other oral agents	165 (66.0)	174 (69.9)	168/248 (67.7)	176 (70.1)
GLP-receptor agonist or SGLT-2 inhibitor	51 (20.4)	53 (21.3)	62 / 248 (25.0)	56 / 249 (22.5)
DPP-4 inhibitor	30 (12.0)	25 (10.0)	28 / 246 (11.4)	34 / 250 (13.6)
Other diabetes medications	16 / 244 (6.6)	16 / 248 (6.5)	21 / 247 (8.5)	15 / 248 (6.0)
No diabetes medications	15 / 247 (6.1)	12 (4.8)	8 (3.2)	18 / 250 (7.2)
Vitals				
BMI - kg/m2				
Median (IQR)	31.5 (28.2 – 36.5)	31.6 (28.4 – 35.6)	32.2 (28.0 – 36.9)	31.4 (28.5 – 36.5)
Blood pressure				
Systolic				
Mean (SD)	132.3 ± 17.51	131.1 ± 16.88	133.0 ± 17.75	134.1 ± 17.72
Diastolic				
Mean (SD)	74.7 ± 9.77	73.2 ± 10.06	74.1 ± 11.03	74.4 ± 10.25

© 2025 Ujueta F et al. *JAMA Internal Medicine*.

Laboratory examinations – Median (IQR)				
Fasting glucose, mg/dL	136.0 (113.0 – 169.0)	133.0 (104.0 – 163.0)	138.0 (113.0 – 171.0)	137.0 (117.5 – 184.0)
Hemoglobin A1c, %	7.3 (6.5 – 8.4)	7.1 (6.4 – 8.3)	7.3 (6.5 – 8.2)	7.3 (6.6 – 8.5)
Creatinine, mg/dL	1.1 (0.9 – 1.2)	1.0 (0.8 – 1.2)	1.1 (0.9 – 1.3)	1.0 (0.9 – 1.3)
Calculated eGFR (mL/min/1.73m2)	68.3 (56.5 – 82.9)	70.5 (54.6 – 84.7)	65.1 (51.8 – 82.5)	71.4 (54.7 – 84.5)
HDL, mg/dL	41.0 (34.0 – 49.0)	41.0 (33.0 – 50.0)	42.0 (35.0 – 51.0)	40.0 (34.0 – 48.0)
LDL, mg/dL	75.0 (60.0 – 103.0)	72.0 (56.0 – 93.0)	69.0 (54.0 – 87.0)	73.0 (54.0 – 100.0)
Total cholesterol, mg/dL	146.5 (125.0 – 184.0)	145.0 (122.0 – 166.0)	144.0 (122.0 – 169.0)	142.0 (122.0 – 178.0)
Triglycerides, mg/dL	150.0 (103.0 – 233.0)	134.0 (100.0 – 194.0)	139.0 (104.0 – 201.0)	149.0 (102.0 – 226.0)
Blood and Urine Metals- Median (IQR)				
Lead (blood), µg/L	9.80 (6.50, 14.69)	8.40 (5.93, 13.50)	9.50 (6.41, 14.00)	9.20 (6.31, 14.00)
Cadmium (urine), µg/g[6]	0.27 (0.16, 0.50)	0.33 (0.20, 0.55)	0.30 (0.18, 0.49)	0.28 (0.18, 0.52)

1 Race collected by selecting all that apply as reported by the participant. If multiple races were selected, participant is included in the 'Multi-race' category.
2 Medical history is participant reported.
3 Year of diabetes diagnosis was collected. Day and month imputed as June 1 of the collected year.
4 Total cholesterol > 240 md/dL (6.2mmol/L); LDL > 130 mg/dL (3.4 mmol/L).
5 Laser/injection eye treatment for diabetic retinopathy, diabetic neuropathy, amputation, or hypoglycemia with help needed for treatment.
6 Baseline urinary metal levels (µg/g) corrected for urinary creatinine (µg of metal per g creatinine).

© 2025 Ujueta F et al. *JAMA Internal Medicine*.

eFigure 1a. Active OMVM vs. Placebo OMVM - Subgroup Analysis of the Primary Outcome (in the Primary Analysis Population)

Subject Group	No. of Event (%) Active OMVM+ (N=500)	Placebo OMVM+ (N=500)	Interaction P-value	Adjusted Hazard Ratio[1]	95% CI
**All Participants**	175/500 (35.0%)	175/500 (35.0%)		0.989	(0.802, 1.220)
**Sex**					
Female	41/136 (30.1%)	45/134 (33.6%)	0.312	0.817	(0.534, 1.250)
Male	134/364 (36.8%)	130/366 (35.5%)		1.052	(0.826, 1.339)
**Ethnicity**					
Hispanic/Latino	34/99 (34.3%)	35/99 (35.4%)	0.524	0.854	(0.532, 1.372)
Non-Hispanic/Latino	140/397 (35.3%)	138/392 (35.2%)		1.014	(0.802, 1.284)
**Race**					
White	138/390 (35.4%)	139/388 (35.8%)	0.236	0.968	(0.765, 1.226)
Black	17/53 (32.1%)	22/48 (45.8%)		0.726	(0.385, 1.370)
Other	20/57 (35.1%)	14/64 (21.9%)		1.610	(0.812, 3.189)
**Age**					
<= 70 Years	123/342 (36.0%)	103/327 (31.5%)	0.074	1.159	(0.891, 1.507)
> 70 Years	52/158 (32.9%)	72/173 (41.6%)		0.773	(0.540, 1.104)
**MI Location**					
Anterior MI	47/151 (31.1%)	56/159 (35.2%)	0.522	0.889	(0.603, 1.310)
Non-anterior MI	128/349 (36.7%)	119/341 (34.9%)		1.033	(0.805, 1.327)
**Insulin use at baseline**					
Yes	99/239 (41.4%)	90/229 (39.3%)	0.626	1.038	(0.779, 1.382)
No	76/261 (29.1%)	85/271 (31.4%)		0.934	(0.685, 1.274)
**GLP-receptor agonist or SGLT-2 inhibitor at baseline**					
Yes	37/113 (32.7%)	35/109 (32.1%)	0.804	1.038	(0.653, 1.649)
No	137/385 (35.6%)	140/391 (35.8%)		0.972	(0.767, 1.230)
**Peripheral Artery Disease at baseline**					
Yes	30/76 (39.5%)	40/84 (47.6%)	0.869	0.960	(0.597, 1.544)
No	142/414 (34.3%)	133/406 (32.8%)		1.004	(0.792, 1.273)

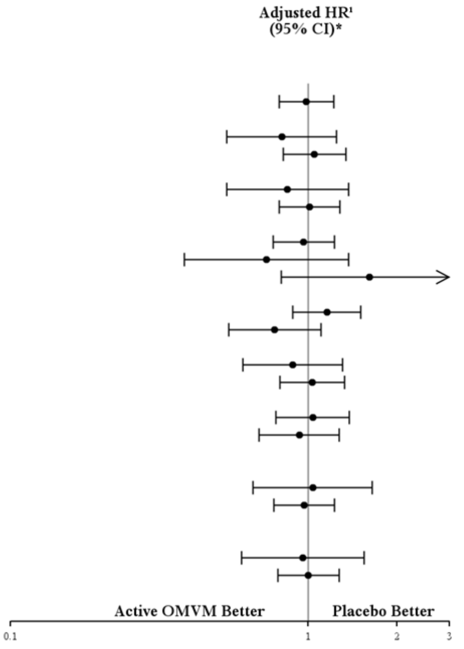

[+] Oral Multivitamins and Multiminerals.
1 Hazard ratio (Active OMVM vs. Placebo OMVM) from Cox proportional hazards regression with interaction between OMVM treatment and subgroup and adjustment of Chelation group, age, time-varying age, sex, and baseline insulin use. A hazard ratio < 1 indicates a benefit with Active OMVM compared to Placebo OMVM.
* The adjusted hazard ratios (95% CIs) are presented on the logarithmic scale.

eFigure 1b. Active OMVM vs. Placebo OMVM - Subgroup Analysis of the Primary Outcome - Statins (in the Primary Analysis Population)

© 2025 Ujueta F et al. *JAMA Internal Medicine*.

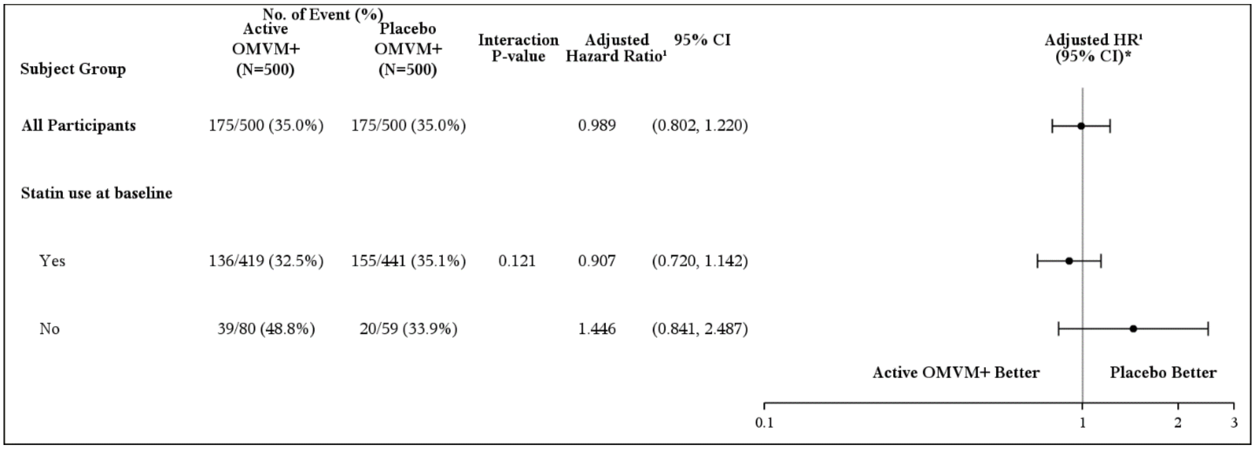

+ Oral Multivitamins and Multiminerals.
1 Hazard ratio (Active OMVM vs. Placebo OMVM) from Cox proportional hazards regression with interaction between OMVM treatment and subgroup and adjustment of Chelation group, age, time-varying age, sex, and baseline insulin use. A hazard ratio < 1 indicates a benefit with Active OMVM compared to Placebo OMVM.
* The adjusted hazard ratios (95% CIs) are presented on the logarithmic scale.

eTable 2. 4 Treatment Group Analysis - Primary and Key Secondary Endpoints (in the Primary Analysis Population)

Outcome	No. of Subjects with Event	Adjusted Hazard Ratio (95% CI)[1]	P value[2]
**Primary composite outcome**			
Time to first event: myocardial infarction, stroke, hospitalization for unstable angina, coronary revascularization, or death from any cause			
EDTA Chelation + Active OMVM	85 / 250 (34.0%)	0.910 (0.673, 1.230)	0.540
EDTA Chelation + Placebo OMVM	89 / 249 (35.7%)	0.968 (0.718, 1.305)	0.831
Placebo Chelation + Active OMVM	90 / 250 (36.0%)	1.040 (0.773, 1.399)	0.797
Placebo Chelation + Placebo OMVM	86 / 251 (34.3%)		

© 2025 Ujueta F et al. *JAMA Internal Medicine*.

Outcome	No. of Subjects with Event	Adjusted Hazard Ratio (95% CI)[1]	P value[2]
**Secondary composite outcome**			
Time to first event: myocardial infarction, stroke, or death from cardiovascular causes			
EDTA Chelation + Active OMVM	50 / 250 (20.0%)	1.152 (0.760, 1.745)	0.505
EDTA Chelation + Placebo OMVM	40 / 249 (16.1%)	0.913 (0.589, 1.415)	0.683
Placebo Chelation + Active OMVM	55 / 250 (22.0%)	1.330 (0.886, 1.996)	0.169
Placebo Chelation + Placebo OMVM	41 / 251 (16.3%)		
**Time to All-cause mortality**			
EDTA Chelation + Active OMVM	50 / 250 (20.0%)	0.939 (0.630, 1.398)	0.755
EDTA Chelation + Placebo OMVM	36 / 249 (14.5%)	0.687 (0.445, 1.060)	0.090
Placebo Chelation + Active OMVM	40 / 250 (16.0%)	0.774 (0.508, 1.179)	0.232
Placebo Chelation + Placebo OMVM	49 / 251 (19.5%)		

1 Hazard ratio (Active vs. Placebo Infusion + Placebo OMVM) from Cox proportional hazards regression with adjustment of age, (time-varying age for primary and secondary composite endpoints), sex, and baseline insulin use. A hazard ratio < 1 indicates a benefit with the active treatment compared to the reference group (Placebo Infusion + Placebo OMVM).

2 Type I error (two-sided α=0.05) is used as the threshold for hypothesis testing

© 2025 Ujueta F et al. *JAMA Internal Medicine*.

eFigure 2. Cumulative Incidence of Time to First Event: Myocardial Infarction, Stroke, Hospitalization for Unstable Angina, Coronary Revascularization, or Death from Any Cause by 4 Treatment Groups (in the Primary Analysis Population)

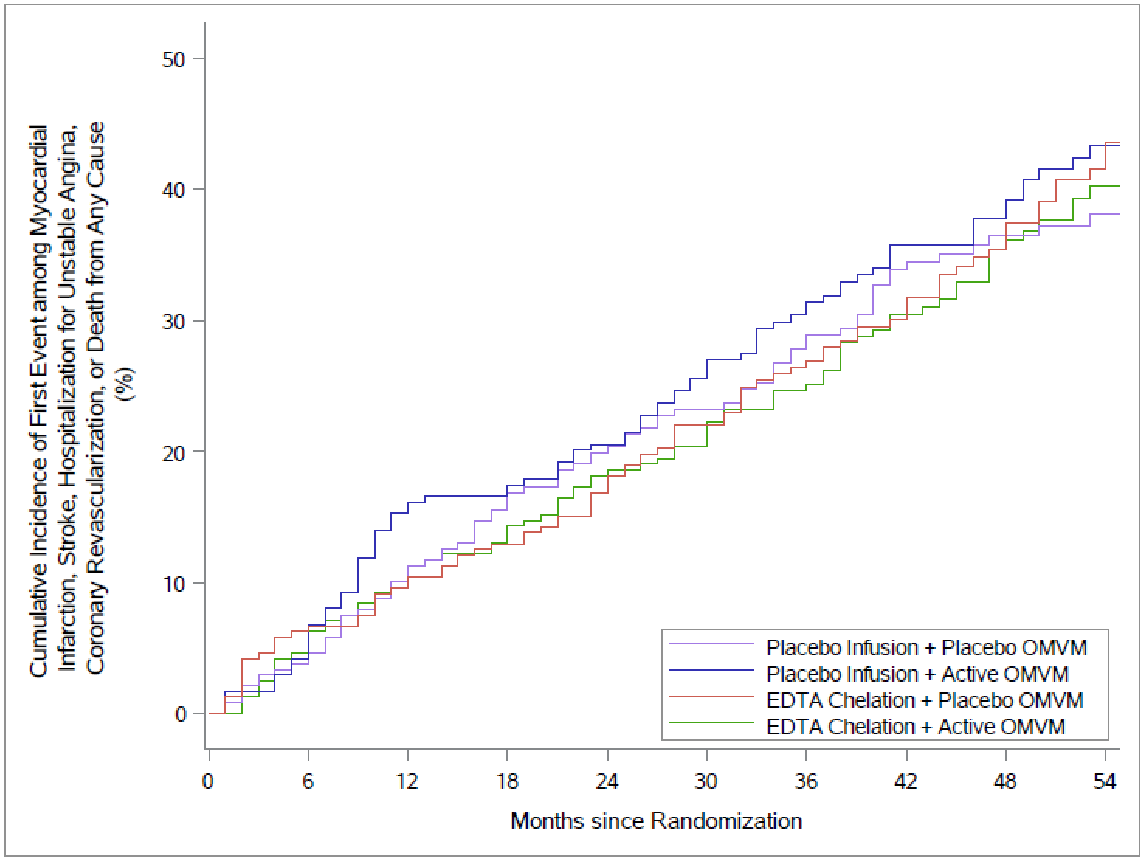

Note 1: Beyond month 54, the sample size for number at risk is lower than meaningful; therefore, the x-axis is displayed to 54 months.

eTable 3. Participant OMVM Intervention Exposure Over Time (in the Primary Analysis Population)

OMVM Compliance by Visit[1]	Active OMVM+ (N=500)	Placebo OMVM+ (N=500)	Total (N=1000)
Infusion Visit			

© 2025 Ujueta F et al. *JAMA Internal Medicine*.

OMVM Compliance by Visit[1]	Active OMVM[+] (N=500)	Placebo OMVM[+] (N=500)	Total (N=1000)
5	423 / 457 (92.6%)	402 / 437 (92.0%)	825 / 894 (92.3%)
10	411 / 436 (94.3%)	403 / 418 (96.4%)	814 / 854 (95.3%)
20	367 / 397 (92.4%)	372 / 387 (96.1%)	739 / 784 (94.3%)
30	340 / 371 (91.6%)	340 / 359 (94.7%)	680 / 730 (93.2%)
40	315 / 343 (91.8%)	311 / 335 (92.8%)	626 / 678 (92.3%)
Post-Infusion Follow-up (months)			
12	303 / 395 (76.7%)	325 / 405 (80.2%)	628 / 800 (78.5%)
24	173 / 340 (50.9%)	178 / 347 (51.3%)	351 / 687 (51.1%)
36	129 / 267 (48.3%)	121 / 269 (45.0%)	250 / 536 (46.6%)
48	74 / 194 (38.1%)	77 / 192 (40.1%)	151 / 386 (39.1%)
60	34 / 98 (34.7%)	33 / 111 (29.7%)	67 / 209 (32.1%)

[+] Oral Multivitamins and Multiminerals.

1 OMVM compliance is defined as participants taking one or more tablets of the intervention at the specified visit among those with non-missing data.

eFigure 3. Screening, Randomization, and Follow-Up (Screening Population)

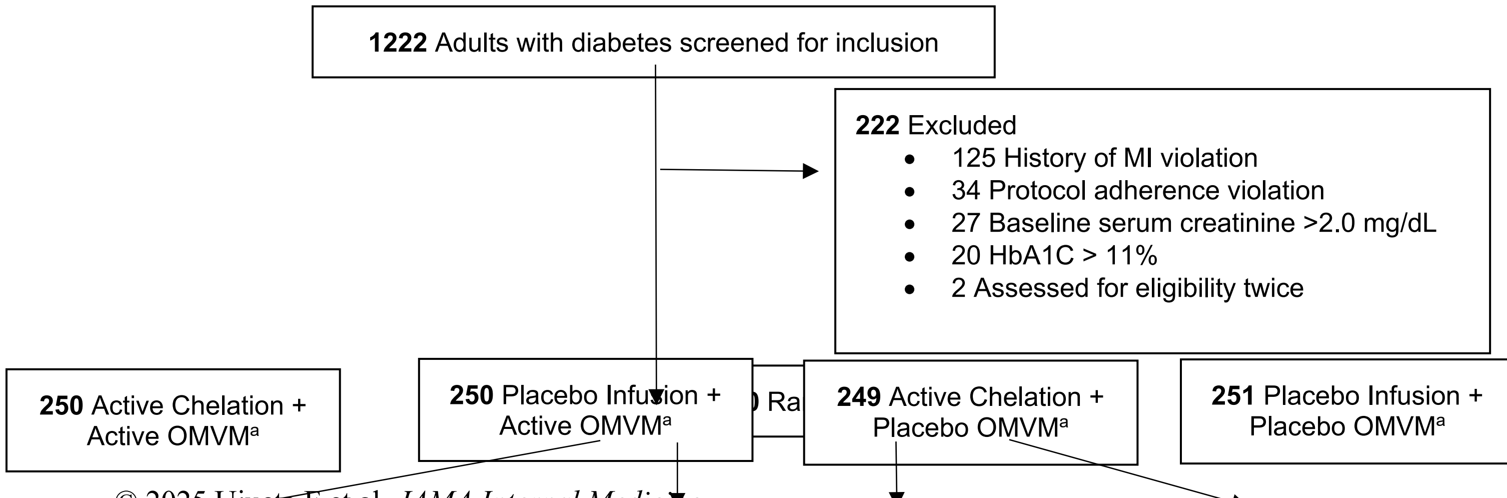

© 2025 Ujueta F et al. *JAMA Internal Medicine*.

500 Randomized to receive Active OMVM[a]

457 Initiated treatment as randomized

- 98 100% OMVM compliance[b]
- 116 80-99% OMVM compliance[b]
- 105 60-79% OMVM compliance[b]
- 79 40-59% OMVM compliance[b]
- 46 20-39% OMVM compliance[b]
- 13 1-19% OMVM compliance[b]

43 (9%) Did not initiate treatment

500 Randomized to receive Placebo OMVM[a]

448 Initiated treatment as randomized

- 76 100% OMVM compliance[b]
- 132 80-99% OMVM compliance[b]
- 114 60-79% OMVM compliance[b]
- 67 40-59% OMVM compliance[b]
- 46 20-39% OMVM compliance[b]
- 13 1-19% OMVM compliance[b]

52 (10%) Did not initiate treatment

a. High-dose oral multivitamins and multiminerals.
b. Oral Multivitamins and Multiminerals (OMVM) compliance is defined as the percentage of non-missing visits where the participant was taking one or more tablets of the intervention.
c. Randomized participants who did not die, were not lost to follow-up, and did not withdraw consent are considered as completing the study.
d. Participants with a death date prior to the end of study are included.
e. Lost to follow-up is defined as no contact within 12 months of the end of study. End of study is defined as the 5-year informed consent expiration date or administrative end of study date. Last contact is defined as the last known alive date.
f. All randomized participants are included in the primary analysis population.

© 2025 Ujueta F et al. *JAMA Internal Medicine*.

eFigure 4. Pre-Infusion Metal Levels at Baseline and Infusion Period for a) Urine Lead (µg of metal/g of creatinine), b) Blood Lead (µg/L), c) Urine Cadmium (µg of metal/g of creatinine), d) Blood Cadmium (µg/L), e) (in the Primary Analysis Population)

(a)

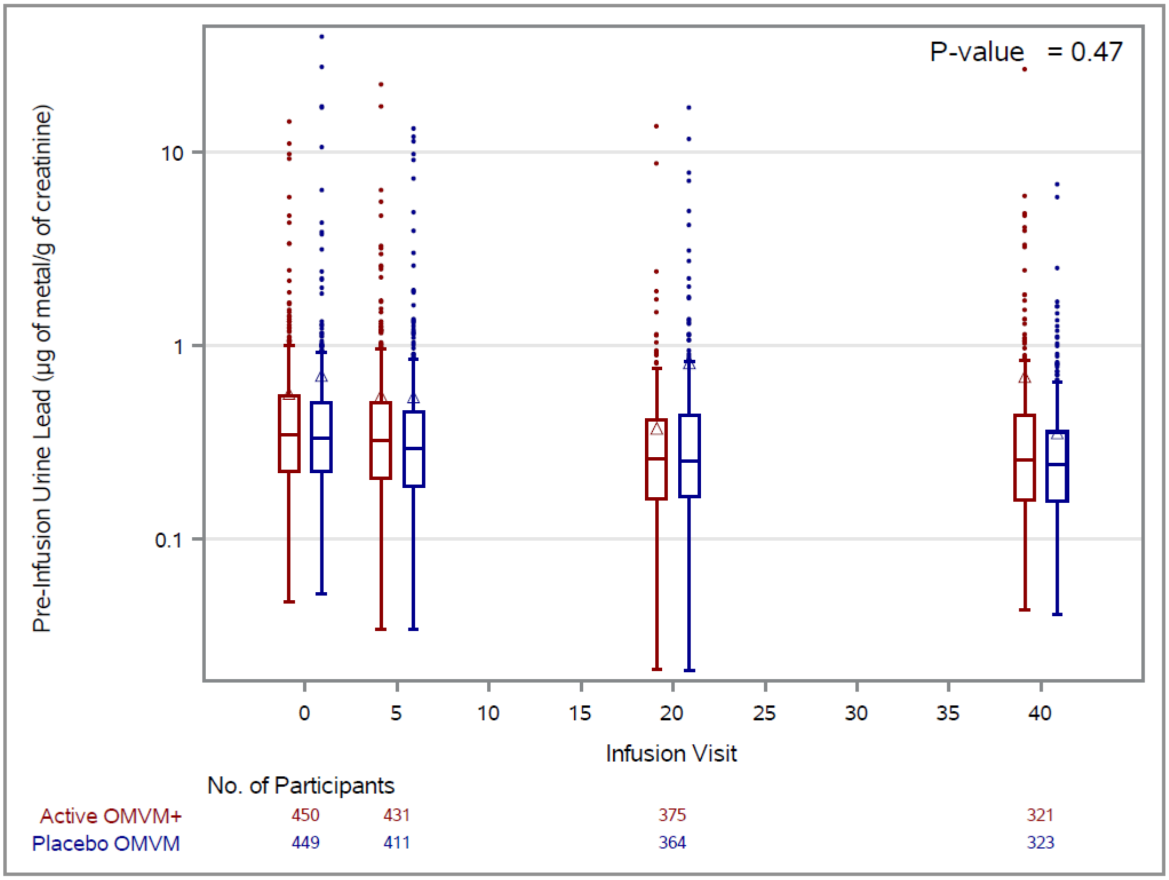

+ Oral Multivitamins and Multiminerals

Note 1: The change from baseline to pre-infusion 40 between active OMVM and placebo OMVM was tested using the Wilcoxon rank sums test (p-value = 0.47).

Note 2: The Y axis and X axis are offset to display both lines. The Y axis is on the logarithmic scale.

Note 3: The length of the box represents the interquartile range (the distance between the 25th and 75th percentiles). The triangle in the box interior represents the group mean. The horizontal line in the box interior represents the group median. Whiskers are drawn to 1.5 * IQR and small circles indicate suspected outliers.

Note 4: Outliers above the threshold for each figure were excluded (Figure 4a) 40; 2b) 100; 2c) 10; 2d) 10; 2e) 1,000; 2f) 500; 2g) 50).

b)

© 2025 Ujueta F et al. *JAMA Internal Medicine*.

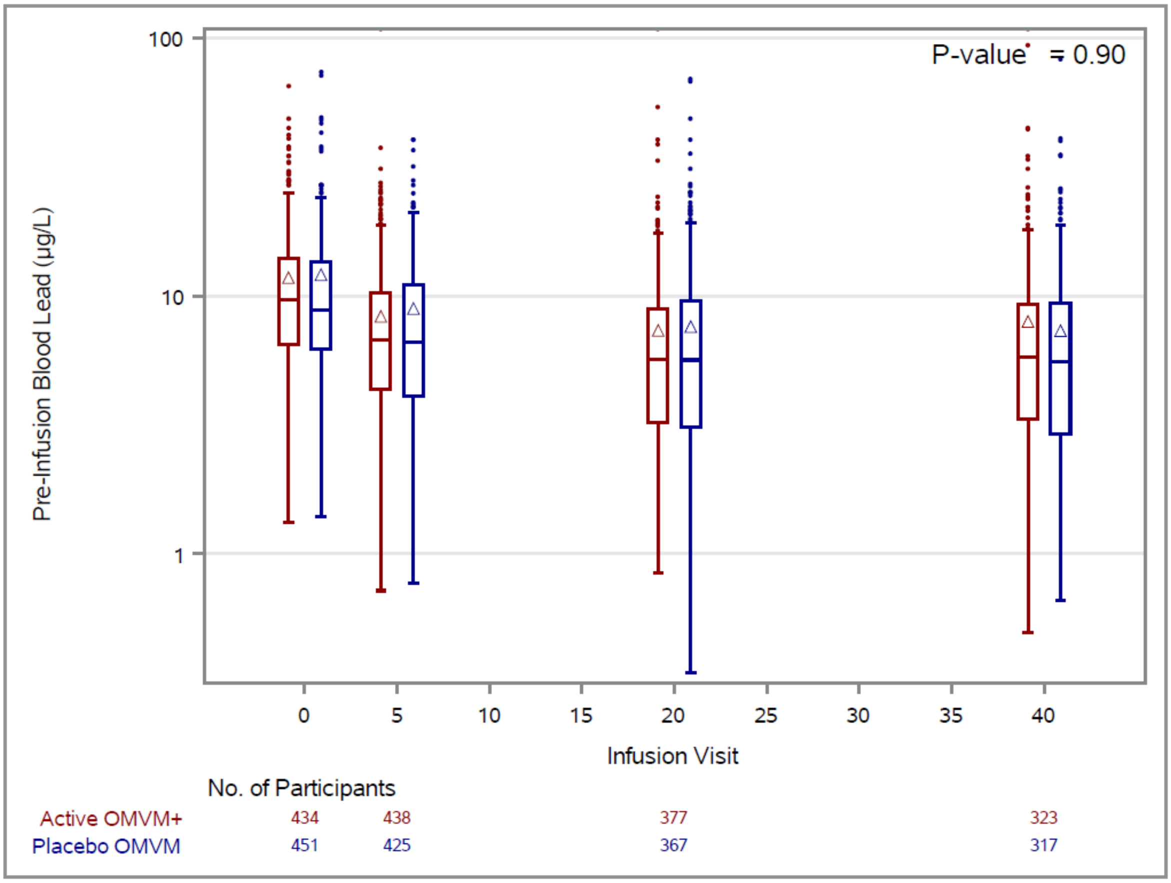

(c)

© 2025 Ujueta F et al. *JAMA Internal Medicine*.

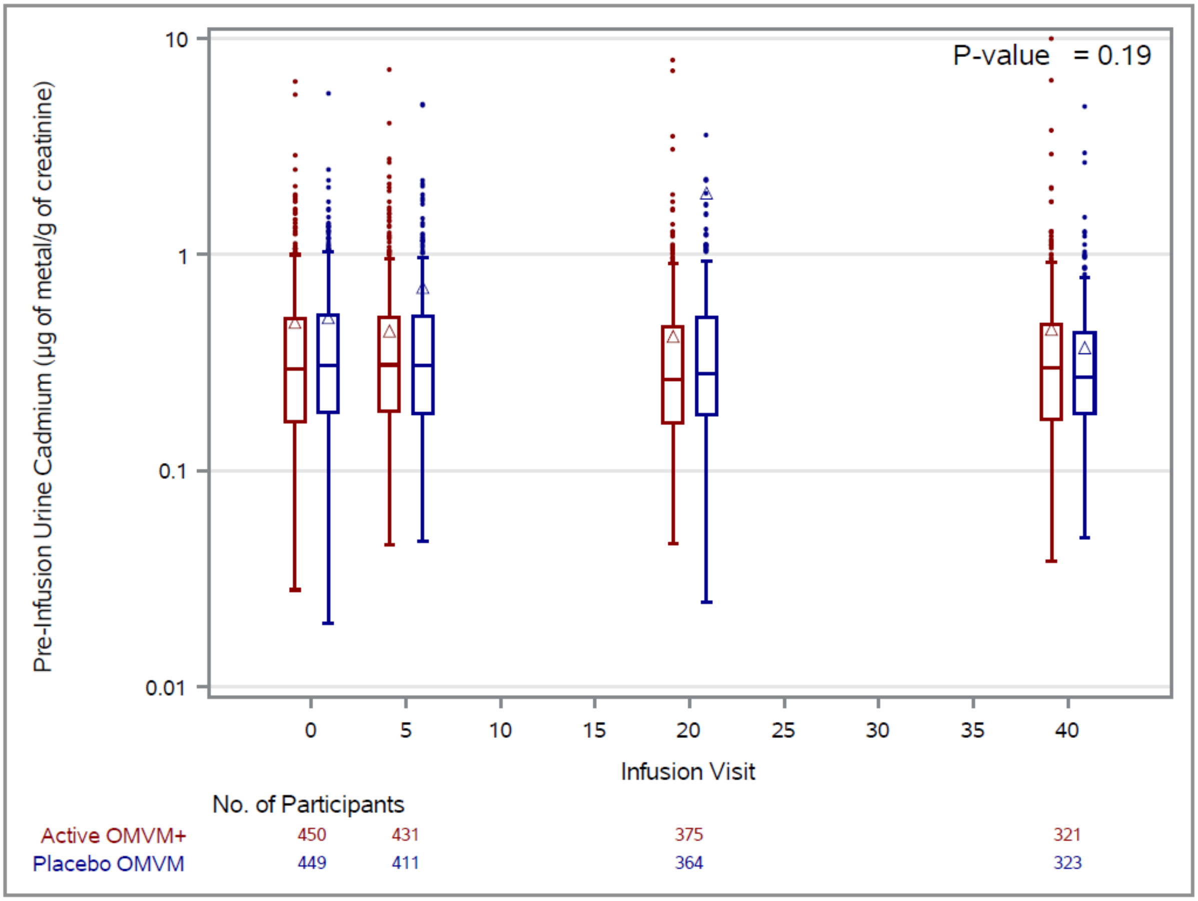

(d)

© 2025 Ujueta F et al. *JAMA Internal Medicine*.

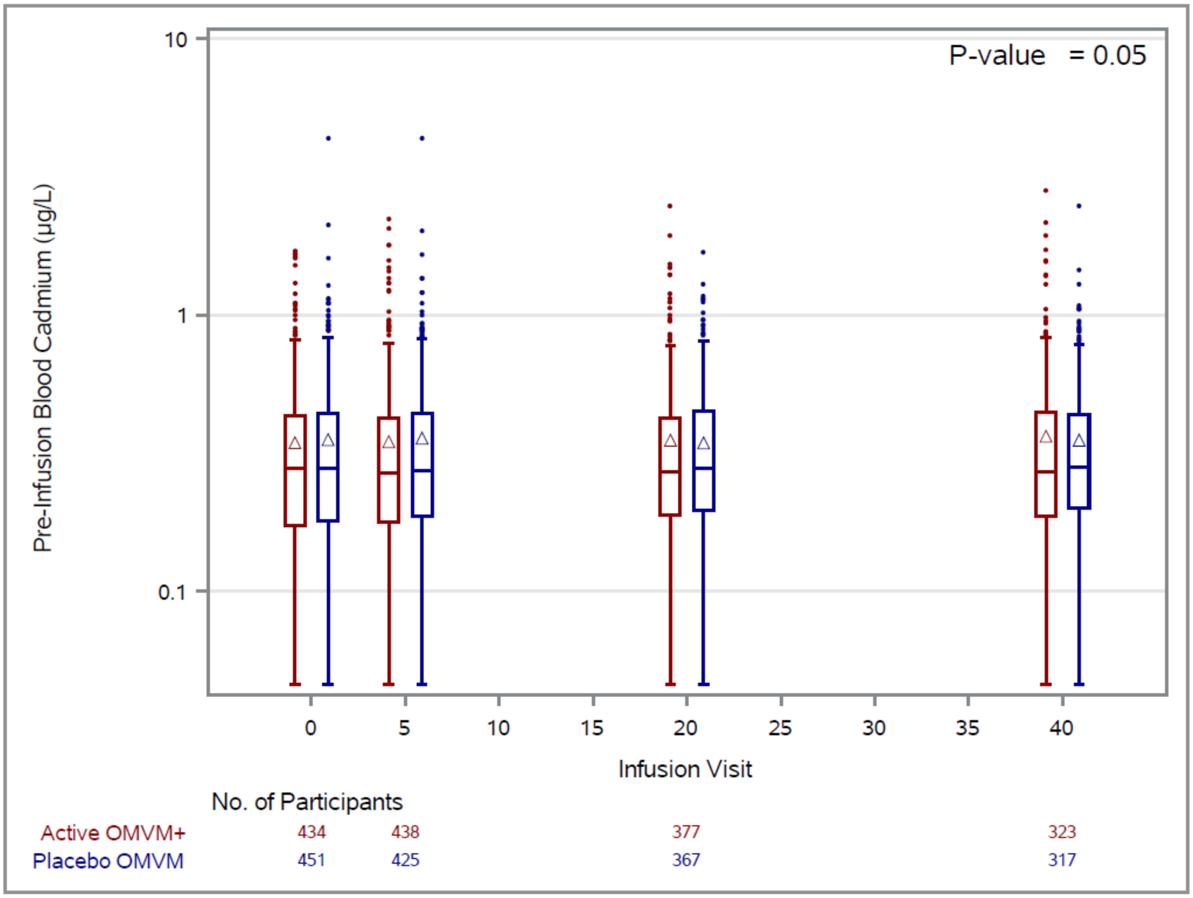

eFigure 5. Pre-Infusion Metal Levels at Baseline and Infusion Period for a) Urine Molybdenum (µg of metal/g of creatinine), b) Blood Selenium (µg/L), and c) Blood Manganese (µg/L) (in the Primary Analysis Population)

(a)

© 2025 Ujueta F et al. *JAMA Internal Medicine*.

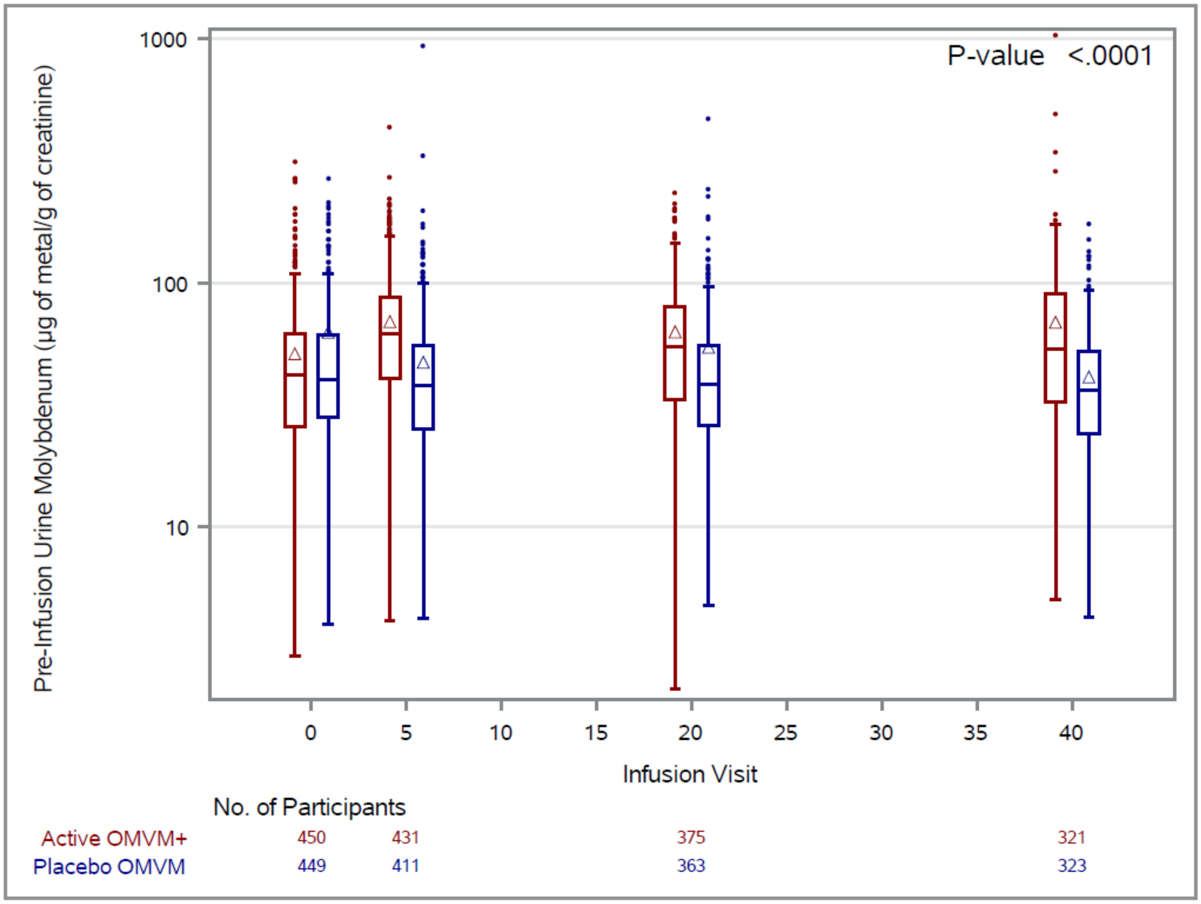

+ Oral Multivitamins and Multiminerals
Note 1: The change from baseline to pre-infusion 40 between active OMVM and placebo OMVM was tested using the Wilcoxon rank sums test (p-value = 0.47).
Note 2: The Y axis and X axis are offset to display both lines. The Y axis is on the logarithmic scale.
Note 3: The length of the box represents the interquartile range (the distance between the 25th and 75th percentiles). The triangle in the box interior represents the group mean. The horizontal line in the box interior represents the group median. Whiskers are drawn to 1.5 * IQR and small circles indicate suspected outliers.

(b)

© 2025 Ujueta F et al. *JAMA Internal Medicine*.

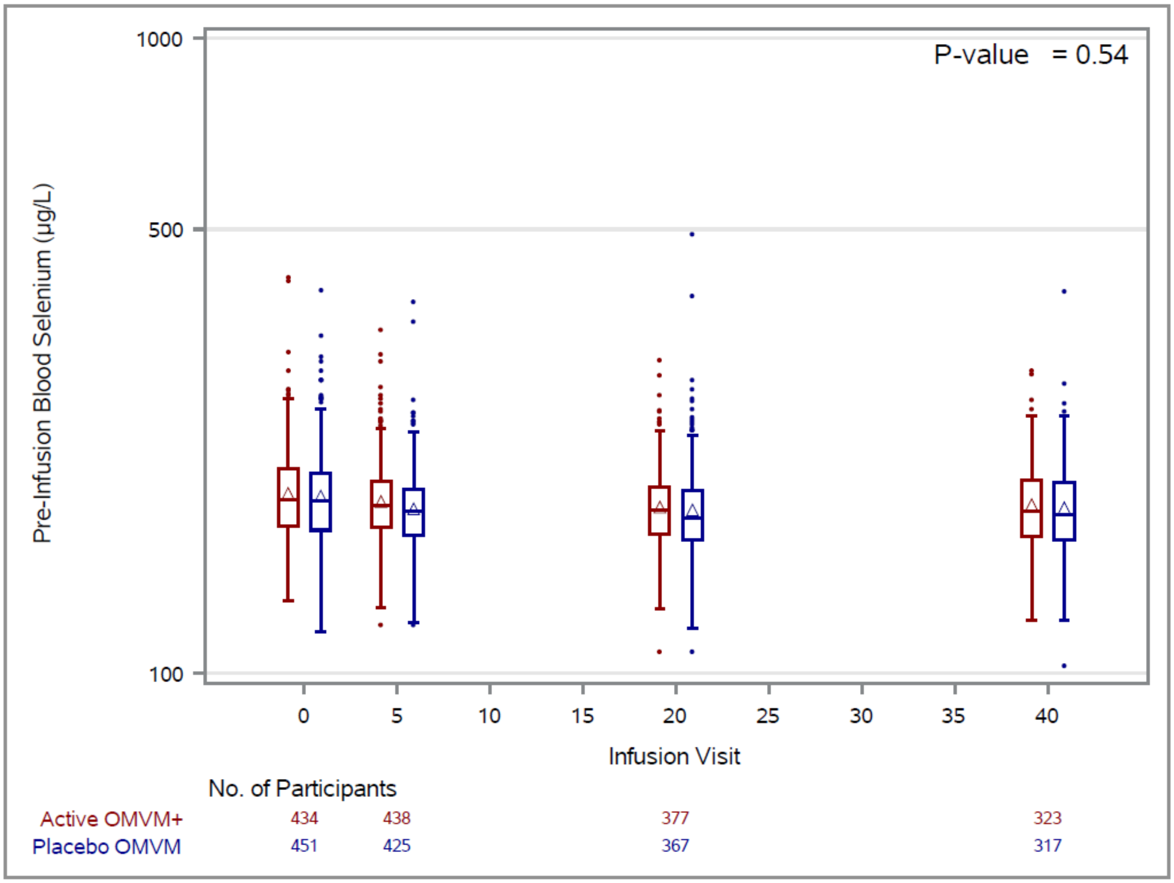

(c)

© 2025 Ujueta F et al. *JAMA Internal Medicine*.

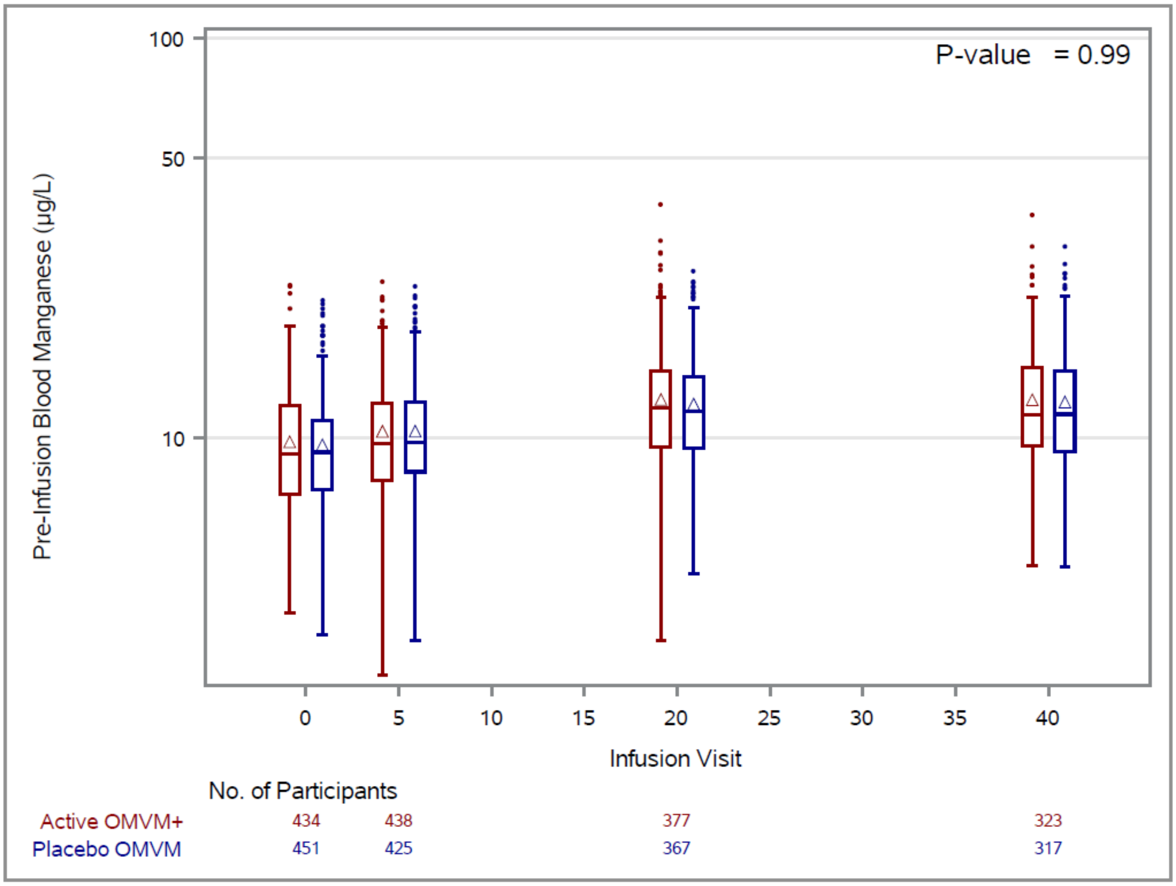

eTable 4. Overview of Serious Adverse Non-Outcome Events (Among Randomized Participants with One or More Infusion)

**Subjects with one or more:**	**Active OMVM+ (N=476)**	**Placebo OMVM+ (N=483)**	**All patients (N=959)**	**Risk Difference (95% CI)[1]**
Serious adverse event (SAE)	80 (16.8%)	80 (16.6%)	160 (16.7%)	-0.24% (-4.99%, 4.49%)

© 2025 Ujueta F et al. *JAMA Internal Medicine*.

Subjects with one or more:	Active OMVM+ (N=476)	Placebo OMVM+ (N=483)	All patients (N=959)	Risk Difference (95% CI)[1]
Severity / Intensity				
Mild	8 (1.7%)	11 (2.3%)	19 (2.0%)	0.60% (-1.28%, 2.55%)
Moderate	42 (8.8%)	34 (7.0%)	76 (7.9%)	-1.78% (-5.29%, 1.66%)
Severe	42 (8.8%)	45 (9.3%)	87 (9.1%)	0.49% (-3.19%, 4.18%)
SAE associated with study treatment	3 (0.6%)	1 (0.2%)	4 (0.4%)	-0.42% (-1.65%, 0.59%)
SAE resulting in death	6 (1.3%)	8 (1.7%)	14 (1.5%)	0.40% (-1.27%, 2.13%)
SAE required hospitalization	66 (13.9%)	73 (15.1%)	139 (14.5%)	1.25% (-3.23%, 5.73%)
Number of SAEs per subject				
1	60 (12.6%)	53 (11.0%)	113 (11.8%)	-1.63% (-5.77%, 2.47%)
2-3	16 (3.4%)	22 (4.6%)	38 (4.0%)	1.19% (-1.34%, 3.80%)
4-5	3 (0.6%)	5 (1.0%)	8 (0.8%)	0.40% (-0.92%, 1.85%)
>5	1 (0.2%)	0	1 (0.1%)	

+ Oral Multivitamins and Multiminerals.

1 95% confidence intervals for the risk difference were calculated with the use of the Miettinen-Nurminen method. Placebo is the reference group. Risk difference values smaller than 0 indicate a benefit with Active OMVM+.

Note: Percentages are calculated based on the total number of patients per treatment group. SAE time period is from randomization to 30 days post final infusion.

© 2025 Ujueta F et al. *JAMA Internal Medicine*.
