# Supplementary material for: Multivitamins After Myocardial Infarction in Patients With Diabetes: A Randomized Clinical Trial
Source: JAMA Intern Med. 2025 Mar 3;185(5):540–8. doi: 10.1001/jamainternmed.2024.8408 (PMC11877407; doi:10.1001/jamainternmed.2024.8408)
Supplement: Supplement 3. — TACT2 Nonauthor Collaborators [file jamainternmed-e248408-s003.pdf]

Supplemental Online Content: Nonauthor Collaborators

*Indicates required information. Only first name, last name, and suffix will appear in PubMed.

*Group Name(s): TACT2 Investigators							
***First Name and Middle Initial(s)**	***Last Name**	***Suffix (eg, Jr, III)**	Academic Degrees	Institution	Location (city, state/province, country)	Role or Contribution, eg, chair, principal investigator	Group (if more than 1 Group listed in the byline) and/or Subgroup (eg, Steering Committee)
Philip	Bear		MD	Iowa Heart Center	Wes Des Moines, IA	Principal Investigator	
Donna	Prouty			Iowa Heart Center	West Des Moines, IA	Study Coordinator	
Jodi	Baxter			Henry Ford Hospital	Detroit, MI	Study Coordinator	
Jonathan	Ehrman		PhD	Henry Ford Hospital	Detroit, MI	Principal Investigator	
Heather	Golden			Henry Ford Hospital	Detroit, MI	Study Coordinator	
Vikrant	Katoch			Comprehensive Cardiovascular Consultants	St. Louis, MO	Study Coordinator	
Raffi	Krikorian		MD	Comprehensive Cardiovascular Consultants	St. Louis, MO	Principal Investigator	
Andre	Paixao		MD	Arkansas Heart Hospital	Little Rock, AR	Principal Investigator	
Fujiko	Anazawa			Arkansas Heart Hospital	Little Rock, AR	Study Coordinator	
Leybi	Ramirez-Kelly			Arkansas Heart Hospital	Little Rock, AR	Study Coordinator	
Abby	Nolen			Arkansas Heart Hospital	Little Rock, AR	Study Coordinator	
Venus	Barney			Allergy and Environmental Medicine Center	Grand Blanc, MI	Study Coordinator	
Gerald	Natzke		MD	Allergy and Environmental Medicine Center	Grand Blanc, MI	Principal Investigator	
Rodica	Pop-Busui		MD	University of Michigan Medical Center	Ann Arbor, MI	Principal Investigator	
Cindy	Plunkett			University of Michigan Medical Center	Ann Arbor, MI	Study Coordinator	
Laura	Meyer			Comprehensive Heart Care, Inc.	Toledo, OH	Study Coordinator	
James	Roberts		MD	Comprehensive Heart Care, Inc.	Toledo, OH	Principal Investigator	
Scott	Rollins		MD	Advant Age Integrative Medicine	Grand Junction, CO	Principal Investigator	
Tamra	Hollis			Advant Age Integrative Medicine	Grand Junction, CO	Study Coordinator	
Nampalli	Vijay		MD	Aurora Denver Cardiology Associates, PC	Aurora, CO	Principal Investigator	
Melinda	Washam			Aurora Denver Cardiology Associates, PC	Aurora, CO	Study Coordinator	
David	Zidar		MD	University Hospitals Cleveland Medical Center	Cleveland, OH	Principal Investigator	
Terence	Semenec			University Hospitals Cleveland Medical Center	Cleveland, OH	Study Coordinator	

*Indicates required information. Only first name, last name, and suffix will appear in PubMed.

***First Name and Middle Initial(s)**	***Last Name**	***Suffix (eg, Jr, III)**	Academic Degrees	Institution	Location (city, state/province, country)	Role or Contribution, eg, chair, principal investigator	Group (if more than 1 Group listed in the byline) and/or Subgroup (eg, Steering Committee)
Lauren	Huntington			University Hospitals Cleveland Medical Center	Cleveland, OH	Study Coordinator	
Amanda	Klumpp			Upper Peninsula Holistic Medicine, PLC	Marquette, MI	Study Coordinator	
Matthew Scott	Doughty		MD	Upper Peninsula Holistic Medicine, PLC	Marquette, MI	Principal Investigator	
Jeffrey	Baker		MD	Clinical Research Prime	Idaho Falls, ID	Principal Investigator	
Joseph	Allen			Clinical Research Prime	Idaho Falls, ID	Study Coordinator	
Rebecca	Cortez			Clinical Research Prime	Idaho Falls, ID	Study Coordinator	
Bhaskar	Purushottam		MD	Monument Health Clinical Research	Rapid City, SD	Principal Investigator	
Kirstin	Stauffacher			Monument Health Clinical Research	Rapid City, SD	Study Coordinator	
Kelly	Airey		MD	Monument Health Clinical Research	Rapid City, SD	Principal Investigator	
Elena	Christofides		MD	Endocrinology Research Associates Inc.	Columbus, OH	Principal Investigator	
Jordyn	Conway			Endocrinology Research Associates Inc.	Columbus, OH	Study Coordinator	
David	Hoffman		MD	Saint Elizabeth Health Center	Youngstown, OH	Principal Investigator	
Patty	Schuler			Saint Elizabeth Health Center	Youngstown, OH	Study Coordinator	
Ronald	Solbrig		MD	Idaho State University	Pocatello, ID	Principal Investigator	
Loni	Chacon			Idaho State University	Pocatello, ID	Study Coordinator	
Phillip	Levy		MD	Wayne State University	Detroit, MI	Principal Investigator	
Linda	Gojcevic			Wayne State University	Detroit, MI	Study Coordinator	
Roy	Collins			Wayne State University	Detroit, MI	Study Coordinator	
John Miles	McClure II		MD	Mid Michigan Heart and Vascular Center	Saginaw, MI	Principal Investigator	
Ellen	Mook			Mid Michigan Heart and Vascular Center	Saginaw, MI	Study Coordinator	
Christopher	DeFilippi		MD	Inova Fairfax Hospital	Falls Church, VA	Principal Investigator	
Wendy	Sheaffer			Inova Fairfax Hospital	Falls Church, VA	Study Coordinator	
Antoinette	Bonaccorso			Mount Sinai Hospital	New York, NY	Study Coordinator	
Daniel	Donovan		MD	Mount Sinai Hospital	New York, NY	Principal Investigator	

Supplemental Online Content: Nonauthor Collaborators
*Indicates required information. Only first name, last name, and suffix will appear in PubMed.

*First Name and Middle Initial(s)	*Last Name	*Suffix (eg, Jr, III)	Academic Degrees	Institution	Location (city, state/province, country)	Role or Contribution, eg, chair, principal investigator	Group (if more than 1 Group listed in the byline) and/or Subgroup (eg, Steering Committee)
Julee	Hartwell			Insight Medical Research Corporation	Glens Falls, NY	Study Coordinator	
Andrew	Garner		MD	Insight Medical Research Corporation	Glens Falls, NY	Principal Investigator	
Dawn	Kalbfliesh			Insight Medical Research Corporation	Glens Falls, NY	Study Coordinator	
David	Hsi		MD	Stamford Hospital	Stamford, CT	Principal Investigator	
Maryanne	Ducey			Stamford Hospital	Stamford, CT	Study Coordinator	
Paula	Trump			Penn State University Cardiovascular Center	Hershey, PA	Study Coordinator	
Urs	Leuenberger		MD	Penn State University Cardiovascular Center	Hershey, PA	Principal Investigator	
Katie	Loffredo			Penn State University Cardiovascular Center	Hershey, PA	Study Coordinator	
Betty	Persico			Magaziner Center for Wellness	Cherry Hill, NJ	Study Coordinator	
Allan	Magaziner		MD	Magaziner Center for Wellness	Cherry Hill, NJ	Principal Investigator	
Dennis	Goodman		MD	New York University	New York, NY	Principal Investigator	
Michela	Garabedian			New York University	New York, NY	Study Coordinator	
Jane	Coates			New York University	New York, NY	Study Coordinator	
Jeanne	Wingo			Johns Hopkins Bayview Medical Center	Baltimore, MD	Study Coordinator	
Sheldon	Gottlieb		MD	Johns Hopkins Bayview Medical Center	Baltimore, MD	Principal Investigator	
Michael	Schachter		MD	Schachter Center for Complementary Medicine	Suffern, NY	Principal Investigator	
Sally	Minniefield			Schachter Center for Complementary Medicine	Suffern, NY	Study Coordinator	
Olakunle	Akinboboye		MD	Laurelton Heart Specialists PC	Rosedale, NY	Principal Investigator	
Kazi	Ullah			Laurelton Heart Specialists PC	Rosedale, NY	Study Coordinator	
Karen	Wolske			Adventist HealthCare Shady Grove Medical Center	Rockville, MD	Study Coordinator	
Dennis	Friedman		MD	Adventist HealthCare Shady Grove Medical Center	Rockville, MD	Principal Investigator	
Daniel	Lorber		MD	New York Hospital of Queens - Lang Research Center	Flushing, NY	Principal Investigator	

*Indicates required information. Only first name, last name, and suffix will appear in PubMed.

*First Name and Middle Initial(s)	*Last Name	*Suffix (eg, Jr, III)	Academic Degrees	Institution	Location (city, state/province, country)	Role or Contribution, eg, chair, principal investigator	Group (if more than 1 Group listed in the byline) and/or Subgroup (eg, Steering Committee)
Mari	Tsovian			New York Hospital of Queens - Lang Research Center	Flushing, NY	Study Coordinator	
Regina	Druz			New York Heart Reasearch Foundation	Mineoloa, NY	Study Coordinator	
Patricia	Hodnett			New York Heart Reasearch Foundation	Mineola, NY	Study Coordinator	
Stephanie	Meller		MD	MD Medical Research	Oxon Hill, MD	Principal Investigator	
Maria	Bartolome			MD Medical Research	Oxon Hill, MD	Study Coordinator	
Giselle	Cortez Vargas			Mount Sinai Medical Center	Miami Beach, FL	Study Coordinator	
Ian	Ergui			Mount Sinai Medical Center	Miami Beach, FL	Study Coordinator	
Francisco	Ujueta			Mount Sinai Medical Center	Miami Beach, FL	Study Coordinator	
Priscilla	Valls			Mount Sinai Medical Center	Miami Beach, FL	Study Coordinator	
Marnie	Lozada			Mount Sinai Medical Center	Miami Beach, FL	Study Coordinator	
Raul	Blanco			Mount Sinai Medical Center	Miami Beach, FL	Study Coordinator	
Ivan	Arenas		MD	Mount Sinai Medical Center	Miami Beach, FL	Principal Investigator	
Esteban	Escolar		MD	Mount Sinai Medical Center	Miami Beach, FL	Principal Investigator	
Xiaoou	Pan			Mount Sinai Medical Center	Miami Beach, FL	Study Coordinator	
Ilana	Seidel		MD	George Washington Center for Integrative Medicine	Washington, DC	Principal Investigator	
Melisa	Corado-Williams			George Washington Center for Integrative Medicine	Washington, DC	Study Coordinator	
Ana	Lamas		MD	Ana M Lamas, MD	Miami, FL	Principal Investigator	
Mariana	Viera-Navarro			Ana M Lamas, MD	Miami, FL	Study Coordinator	
Miguel	Trevino		MD	Innovative Research of West Florida, Inc.	Clearwater, FL	Principal Investigator	
Kimberly	Mai			Innovative Research of West Florida, Inc.	Clearwater, FL	Study Coordinator	
Rachel	Eidelman		MD	Bethesda Health City	Boynton Beach, FL	Principal Investigator	
Gabriela	Reyes			Bethesda Health City	Boynton Beach, FL	Study Coordinator	
Kelly	Brown			Intercoastal Medical Group	Sarasota, FL	Study Coordinator	
Ricardo	Yaryura		MD	Intercoastal Medical Group	Sarasota, FL	Principal Investigator	
Amy	Little			Intercoastal Medical Group	Sarasota, FL	Study Coordinator	
Heber	Varela		MD	Novel Clinical Research Center	Bellaire, TX	Principal Investigator	
Malu	Bienes			Novel Clinical Research Center	Bellaire, TX	Study Coordinator	
Robert	Jeanfreau		MD	MedPharmics, LLC	Metairie, LA	Principal Investigator	

Supplemental Online Content: Nonauthor Collaborators

*Indicates required information. Only first name, last name, and suffix will appear in PubMed.

*First Name and Middle Initial(s)	*Last Name	*Suffix (eg, Jr, III)	Academic Degrees	Institution	Location (city, state/province, country)	Role or Contribution, eg, chair, principal investigator	Group (if more than 1 Group listed in the byline) and/or Subgroup (eg, Steering Committee)
Paul	Neff			MedPharmics, LLC	Metairie, LA	Study Coordinator	
Mark	Thompson		MD	CaroMont Heart	Gastonia, NC	Principal Investigator	
Melody	Lineberger-Moore			CaroMont Heart	Gastonia, NC	Study Coordinator	
Donna	Miller			Ocala Research Institute, Inc.	Ocala, FL	Study Coordinator	
Rakesh	Prashad		MD	Ocala Research Institute, Inc.	Ocala, FL	Principal Investigator	
John	Schmedtje Jr		MD	Roanoke Heart Institute	Roanoke, VA	Principal Investigator	
Melissa	Shelton			Roanoke Heart Institute	Roanoke, VA	Study Coordinator	
Charles	Treasure II		MD	Cardiovascular Research of Knoxville	Knoxville, TN	Principal Investigator	
Ashley	Freel			Cardiovascular Research of Knoxville	Powell, TN	Study Coordinator	
Gerardo	Rojas		MD	Global Healthcare for Adults	Orlando, FL	Principal Investigator	
Damaris	Bruceles			Global Healthcare for Adults	Orlando, FL	Study Coordinator	
Gabriel	Uwaifo		MD	Ochsner Baptist Clinical Trials Unit	New Orleans, LA	Principal Investigator	
Veronica	Hixon- Calliet			Ochsner Baptist Clinical Trials Unit	New Orleans, LA	Study Coordinator	
Amber	Passini		MD	Biogenesis Medical Center	Landrum, SC	Principal Investigator	
Stacey	Melton			Biogenesis Medical Center	Landrum, SC	Study Coordinator	
Denise	Moreno			Vista Health Research	Miami, FL	Study Coordinator	
Antonio	Blanco		MD	Vista Health Research	Miami, FL	Principal Investigator	
Anisia	Otero		MD	Vista Health Research, LLC	Homestead, FL	Principal Investigator	
Mayra	Rodriguez			Vista Health Research, LLC	Homestead, FL	Study Coordinator	
Felix	Sogade		MD	Georgia Arrhythmia Consultants	Macon, GA	Principal Investigator	
Simisola	Oludare			Georgia Arrhythmia Consultants	Macon, GA	Study Coordinator	
Vivian	Fonseca		MD	Tulane University Health Science Center	New Orleans, LA	Principal Investigator	
Cynthia	Moreau			Tulane University Health Science Center	New Orleans, LA	Study Coordinator	
Sofia	Marquez			Tulane University Health Science Center	New Orleans, LA	Study Coordinator	
Phillip	Duncan		MD	Cardiac Health Management Network	Chester, VA	Principal Investigator	
Unice	Davis			Cardiac Health Management Network	Chester, VA	Study Coordinator	
Steven	Tang		MD	Lake Internal Medicine Associates	Eustis, FL	Principal Investigator	

*Indicates required information. Only first name, last name, and suffix will appear in PubMed.

***First Name and Middle Initial(s)**	***Last Name**	***Suffix (eg, Jr, III)**	Academic Degrees	Institution	Location (city, state/province, country)	Role or Contribution, eg, chair, principal investigator	Group (if more than 1 Group listed in the byline) and/or Subgroup (eg, Steering Committee)
Bonnie	DeMuth			Lake Internal Medicine Associates	Eustis, FL	Study Coordinator	
Donovan	Christie		MD	ANWAN Wellness Medical Center	Tucker, GA	Principal Investigator	
Lorin	Roberts			ANWAN Wellness Medical Center	Tucker, GA	Study Coordinator	
Amanda	Martin			Ocala Cardiovascular Center	Ocala, FL	Study Coordinator	
Lan	Luo		MD	Ocala Cardiovascular Center	Ocala, FL	Principal Investigator	
Summer	Waddle			Ocala Cardiovascular Center	Ocala, FL	Study Coordinator	
Shameka	Brown			LKN Clinical Trials, LLC	Cornelius, NC	Study Coordinator	
Salvatore	Bianco		MD	LKN Clinical Trials, LLC	Cornelius, NC	Principal Investigator	
Helmut	Steinberg		MD	University of Tennessee Health Science Center	Memphis, TN	Principal Investigator	
Amy	Bell			University of Tennessee Health Science Center	Memphis, TN	Study Coordinator	
Dharmendra	Patel		MD	Erlanger Medical Center	Chattanooga, TN	Principal Investigator	
Alison	Bailey		MD	Erlanger Medical Center	Chattanooga, TN	Principal Investigator	
Michelle	Rice			Erlanger Medical Center	Chattanooga, TN	Study Coordinator	
David	Herrington		MD	Wake Forest University Health Sciences	Winston Salem, NC	Principal Investigator	
Karen	Blinson			Wake Forest University Health Sciences	Winston Salem, NC	Study Coordinator	
Lynda	Doomy			Wake Forest University Health Sciences	Winston-Salem, NC	Study Coordinator	
David	Smith		MD	Monroe Biomedical Research	Monroe, NC	Principal Investigator	
Jenny	Norton			Monroe Biomedical Research	Monroe, NC	Study Coordinator	
Suvi	Gross			Monroe Biomedical Research	Monroe, NC	Study Coordinator	
Assad	Mouhaffel		MD	Clinical Trials of America	Monroe, LA	Principal Investigator	
Clinton	Guillory		MD	Clinical Trials of America	West Monroe, LA	Principal Investigator	
Samantha	Korn			Clinical Trials of America	West Monroe, LA	Study Coordinator	
Yamil Wady	Aude		MD	Heart and Vascular Specialists of South Texas	McAllen, TX	Principal Investigator	
Yirsa	Esparza			Heart and Vascular Specialists of South Texas	McAllen, TX	Study Coordinator	
Sonia	Leal			Heart and Vascular Specialists of South Texas	McAllen, TX	Study Coordinator	
Leidy	Abreu			Heart and Vascular Specialists of South Texas	McAllen, TX	Study Coordinator	

Supplemental Online Content: Nonauthor Collaborators

*Indicates required information. Only first name, last name, and suffix will appear in PubMed.

*First Name and Middle Initial(s)	*Last Name	*Suffix (eg, Jr, III)	Academic Degrees	Institution	Location (city, state/province, country)	Role or Contribution, eg, chair, principal investigator	Group (if more than 1 Group listed in the byline) and/or Subgroup (eg, Steering Committee)
Eric	Auerbach		MD	Oklahoma Heart Institute	Tulsa, OK	Principal Investigator	
Cindy	Huff			Oklahoma Heart Institute	Tulsa, OK	Study Coordinator	
Miriam	Brooks			Oklahoma Heart Institute	Tulsa, OK	Study Coordinator	
Marina	Johnson		MD	Institute of Endocrinology and Preventive Medicine	Dallas, TX	Principal Investigator	
Tammy	Allen			Institute of Endocrinology and Preventive Medicine	Dallas, TX	Study Coordinator	
Arthur	Berkson		MD	Integrative Medical Center	Las Cruces, NM	Principal Investigator	
Krystal	McAuliffe			Integrative Medical Center	Las Cruces, NM	Study Coordinator	
Holly	Little			Houston Heart and Vascular Associates	Kingwood, TX	Study Coordinator	
Raymond	Little		MD	Houston Heart and Vascular Associates	Kingwood, TX	Principal Investigator	
Jalal	Abbas		MD	Clinical Research Institute of Arizona	Sun City West, AZ	Principal Investigator	
Christina	Collard			Clinical Research Institute of Arizona	Sun City West, AZ	Study Coordinator	
Jamie	Foreman			Clinical Research Institute of Arizona	Sun City West, AZ	Study Coordinator	
Shelby	Richardson			Wichita Falls Heart Clinic	Wichita Falls, TX	Study Coordinator	
Andre	Desire		MD	Wichita Falls Heart Clinic	Wichita Falls, TX	Principal Investigator	
Ayham	Shneker		MD	San Antonio Premier Internal Medicine	San Antonio, TX	Principal Investigator	
Omar	Cordero			San Antonio Premier Internal Medicine	San Antonio, TX	Study Coordinator	
Piotr	Kunik			San Antonio Endovascular and Heart Institute	San Antonio, TX	Study Coordinator	
Radoslav	Kiesz		MD	San Antonio Endovascular and Heart Institute	San Antonio, TX	Principal Investigator	
Kathy	Sasser			Patrick A. Golden, MD	Fresno, CA	Study Coordinator	
Patrick	Golden		MD	Patrick A. Golden, MD	Fresno, CA	Principal Investigator	
Renee	Garden			Center for Optimum Health	Los Angeles, CA	Study Coordinator	
Allen	Green		MD	Center for Optimum Health	Los Angeles, CA	Principal Investigator	
Gordon	Fung		MD	University of California at San Francisco	San Francisco, CA	Principal Investigator	

Supplemental Online Content: Nonauthor Collaborators

*Indicates required information. Only first name, last name, and suffix will appear in PubMed.

*First Name and Middle Initial(s)	*Last Name	*Suffix (eg, Jr, III)	Academic Degrees	Institution	Location (city, state/province, country)	Role or Contribution, eg, chair, principal investigator	Group (if more than 1 Group listed in the byline) and/or Subgroup (eg, Steering Committee)
Keith	Uyemura			University of California at San Francisco	San Francisco, CA	Study Coordinator	
Sajad	Hamal			Los Angeles Biomedical Research Institute	Torrance, CA	Study Coordinator	
Matthew	Budoff		MD	Los Angeles Biomedical Research Institute	Torrance, CA	Principal Investigator	
Dinh	Dinh		MD	Advanced RX Clinical Research Group, Inc	Westminister, CA	Principal Investigator	
Jenny	Nguyen			Advanced RX Clinical Research Group, Inc	Westminister, CA	Study Coordinator	
Ashwini	Erande			Susan Samueli Integrative Health Institute	Costa Mesa, CA	Study Coordinator	
Kelly	Brink			Susan Samueli Integrative Health Institute	Costa Mesa, CA	Study Coordinator	
Shaista	Malik		MD	Susan Samueli Integrative Health Institute	Costa Mesa, CA	Principal Investigator	
Nicolas	Chronos		MD	Cardiology Care Clinics	Eatonton, GA	Principal Investigator	
Nicole	Maslanka			Cardiology Care Clinics	Eatonton, GA	Study Coordinator	
Philip	O'Donnell		MD	Selma Medical Associates	Winchester, VA	Principal Investigator	
Margaret Ann	Bittorf			Selma Medical Associates	Winchester, VA	Study Coordinator	
Rajesh	Garg		MD	University of Miami Diabetes Research Institute	Miami, FL	Principal Investigator	
Della	Matheson			University of Miami Diabetes Research Institute	Miami, FL	Study Coordinator	
Aldo	Martinez		MD	Unicardio Medical Center	Miami, FL	Principal Investigator	
Yasiel	Hernandez			Unicardio Medical Center	Miami, FL	Study Coordinator	
Walter Herbert	Haught		MD	Heart Center Research, LLC	Huntsville, AL	Principal Investigator	
Lisa	Eskridge			Heart Center Research, LLC	Huntsville, AL	Study Coordinator	
Naseem	Jaffrani		MD	Alexandria Cardiology Clinic	Alexandria, LA	Principal Investigator	
Melissa	Trimble			Alexandria Cardiology Clinic	Alexandria, LA	Study Coordinator	
James (Jamie)	Powell		MD	East Carolina University	Greenville, NC	Principal Investigator	
Winifred	Bryant			East Carolina University	Greenville, NC	Study Coordinator	
Nakeydia	Bryant			East Carolina University	Greenville, NC	Study Coordinator	
Shmuel	Bergman		MD	Canadian Center for Preventive Medicine	Toronto, Canada	Principal Investigator	

Supplemental Online Content: Nonauthor Collaborators

*Indicates required information. Only first name, last name, and suffix will appear in PubMed.

***First Name and Middle Initial(s)**	***Last Name**	***Suffix (eg, Jr, III)**	Academic Degrees	Institution	Location (city, state/province, country)	Role or Contribution, eg, chair, principal investigator	Group (if more than 1 Group listed in the byline) and/or Subgroup (eg, Steering Committee)
Rezelle	Libuit			Canadian Center for Preventive Medicine	Toronto, Canada	Study Coordinator	
Angelica	Bringas			Canadian Center for Preventive Medicine	Toronto, Canada	Study Coordinator	
Diana	Visentin		MD	South Simcoe Cardiac Services	Barrie, Canada	Principal Investigator	
Lori	Murphy			South Simcoe Cardiac Services	Barrie, Canada	Study Coordinator	
Adrienne	Junek		MD	Seekers Centre for Integrative Medcine	Ottawa, Canada	Principal Investigator	
Shadi	Nahas			Seekers Centre for Integrative Medcine	Ottawa, Canada	Study Coordinator	
Joseph	Berlingieri		MD	JBN Medical Diagnostic Services Inc.	Burlington, Canada	Principal Investigator	
Caroline	Urso			JBN Medical Diagnostic Services Inc.	Burlington, Canada	Study Coordinator	
Alireza	Moshiri			BC Diabetes Research	Vancouver, Canada	Study Coordinator	
Thomas	Elliott			BC Diabetes Research	Vancouver, Canada	Principal Investigator	
Marla	Inducil			BC Diabetes Research	Vancouver, Canada	Study Coordinator	
Wanda	Parker			Duke University	Durham, NC	Project Leader- DCC	
Ryan	Stults			Duke University	Durham, NC	Lead CRA- DCC	
Hwasoon	Kim			Duke University	Durham, NC	Biostatistician- DCC	
Beatriz	Acevedo			Mount Sinai Medical Center	Miami Beach, FL	Administrative Coordinator-CCC	
Nancy	LoIacono			Columbia University	New York, NY	Research Scientist- Trace Metals & Biorepository Center	
