# Supplementary material for: Multivitamins After Myocardial Infarction in Patients With Diabetes: A Randomized Clinical Trial
Source: JAMA Intern Med. 2025 Mar 3;185(5):540–8. doi: 10.1001/jamainternmed.2024.8408 (PMC11877407; doi:10.1001/jamainternmed.2024.8408)
Supplement: Supplement 4. — Data Sharing Statement [file jamainternmed-e248408-s004.pdf]

Data Sharing Statement

Ujueta. Multivitamins After Myocardial Infarction in Patients With Diabetes. *JAMA Intern Med*. Published March 03, 2025. doi:10.1001/jamainternmed.2024.8408

Data
Additional Information: NCT02733185
Data available: Yes
Data types: Deidentified participant data, Data dictionary
How to access data: https://biolincc.nhlbi.nih.gov
When available: beginning date: 03-08-2026

Supporting Documents
Document types: None

Additional Information
Who can access the data: Anyone requesting the data
Types of analyses: for any purpose
Mechanisms of data availability: without investigator support
